# Novel Seleno- and Thio-Urea Containing Dihydropyrrol-2-One Analogues as Antibacterial Agents

**DOI:** 10.3390/antibiotics10030321

**Published:** 2021-03-19

**Authors:** Shekh Sabir, Tsz Tin Yu, Rajesh Kuppusamy, Basmah Almohaywi, George Iskander, Theerthankar Das, Mark D. P. Willcox, David StClair Black, Naresh Kumar

**Affiliations:** 1 School of Chemistry, The University of New South Wales, Sydney, NSW 2052, Australia; s.sabir@student.unsw.edu.au (S.S.); tsztin.yu@unsw.edu.au (T.T.Y.); r.kuppusamy@unsw.edu.au (R.K.); georgeiskanderb@gmail.com (G.I.); d.black@unsw.edu.au (D.S.B.); 2 School of Optometry and Vision Science, The University of New South Wales, Sydney, NSW 2052, Australia; m.willcox@unsw.edu.au; 3 Department of Pharmaceutical Chemistry, College of Pharmacy, King Khalid University, Abha 61421, Saudi Arabia; bal-mohawe@kku.edu.sa; 4 Department of Infectious Diseases and Immunology, School of Medical Sciences, The University of Sydney, Sydney, NSW 2006, Australia; das.ashishkumar@sydney.edu.au

**Keywords:** antibiotic resistance, *Pseudomonas aeruginosa*, quorum-sensing inhibitors, dihydropyrrol-2-one, organoselenium compounds, antibacterial activity

## Abstract

The quorum sensing (QS) system in multi-drug-resistant bacteria such as *P. aeruginosa* is primarily responsible for the development of antibiotic resistance and is considered an attractive target for antimicrobial drug discovery. In this study, we synthesised a series of novel selenourea and thiourea-containing dihydropyrrol-2-one (DHP) analogues as LasR antagonists. The selenium DHP derivatives displayed significantly better quorum-sensing inhibition (QSI) activities than the corresponding sulphur analogues. The most potent analogue **3e** efficiently inhibited the *las* QS system by 81% at 125 µM and 53% at 31 µM. Additionally, all the compounds were screened for their minimum inhibitory concentration (MIC) against the Gram-positive bacterium *S. aureus*, and interestingly, only the selenium analogues showed antibacterial activity, with **3c** and **3e** being the most potent with a MIC of 15.6 µM.

## 1. Introduction

Antimicrobial resistance (AMR) is a continuously rising problem and a major challenging threat to the health of the global population. The Centers for Disease Control and Prevention (CDC) and World Health Organisation (WHO) have estimated that by 2050, millions of people could die due to resistant microbial infections, and this could lead to another pandemic [1,2]. The antimicrobial activities of conventional antibiotics is achieved through the disruption of bacterial cell walls, or inhibition of bacterial nucleic acid, protein or folate synthesis [3,4]. Consequently, there is an increased selective pressure build-up in bacteria, which causes the development of antibiotic resistance through a variety of mechanisms, including the formation of biofilms, the elimination of antibiotics by efflux pumps, target gene mutations and reduced cell permeability [5,6,7,8]. Hence, there is a pressing need to discover new classes of antimicrobial therapeutics with novel modes of action [9].

Quorum sensing (QS) is a cell-density-dependent communication system between bacterial cells that facilitates their collective behaviour in response to changes that occur in their environment [10]. Quorum-sensing signalling molecules, also known as autoinducers (AIs), are produced by many bacterial cells. At high bacterial density, these small signalling molecules communicate and coordinate their collective behaviour by regulating several activities such as biofilm formation, the production of virulence factors (pyocyanin, rhamnolipids, and pyoverdine), swarming motility, and antibiotic resistance by modulating the transcription of several genes [11,12].

Several types of QS systems are present in different bacteria. For instance, *Pseudomonas aeruginosa* possesses three major interconnected QS systems, namely *las, rhl* and *pqs*, which are differentiated based on the chemical nature of the associated autoinducers. These signalling molecules activate their respective cognate protein receptors (LasR, RhR, and PqsR), which subsequently upregulates the activities of multiple genes responsible for virulence and biofilm formation. In addition, these QS systems can modulate the activities of each other; for example, the activities of *rhl* and *pqs* systems are positively regulated by *las* [13]. The *las* system utilises an *N*-acyl-L-homoserine lactone (AHL) type of autoinducer, which binds and activates a LuxR-type transcriptional regulator receptor LasR. *P. aeruginosa* synthesises *N*-(3-oxododecanoyl)-L-homoserine lactone (3OC12-HSL) as an AHL autoinducer, which binds to the LasI/LasR cognate receptor and modulates the expression of multiple genes responsible for the production of various virulence factors and biofilm formation. Therefore, LasR antagonists could be utilised as novel antivirulence agents for the treatment of infections caused by multi-drug-resistant bacteria such as *P. aeruginosa* [14,15].

**Figure 1:**
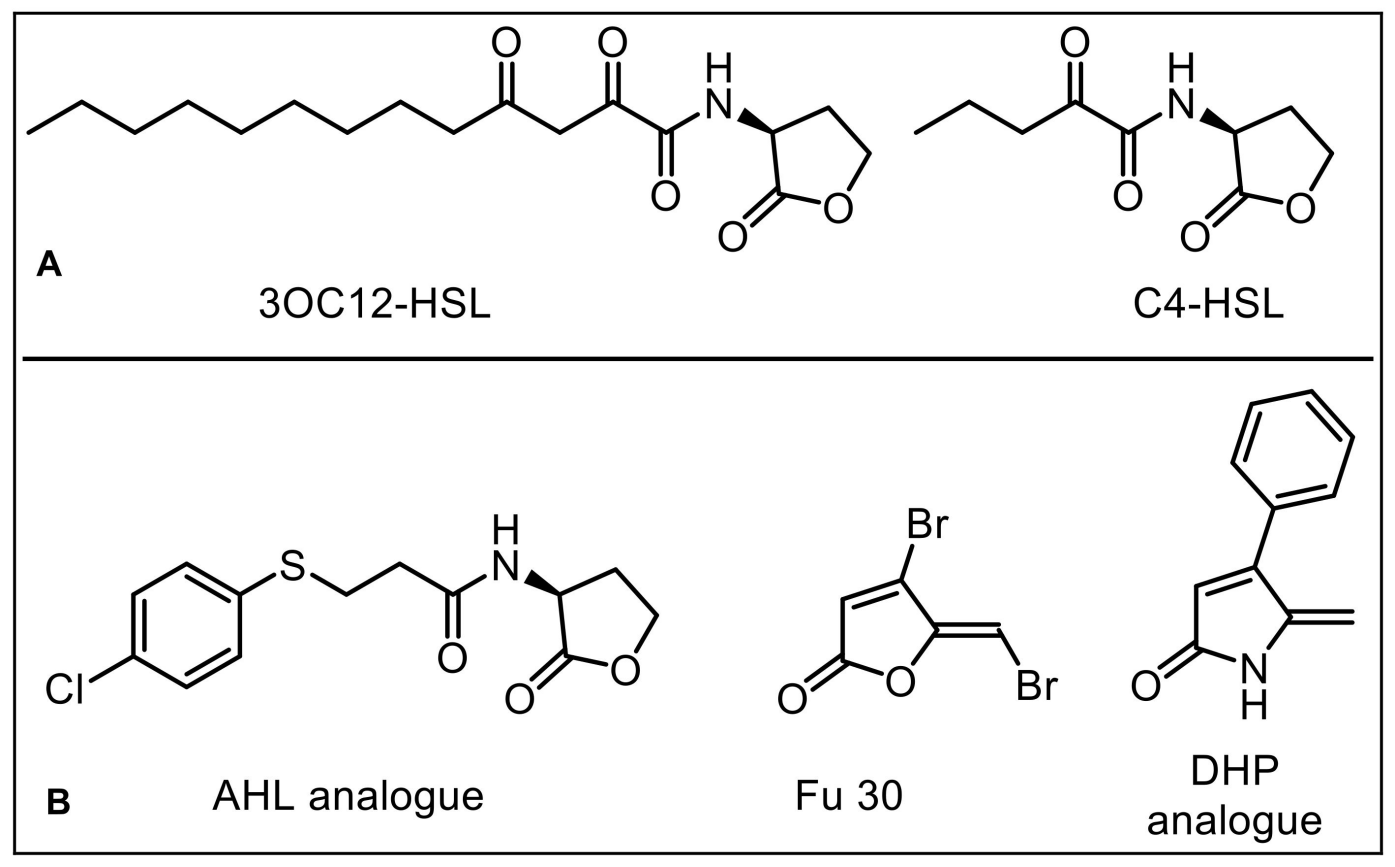
(**A**) AHL-based natural autoinducers of *P. aeruginosa*. (**B**) Synthetic AHL-based quorum-sensing inhibitors (QSIs).

Several natural and synthetic lactones, such as fimbrolides and halogenated furanones, have been reported as AHL mimics and QS inhibitors (Figure 1) [16,17,18]. These inhibitors can inhibit QS and biofilm formation. Structure–function analyses of previously reported furanone and dihydropyrrol-2-one (DHP)-based QSI inhibitors have revealed that the presence of a conjugated exocyclic double bond is critical for their inhibitory activity [19]. We have previously reported DHP-based AHL mimics that contain a five-membered lactam ring and an aryl (phenyl) group at the 4-position and an exocyclic double bond [20]. The stability of lactams from hydrolysis under normal physiological conditions makes them a better choice than the corresponding oxygen-containing (lactone) analogues. The DHP moiety is present in several classes of biologically important natural and synthetic molecules. In our recent work, we synthesised a series of thioether-containing DHP analogues as PqsR antagonists, and several of those analogues exhibited good *pqs* quorum-sensing inhibitory (QSI) and biofilm inhibition activities against POA1 *P. aeruginosa* without affecting bacterial growth [21].

Organoselenium compounds have attracted considerable attention as biologically active compounds due to the low toxicity and antioxidant properties of selenium [22,23,24]. As an antioxidant, selenium acts as an essential element of several redox enzymes such as the enzyme glutathione peroxidase, required for catalysing several metabolic processes in the body [25]. Several organoselenium compounds have been reported as anticancer [26,27], antibacterial [28,29,30,31], and antiviral agents [32,33].

To elaborate on these earlier findings, the present investigation explored further modifications around the DHP motif via the introduction of selenourea and thiourea moieties attached directly to the C-5 exocyclic alkene of the pyrrol-2-one ring. The synthesised compounds were tested against the LasR QS reporter strain of *P. aeruginosa* (MH602). In addition, selenium-containing compounds were also tested for growth inhibition in the Gram-positive *S. aureus* (SA38) and the Gram-negative *E. coli* (K12). The screening results revealed that the selenium compounds were more active compared to the corresponding sulphur analogues.

## 2. Results

### 2.1. Chemistry

**Scheme 1:**
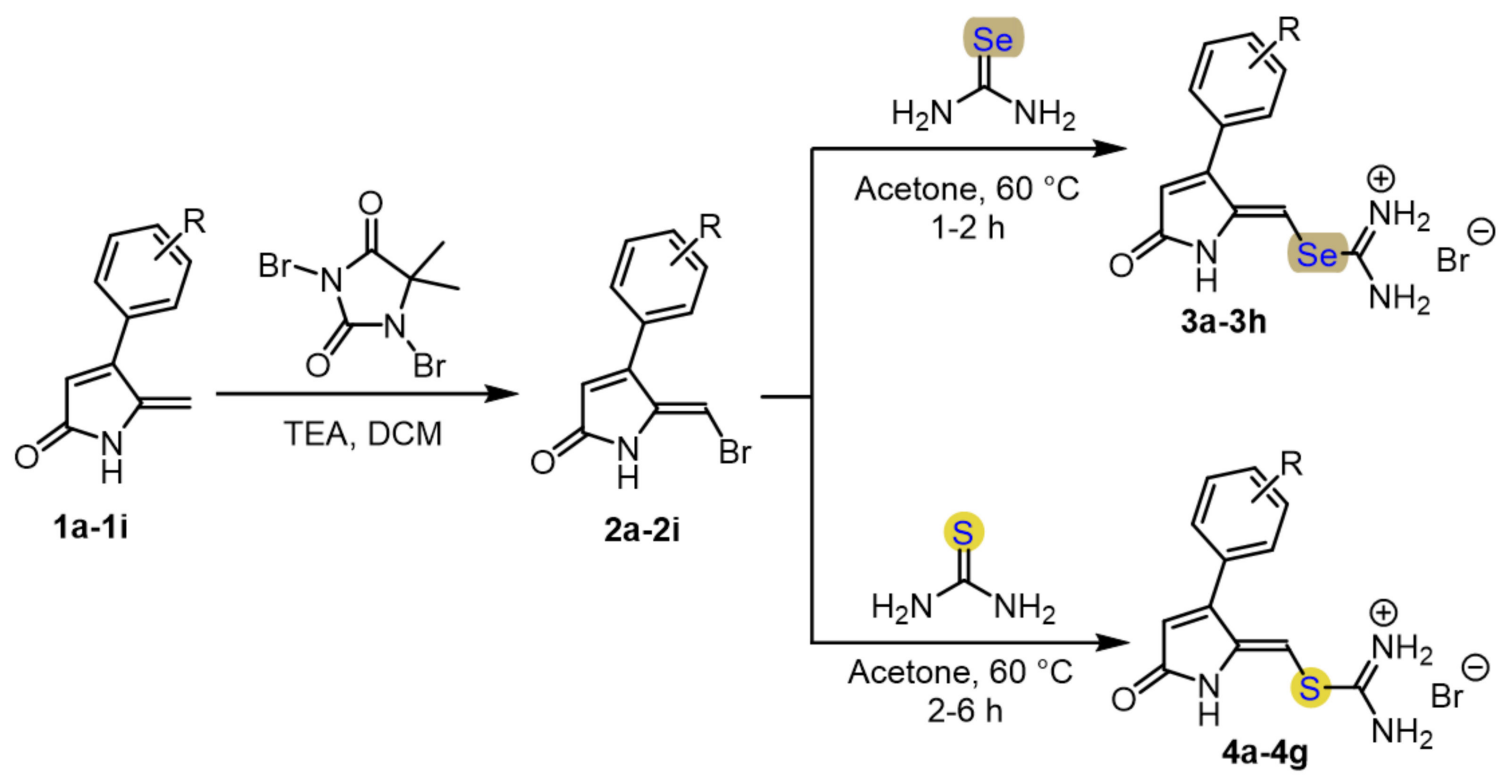
General scheme for the synthesis of seleno-/thio-urea-containing dihydropyrrol-2-one derivatives.

Synthesis of novel selenourea- and thiourea-containing DHP derivatives started with our recently reported key bromo DHP intermediates (**2a–2i**) [21]. In that previous work, it was reported that the bromo DHP compounds undergo an exchange reaction with different thiols and give thioether-containing DHP compounds under mild reaction conditions. In order to further explore the reactivity of the bromo DHP compounds, it was found that heating these bromo DHP compounds with selenourea at 60 °C in acetone resulted in the corresponding DHP carbamimidoselenoate hydrobromide (**3a–3h**) precipitating from the reaction mixture within 1–2 h. The solid compounds could be easily isolated by simple filtration and subsequent washing with diethyl ether to give selenourea-containing analogues in 63–85% yields. Similarly, the corresponding DHP carbamimidothioate hyrobromide analogues (**4a–4g**) were synthesised in 48–96% yields by heating bromo DHP intermediates (**2a–2i**) with thiourea. However, in the case of thiourea, the reactions took longer (approximately 2–6 h) to complete (Scheme 1).

The exchange reaction takes place readily with several different bromo DHP intermediates (**2a–2i**) and no significant effect of substitution on the aryl group of DHP was observed (Table 1).

All the synthesised compounds were characterised by ^1^H NMR, ^13^C NMR, HRMS, and IR spectroscopy. To confirm the presence of a selenium atom in the structure, ^77^Se NMR was carried out for compound **3e** in DMSO-*d*_6_, and this resulted in a single peak at 388.5 ppm, similar to the reported ^77^Se NMR of other Se-containing compounds [34], which validates the presence of one selenium atom in the molecule (Supplementary Materials, File S12).

### 2.2. Quorum-Sensing Inhibition Assay

The novel selenourea and thiourea dihydropyrrol-2-ones were evaluated for their quorum-sensing inhibitory (QSI) activity using the *P. aeruginosa* MH602 P_las_B:gfp reporter strain, which harbours a chromosomal fusion of the lasB promoter to an unstable green fluorescent protein (GFP-ASV) reporter gene and responds to the AHL 3-oxo-dodecanoyl homoserine lactone (3oxo-C12-HSL) [35].

The GFP fluorescence level is a measure for the expression of AHL-mediated QS; therefore, the inhibition of the QS system by synthetic small molecules leads to the decrease in the fluorescence level of GFP, which can be directly correlated with the activity of those inhibitors [36].

The QSI activity of each compound was tested with three different micromolar concentrations (125, 63, and 31 µM) by incubating with bacterial cultures at 37 °C for 15 h. The reduction in GFP fluorescence at λ = 535 nm was determined and expressed as a percentage of inhibition (QSI) compared to the negative control (only bacteria). A reported QS inhibitor Furanone 30 (Fu 30) was employed as a positive control.

**Figure 2:**
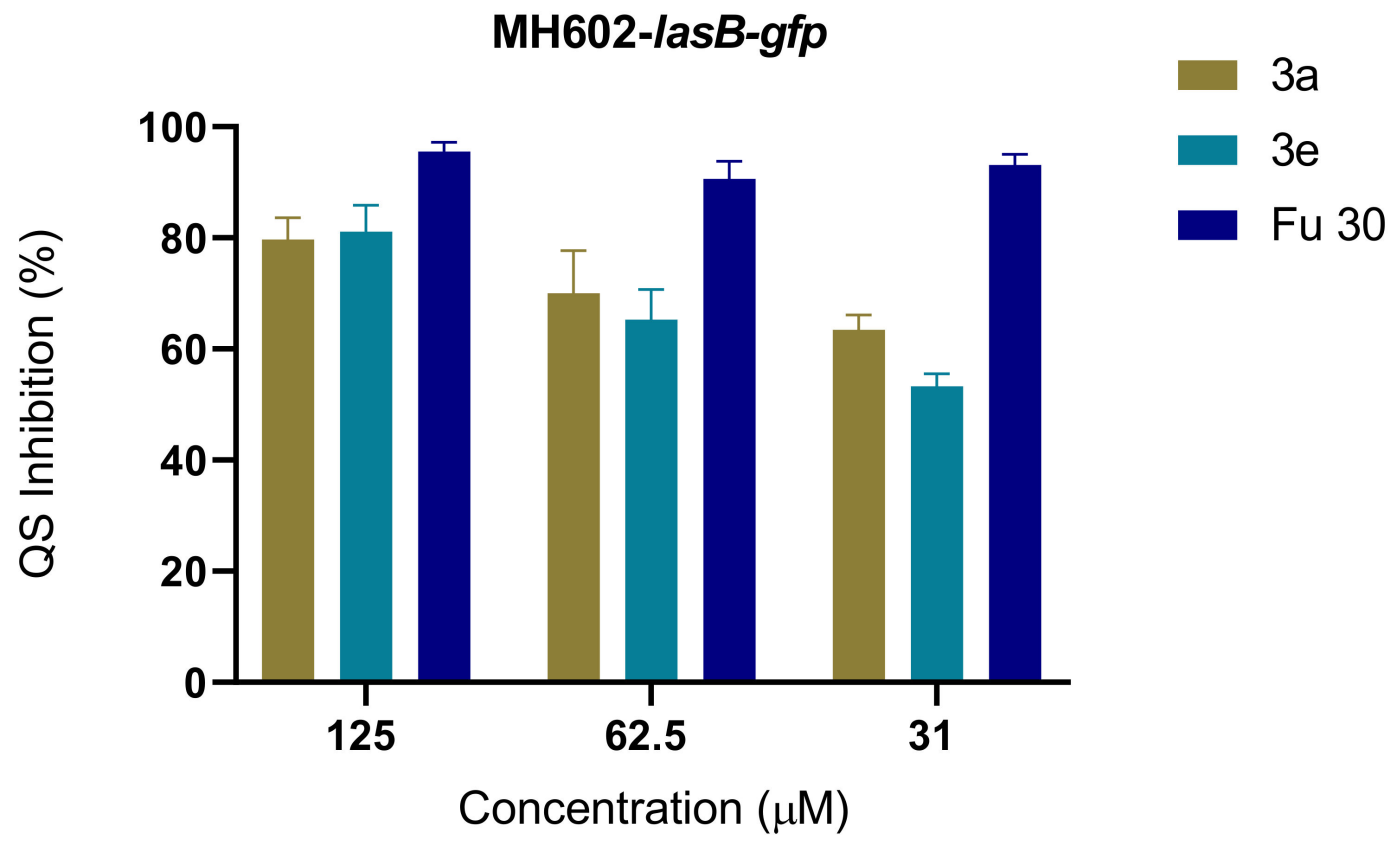
Comparison of % LasR QS inhibition at different concentrations of inhibitors **3a**, **3e**, and reference **Fu 30**.

The QSI activities of all the novel compounds were promising and concentration dependent, as shown in Table 2. Overall, the selenium-containing compounds (**3a–h**) displayed higher QSI activities than the corresponding sulphur analogues (**4a–g**). All the selenium-containing compounds showed greater than 60% QS inhibition at a 125 µM concentration. The QSI activity of selenium compound **3a**, which contained an unsubstituted phenyl group at the C-4 position, produced 80% inhibition at 125 µM and 63% inhibition at 31 µM. The most potent compound was **3e** with 2-Cl substitution at the phenyl ring, producing 81% inhibition at 125 µM and 53% inhibition at 31 µM (Figure 2). The 4-Cl phenyl analogue **3c** produced slightly lower activity of 74% at 125 µM and 42% at 31 µM. The substitution at the C-3 position of lactam with a methyl group led to a decrease in activity as compound **3f** showed only 69% inhibition at 125 µM. The selenium compound **3h** (4-methoxy phenyl) exhibited QS inhibition of 80% at 125 µM and 53% at 31 µM, and this suggests that there is no or a very small impact of different substitutions of the C-4 aryl group on the overall QS inhibition activity of these compounds.

On the other hand, sulphur-containing compounds produced lower QS inhibition. The compound **4a** (C-4 phenyl) showed 50% and 37% QS inhibition at 125 µM and 31 µM, respectively. However, in this series, the most potent analogue was **4d** (4-Br phenyl) which exhibited QS inhibition of 74% at 125 µM and 41% at 31 µM.

### 2.3. Antibacterial Activity

As these new compounds contain cationic selenourea and thiourea functionalities, we were interested to see if these compounds had antibacterial activity. When the compounds were screened for their minimum inhibitory concentrations (MICs), using the Gram-negative bacteria *E. coli* (Table 3), or their effect on the growth of *P. aeruginosa* (Supplementary Materials, File S48), none were able to reduce the growth by 90%, the minimum growth inhibition to obtain a MIC. Selenium-containing compounds produced a high level (23–50%) of growth inhibition of *P. aeruginosa* at 125 µM, while at 31 µM, the growth inhibition induced by the selenium-containing compounds was <20%, except for compound **3c**, which still gave a growth inhibition of 23%. This suggests that these selenium-containing compounds (except **3c**) work predominantly as QS inhibitors at low concentrations.

Nevertheless, when the compounds were screened for their minimum inhibitory concentration (MIC) against the Gram-positive *S. aureus* SA38 (isolated from a human corneal ulcer) (Table 3), the selenium-containing compounds (3a–h) were able to reduce the growth of *S. aureus*. Compounds **3c** and **3e,** which had 4-Cl and 2-Cl substitution, respectively, were the most potent with MICs of 15.6 µM. These did not show haemolysis at 100 µM. The corresponding 4-F and 4-Br derivatives **3b** and **3d** were less active, with MICs of 125 µM and ˃250 µM, respectively. The substitution of a methyl group at the 3-position of the lactam also led to significant loss of antimicrobial activity as compound **3f** had a MIC of 62.5 µM compared to MIC of 15.6 µM for **3c**. On the other hand, none of the corresponding sulphur analogues showed any activity at the maximum concentration of 250 µM. These results further confirm that selenium is critical for antibacterial activity.

## 3. Conclusions

In summary, a new series of selenourea and thiourea dihydropyrrol-2-one compounds were synthesised and screened for LasR QSI activity using the *P. aeruginosa* MH602-P*_las_*B-*gfp* reporter strain. The results showed that most of the compounds produced a concentration-dependent inhibition of *las* QS. Interestingly, selenium-containing compounds were significantly more effective inhibitors compared to the corresponding sulphur-containing analogues, most likely due to the presence of both selenium (and possibly also the cationic +NH_2_). Except for compounds **3d** and **3h**, all the selenium analogues reduced the growth of *S. aureus* SA38, with derivatives **3c** and **3e** showing potent activity with MICs of 15.6 µM. The results indicated that the presence of selenium is essential for both QSI against Gram-negative *P. aeruginosa* and antibacterial activity against Gram-positive *S. aureus*. To the best of our knowledge, this is the first description of dual-acting organoselenium compounds as quorum-sensing inhibitors in Gram-negative (*P. aeruginosa*) and bactericidal activity in Gram-positive bacteria (*S. aureus*). In the future, our efforts will focus on developing more potent selenium-based antibacterial compounds by structure–activity relationships, and we will also evaluate the effect of these compounds on the formation of bacterial biofilm and other virulence factors.

## 4. Materials and Methods

All the reagents and solvents were purchased from commercial sources (Combi-Blocks, Sigma-Aldrich, and Oakwood chemicals) and used without further purification. Reactions were performed using oven-dried glassware. Room temperature (rt) refers to the ambient temperature (25 °C). Progress of reactions was monitored by thin-layer chromatography (TLC) using precoated Merck silica gel 60 F254 plates and visualised using UV light (254 nm). Melting points of the new compounds were determined by an SRS MPA100 OptiMelt instrument and are reported without correction. IR spectra were recorded using a Cary 630 ATR FTIR spectrophotometer. High-resolution mass spectrometry (HRMS) was performed by electrospray (ESI) ionisation using a Thermo LTQ Orbitrap XL instrument at Bioanalytical Mass Spectrometry Facility (BMSF) of Mark Wainwright Analytical Centre, UNSW Sydney. ^1^H and ^13^C-NMR were recorded in deuterated DMSO-*d*6) using Bruker Avance III 300 and Bruker Avance III 400 MHz instruments (Bruker Pty Ltd, Preston, Australia) at 24 °C. Chemical shifts (δ) are reported as relative to the corresponding solvent peak, with tetramethylsilane as the internal standard and quoted in parts per million (ppm). The coupling constants (*J*) are reported in hertz (Hz).

### 4.1. General Procedure for Synthesis of Selenourea and Thiourea DHP Compounds

The corresponding DHP bromide (0.2 mmol) was added to a stirring mixture of selenourea or thiourea (0.22 mmol) in acetone (5 mL) at rt. The mixture was then heated to 60 °C for 1–6 h. Progress of the reaction was monitored by TLC, and after completion of the reaction, the solid precipitate was collected by filtration or by evaporation under vacuum and was further purified by washing with diethyl ether (10 mL).

(*Z*)-(*5*-*oxo*-*3*-*phenyl*-*1*,*5*-*dihydro*-*2H*-*pyrrol*-*2*-*ylidene*) *methyl carbamimidoselenoate hydrobromide* (**3a**): Following the general procedure A, the title product was obtained as a yellow solid (62 mg, 83% yield); mp 146–149 °C; ^1^H NMR (400 MHz, DMSO-*d6*) δ 10.51 (s, 1H), 9.24 (s, 4H), 7.81–7.55 (m, 2H), 7.55–7.24 (m, 3H), 6.47 (d, *J* = 1.6 Hz, 1H), 6.28 (s, 1H). ^13^C NMR (101 MHz, DMSO-*d6*) δ 170.40, 165.78, 150.14, 145.72, 131.06, 129.68, 128.86, 122.46, 94.19. IR (ATR): *ν*_max_ 3155–2985, 1710, 1638; ESI-HRMS *m*/*z*: calcd for C_12_H_11_N_3_OSe [M+H]^+^: 294.0140; found 294.0134.

(*Z*)-(*3*-(*4*-*fluorophenyl*)-*5*-*oxo*-*1*,*5*-*dihydro*-*2H*-*pyrrol*-*2*-*ylidene*)*methyl carbamimidoselenoate hydrobromide* (**3b**): The title product was obtained as a yellow solid (64 mg, 82% yield); mp 162–165 °C; ^1^H NMR (400 MHz, DMSO-*d6*) δ 10.52 (d, *J* = 1.8 Hz, 1H), 9.25 (s, 5H), 7.65 (dd, *J* = 8.7, 5.5 Hz, 2H), 7.34 (t, *J* = 8.9 Hz, 2H), 6.47 (d, *J* = 1.5 Hz, 1H), 6.25 (s, 1H). ^13^C NMR (101 MHz, DMSO-*d6*) δ 170.32, 165.81, 164.18, 161.73, 149.04, 145.81, 131.25, 131.16, 127.52, 127.49, 122.61, 115.95, 115.74, 94.11. IR (ATR): *ν*_max_ 3243, 3100, 1681, 1643; ESI-HRMS *m/z*: calcd for C_12_H_11_FN_3_OSe [M+H]^+^: 312.0046; found 312.0042.

(*Z*)-(*3*-(*4*-*chlorophenyl*)-*5*-*oxo*-*1*,*5*-*dihydro*-*2H*-*pyrrol*-*2*-*ylidene*)*methyl carbamimidoselenoate hydrobromide* (**3c**): The title product was obtained as a yellow solid (71 mg, 85% yield); mp 170–175 °C; ^1^H NMR (400 MHz, DMSO-*d6*) δ 10.56 (s, 1H), 9.23 (s, 5H), 7.68–7.54 (m, 4H), 6.52 (d, *J* = 1.6 Hz, 1H), 6.28 (s, 1H). ^13^C NMR (101 MHz, DMSO-*d6*) δ 170.24, 165.73, 148.83, 145.50, 134.54, 130.72, 129.88, 128.87, 122.95, 94.27. IR (ATR): *ν*_max_ 3170–2993, 1684, 1640; ESI-HRMS *m/z*: calcd for C_12_H_11_ClN_3_OSe [M+H]^+^: 327.9750; found 327.9743.

(*Z*)-(*3*-(*4*-*bromophenyl*)-*5*-*oxo*-*1*,*5*-*dihydro*-*2H*-*pyrrol*-*2*-*ylidene*)*methyl carbamimidoselenoate hydrobromide* (**3d**): The title product was obtained as a yellow solid (66 mg, 70% yield); mp 173–175 °C; ^1^H NMR (400 MHz, DMSO-*d6*) δ 10.56 (s, 1H), 9.23 (s, 4H), 7.71 (d, *J* = 8.5 Hz, 2H), 7.55 (d, *J* = 8.5 Hz, 2H), 6.53 (d, *J* = 1.6 Hz, 1H), 6.29 (s, 1H). ^13^C NMR (101 MHz, DMSO-*d6*) δ 170.19, 165.68, 148.86, 145.35, 131.76, 130.92, 130.21, 123.24, 122.89, 94.27. IR (ATR): *ν*_max_ 3165–2986, 1702, 1649; ESI-HRMS *m/z*: calcd for C_12_H_11_BrN_3_OSe [M+H]^+^: 371.9245; found 371.9237.

(*Z*)-(*3*-(*2*-*chlorophenyl*)-*5*-*oxo*-*1*,*5*-*dihydro*-*2H*-*pyrrol*-*2*-*ylidene*)*methyl carbamimidoselenoate hydrobromide* (**3e**): The title product was obtained as a yellow solid (69 mg, 84% yield); mp 165–168 °C; ^1^H NMR (400 MHz, DMSO-*d6*) δ 10.65 (s, 1H), 9.23 (s, 4H), 7.63 (dd, *J* = 7.8, 1.4 Hz, 1H), 7.60–7.41 (m, 3H), 6.49 (d, *J* = 1.5 Hz, 1H), 5.99 (s, 1H).^13^C NMR (101 MHz, DMSO-*d6*) δ 170.73, 165.84, 147.30, 146.02, 132.69, 132.10, 131.41, 130.43, 130.09, 127.63, 125.74, 94.75. ^77^Se NMR (76 MHz, DMSO-*d6*) δ 388.56. IR (ATR): *ν*_max_ 3156–3031, 1689, 1645; ESI-HRMS *m/z*: calcd for C_12_H_10_ClN_3_OSe [M+H]^+^: 327.9750; found 327.9750.

(*Z*)-(*3*-(*4*-*chlorophenyl*)-*4*-*methyl*-*5*-*oxo*-*1*,*5*-*dihydro*-*2H*-*pyrrol*-*2*-*ylidene*)*methyl carbamimidoselenoate hydrobromide* (**3f**): The title product was obtained as a light yellow solid (54 mg, 63% yield); mp 180–182 °C; ^1^H NMR (400 MHz, DMSO-*d6*) δ 10.58 (s, 1H), 9.12 (d, *J* = 23.4 Hz, 4H), 7.58 (d, *J* = 8.5 Hz, 2H), 7.50 (d, *J* = 8.5 Hz, 2H), 5.92 (s, 1H), 1.85 (s, 3H). ^13^C NMR (101 MHz, DMSO-*d6*) δ 171.51, 166.66, 147.65, 142.19, 134.20, 131.88, 131.82, 129.89, 129.18, 91.15, 9.64. IR (ATR): *ν*_max_ 3258–2976, 1698, 1640; ESI-HRMS *m/z*: calcd for C_13_H_12_FNOS [M+H]^+^: 341.9907; found 341.9905.

(*Z*)-(*3*-(*2*-*fluorophenyl*)-*5*-*oxo*-*1*,*5*-*dihydro*-*2H*-*pyrrol*-*2*-*ylidene*)*methyl carbamimidoselenoate hydrobromide* (**3g**): The title product was obtained as a yellow solid (64 mg, 82% yield); mp 159–161 °C; ^1^H NMR (300 MHz, DMSO-*d6*) δ 10.63 (s, 1H), 9.23 (s, 4H), 7.62-7.51 (m, 2H), 7.38–7.30 (m, 2H), 6.50 (s, 1H), 6.17 (s, 1H). ^13^C NMR (76 MHz, DMSO-*d6*) δ 170.28, 165.55, 160.45, 154.88, 145.32, 143.41, 131.78, 131.66, 131.47, 125.13, 124.69, 118.69, 116.40, 116.11, 94.39. IR (ATR): *ν*_max_ 3162–3013, 1690, 1647; ESI-HRMS *m/z*: calcd for C_12_H_11_FN_3_OSe [M+H]^+^: 312.0046; found 312.0040.

(*Z*)-(*3*-(*4*-*methoxyphenyl*)-*5*-*oxo*-*1*,*5*-*dihydro*-*2H*-*pyrrol*-*2*-*ylidene*)*methyl carbamimidoselenoate hydrobromide* (**3h**): The title product was obtained as a yellow solid (65 mg, 80% yield); mp 138–140 °C; ^1^H NMR (600 MHz, DMSO-*d6*) δ 10.41 (s, 1H), 9.22 (s, 4H), 7.55 (d, *J* = 8.6 Hz, 2H), 7.08–7.02 (m, 2H), 6.38 (d, *J* = 1.6 Hz, 1H), 6.26 (s, 1H), 3.81 (s, 3H). ^13^C NMR (151 MHz, DMSO-*d6*) δ 170.54, 165.83, 160.47, 149.88, 146.03, 130.33, 123.35, 121.10, 114.31, 93.67, 55.38. IR (ATR): *ν*_max_ 3102–2992, 1681, 1648; ESI-HRMS *m/z*: calcd for C_13_H_13_N_3_O_2_Se [M+H]^+^: 324.0246; found 324.0241.

(*Z*)-(*5*-*oxo*-*3*-*phenyl*-*1*,*5*-*dihydro*-*2H*-*pyrrol*-*2*-*ylidene*)*methyl carbamimidothioate hyrobromide* (**4a**): The title product was obtained as a yellow solid (45 mg, 69% yield); mp 172–175 °C; ^1^H NMR (400 MHz, DMSO-*d6*) δ 10.75 (s, 1H), 9.14 (d, *J* = 23.6 Hz, 4H), 7.65–7.56 (m, 2H), 7.55–7.46 (m, 3H), 6.51 (d, *J* = 1.5 Hz, 1H), 5.96 (s, 1H). ^13^C NMR (101 MHz, DMSO-*d6*) δ 170.33, 167.87, 150.28, 147.24, 130.87, 129.75, 128.86, 122.96, 93.01. IR (ATR): *ν*_max_ 3251–3055, 1684, 1650; ESI-HRMS *m/z*: calcd for C_12_H_11_N_3_OS [M+H]^+^: 246.0696; found 246.0693.

(*Z*)-(*3*-(*4*-*fluorophenyl*)-*5*-*oxo*-*1*,*5*-*dihydro*-*2H*-*pyrrol*-*2*-*ylidene*)*methyl carbamimidothioate hyrobromide* (**4b**) The title product was obtained as a yellow solid (65 mg, 94% yield); mp 175–177 °C; ^1^H NMR (600 MHz, DMSO-*d6*) δ 10.74 (s, 1H), 9.17 (d, *J* = 41.0 Hz, 4H), 7.69–7.63 (m, 2H), 7.37–7.32 (m, 2H), 6.50 (d, *J* = 1.6 Hz, 1H), 5.93 (s, 1H). ^13^C NMR (151 MHz, DMSO-*d6*) δ 170.70, 168.32, 164.26, 162.62, 149.65, 147.77, 131.70, 131.65, 127.76, 127.74, 123.53, 116.38, 116.23, 93.40. IR (ATR): *ν*_max_ 3251–3054, 1690, 1650; ESI-HRMS *m/z*: calcd for C_12_H_10_FN_3_OS [M+H]^+^: 264.0601; found 264.0598.

(*Z*)-(*3*-(*4*-*chlorophenyl*)-*5*-*oxo*-*1*,*5*-*dihydro*-*2H*-*pyrrol*-*2*-*ylidene*)*methyl mcarbamimidothioate hydrobromide* (**4c**): The title product was obtained as a light orange solid (52 mg, 71 % yield); mp 149–151 °C; ^1^H NMR (400 MHz, DMSO-*d6*) δ 10.78 (s, 1H), 9.12 (d, *J* = 21.5 Hz, 4H), 7.67–7.60 (m, 2H), 7.60–7.54 (m, 2H), 6.54 (d, *J* = 1.5 Hz, 1H), 5.95 (s, 1H); ^13^C NMR (101 MHz, DMSO-*d6*) δ 170.16, 167.77, 148.99, 146.98, 134.62, 130.72, 129.69, 128.88, 123.44, 93.12. IR (ATR): *ν*_max_ 3155–2991, 1685, 1643; ESI-HRMS *m/z*: calcd for C_12_H_11_ClN_3_OS [M+H]^+^: 280.0306; found 280.0306.

(*Z*)-(*3*-(*4*-*bromophenyl*)-*5*-*oxo*-*1*,*5*-*dihydro*-*2H*-*pyrrol*-*2*-*ylidene*)*methyl carbamimidothioate hydrobromide* (**4d**): The title product was obtained as a light orange solid (78 mg, 96% yield); mp 169–171 °C; ^1^H NMR (300 MHz, DMSO-*d6*) δ 10.78 (s, 1H), 9.13 (s, 4H), 7.72 (d, *J* = 8.5 Hz, 2H), 7.56 (d, *J* = 8.5 Hz, 2H), 6.56 (d, *J* = 1.5 Hz, 1H), 5.96 (s, 1H). ^13^C NMR (76 MHz, DMSO-*d6*) δ 170.12, 167.72, 149.03, 146.86, 131.77, 130.92, 130.02, 123.39, 123.33, 93.10. IR (ATR): *ν*_max_ 3140–2959, 1696, 1651; ESI-HRMS *m/z*: calcd for C_12_H_11_BrN_3_OS [M+H]^+^: 323.9801; found 323.9802.

(*Z*)-(*3*-(*2*-*fluorophenyl*)-*5*-*oxo*-*1*,*5*-*dihydro*-*2H*-*pyrrol*-*2*-*ylidene*)*methyl carbamimidothioate hydrobromide* (**4e**): The title product was obtained as a yellow solid (45 mg, 66% yield); mp 166–168 °C; ^1^H NMR (400 MHz, DMSO-*d6*) δ 10.85 (s, 1H), 9.14 (s, 4H), 7.65 – 7.50 (m, 2H), 7.43 – 7.29 (m, 2H), 6.53 (s, 1H), 5.84 (s, 1H). ^13^C NMR (101 MHz, DMSO-*d6*) δ 183.86, 170.21, 167.66, 160.47, 158.00, 146.79, 143.50, 131.83, 131.74, 131.44, 125.63, 125.60, 124.69, 124.66, 118.48, 118.34, 116.37, 116.15, 93.22. IR (ATR): *ν*_max_ 3159-3028, 1691, 1650; ESI-HRMS *m/z*: calcd for C_12_H_10_FN_3_OS [M+H]^+^: 264.0601; found 264.0600.

(*Z*)-(*3*-(*2*-*chlorophenyl*)-*5*-*oxo*-*1*,*5*-*dihydro*-*2H*-*pyrrol*-*2*-*ylidene*)*methyl carbamimidothioate hydrobromide* (**4f**): The title product was obtained as a yellow solid (35 mg, 48% yield); mp 187–190 °C; ^1^H NMR (300 MHz, DMSO-*d6*) δ 10.87 (s, 1H), 9.12 (s, 4H), 7.67 – 7.41 (m, 4H), 6.52 (s, 1H), 5.65 (s, 1H). ^13^C NMR (76 MHz, DMSO-*d6*) δ 170.19, 167.52, 146.98, 146.91, 132.23, 131.64, 130.99, 129.97, 129.38, 127.17, 125.83, 93.09. IR (ATR): *ν*_max_ 3225-3040, 1690, 1651; ESI-HRMS *m/z*: calcd for C_12_H_11_ClN_3_OS [M+H]^+^: 280.0306; found 280.0303.

(*Z*)-(*5*-*oxo*-*3*-(*3*-(*trifluoromethyl*)*phenyl*)-*1*,*5*-*dihydro*-*2H*-*pyrrol*-*2*-*ylidene*)*methyl carbamimidothioate hydrobromide* (**4g**): The title product was obtained as a yellow solid (60 mg, 76% yield); mp 168–170 °C;^1^H NMR (400 MHz, DMSO-*d6*) δ 10.84 (s, 1H), 9.13 (d, *J* = 22.8 Hz, 4H), 7.95 (s, 1H), 7.94–7.85 (m, 2H), 7.76 (t, *J* = 7.8 Hz, 2H), 6.67 (d, *J* = 1.5 Hz, 1H), 5.94 (s, 1H).^13^C NMR (151 MHz, DMSO-*d6*) δ 170.04, 167.78, 148.63, 146.93, 132.97, 131.87, 129.96, 129.72, 129.51, 126.24, 125.48, 125.45, 124.85, 124.42, 123.04, 93.27. IR (ATR): *ν*_max_ 3120-3008, 1710, 1641; ESI-HRMS *m/z*: calcd for C_13_H_10_FN_3_OS [M+H]^+^: 314.0569; found 314.0565.

### 4.2. Quorum-Sensing Inhibition Assay

*P. aeruginosa* MH602 P*_las_*B::*gfp*(ASV) reporter strain, which harbours a chromosomal fusion of the *lasB* promoter to an unstable *gfp* gene and responds to the AHL 3-oxo-dodecanoyl homoserine lactone (3oxo-C12-HSL), was used to evaluate the QSI activity of the synthesised compounds on QS signalling. An overnight culture was prepared in Luria-Bertani (LB10) media supplemented with gentamycin (40 μM). This culture was then diluted (1:100) in TSB/LB10 (4:1) medium supplemented with gentamycin (40 μM), and 200 μL of aliquots were dispensed to flat-bottom 96-well plate wells (Costar). The culture was supplemented with varying concentrations of test compounds dissolved in dimethyl sulfoxide, with the final concentrations of test compounds of 125, 63, and 31 μM. Wells with bacterial culture but no compound were used as a negative control, while wells supplemented with the furanone Fu-30 were used as a positive control. Plates were incubated for 15 h at 37 °C with shaking at 150 rpm. After incubation, the reading of the fluorescence of GFP (excitation, 485 nm; emission, 520 nm) and cell growth (OD at 600 nm) was taken in a plate reader (FLUOstar Omega, BMG Labtech, Ortenberg, Germany).

### 4.3. Analysis of the Minimum Inhibitory and Minimum Bactericidal Concentration of Compounds

The antimicrobial activity of compounds was evaluated using the broth microdilution assay described by The Clinical and Laboratory Standards Institute. Briefly, *Staphylococcus aureus* SA38 or *Escherichia coli* K12 were grown to mid-log phase (18–24 h incubation) in Muller–Hinton broth (MHB) with shaking at 120 rpm at 37 °C. Following incubation, bacteria were washed two times using phosphate-buffered saline (pH 7.4) with centrifugation for 10 min after each wash. After washing, bacteria were diluted with fresh MHB to a turbidity of OD600 of 0.1 (which gave 1 × 10^8^ cfu/mL on retrospective plate counts) and then further diluted to achieve 1 × 10^6^ cfu/mL as a final bacterial concentration. Each compound was added at concentrations ranging from 250 to 3.9 µM through serial two-fold dilution. Wells containing only bacteria and without any compound were used as a negative control and only MHB media as blank. Gentamicin was used as a positive control. The microtiter plate was wrapped with paraffin to prevent evaporation and incubated with shaking at 120 rpm and 37 °C for 18–24 h. After incubation, spectrophotometric reading of the wells was taken at 600 nm using a FLUOstar Omega (BMG Labtech, Mornington, Victoria, Australia). The minimum inhibitory concentration (MIC) was defined as the lowest concentration of the compounds that produce >90% inhibition of microbial growth. Each experiment was performed with two independent repeats. The MBC was determined as the lowest concentration of compound that reduced bacterial growth by >99.99% after the enumeration of viable bacteria by plate counts compared to bacteria grown without any compounds.

### 4.4. Lysis of Horse Red Blood Cells

The haemolytic activities of compounds **3c** and **3e** were determined using horse red blood cells (HRBCs; Sigma) as described previously [37]. The HRBCs were washed three times with PBS at 470× *g* for 5 min. Compounds (100 μM, 50 μM and 25 μM, in PBS) were added to the washed HRBCs and incubated at 37 °C for 4 h. After incubation, the cells were pelleted at 1057× *g* for 5 min, and the supernatant was removed to assess the release of haemoglobin by measuring OD540nm. HRBCs in PBS and HRBCs in distilled water were used as negative (diluent) and positive controls to achieve 0% and 100% lysis, respectively. The relative OD of HRBCs treated with compounds **3c** and **3e** were compared to that treated with distilled water were used to determine the relative percentage of haemolysis. Two separate trials were carried out in triplicates.

% haemolysis = (absorbance of test compound) − (absorbance of diluent)/(absorbance of positive control) − (absorbance of diluent) * 100.

## Figures and Tables

**Table 1:** Structures and yields of synthesised seleno-/thio-urea-containing dihydropyrrol-2-one compounds.

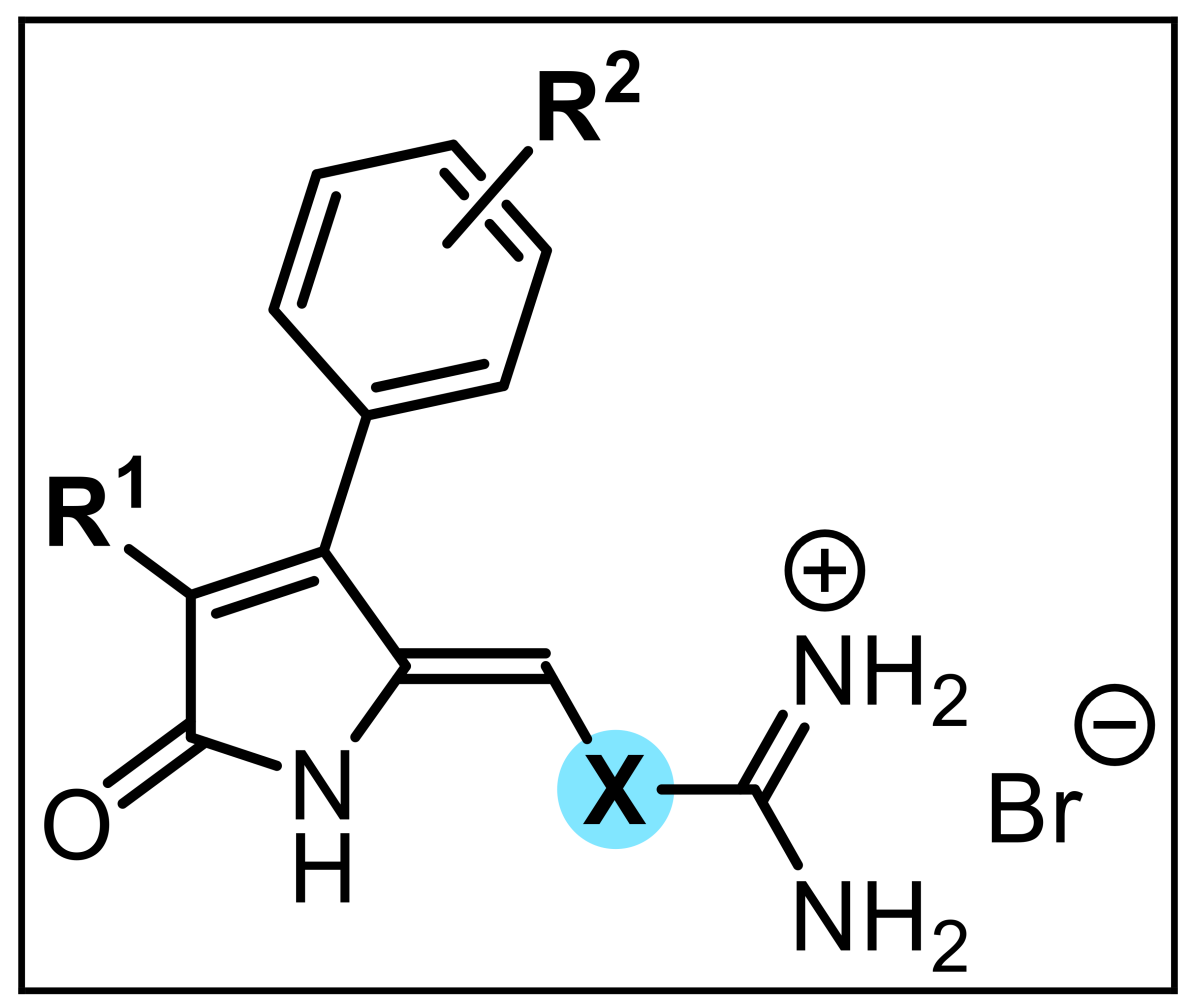				
**Compound**	**R^1^**	**R^2^**	**X**	**Yield (%)**
3a	H	H	Se	83
3b	H	4-F	Se	82
3c	H	4-Cl	Se	85
3d	H	4-Br	Se	70
3e	H	2-Cl	Se	84
3f	CH_3_	4-Cl	Se	63
3g	H	2-F	Se	82
3h	H	4-OCH_3_	Se	80
4a	H	H	S	69
4b	H	4-F	S	94
4c	H	4-Cl	S	71
4d	H	4-Br	S	96
4e	H	2-F	S	66
4f	H	2-Cl	S	48
4g	H	3-CF_3_	S	76

**Table 2:** % LasR QS inhibition at different concentrations of inhibitors **3a–3h**, **4a–4f**, and reference **fu 30**.

Compound	*P. aeruginosa* MH602		
	125 µM	61.5 µM	31 µM
**3a**	**80** ± 4	**70** ± 8	**63** ± 3
3b	67 ± 2	43 ± 6	38 ± 6
3c	74 ± 2	52 ± 3	42 ± 4
3d	76 ± 1	58 ± 6	42 ± 4
**3e**	**81** ± 5	**65** ± 5	**53** ± 2
3f	69 ± 6	49 ± 6	37 ± 3
3g	66 ± 7	49 ± 4	38 ± 4
3h	80 ± 6	64 ± 7	53 ± 4
4a	50 ± 1	44 ± 0	37 ± 1
4b	54 ± 3	38 ± 13	34 ± 5
4c	70 ± 3	48 ± 5	35 ± 3
4d	74 ± 2	54 ± 5	41 ± 5
4e	40 ± 4	29 ± 7	23 ± 9
4f	63 ± 1	55 ± 6	49 ± 11
4g	56 ± 2	42 ± 2	34 ± 3
Fu 30	96 ± 2	91 ± 3	93 ± 2

**Table 3:** MIC determined for compounds against Gram-positive *S. aureus* (SA38) and Gram-negative bacteria (*E. coli* K12). NT-Not tested.

Entry	Compounds	MIC/MBC (µM)	
		*S. aureus* 38	*E. coli* K12
1	3a	31.2	>250
2	3b	125	NT
3	**3c**	**15.6/31.2**	>250
4	3d	>250	NT
5	**3e**	**15.6/62.4**	>250
6	3f	62.5	>250
7	3g	62.5	>250
8	3h	>250	NT
9	4a	>250	NT
10	4b	>250	NT
11	4c	>250	NT
12	4d	>250	NT
13	4e	>250	NT
14	4g	>250	NT
15	4f	>250	NT
16	Gentamicin	<0.4	NT

## Data Availability

Data is contained within the article and Supplementary Materials.

## Supplementary Materials

The following are available online at https://www.mdpi.com/2079-6382/10/3/321/s1, Figures S2–S32: ^1^H and ^13^C NMR spectra of the compounds; Figure S12: ^77^Se NMR spectrum of compound 3e; Figures S33–S47: HRMS data of the compounds; Figures S48–S52: IR Spectra of compounds; Figure S53: Growth inhibition data (*P. aeruginosa* MH602).
