# Supplementary material for: Novel Seleno- and Thio-Urea Containing Dihydropyrrol-2-One Analogues as Antibacterial Agents
Source: Antibiotics (Basel). 2021 Mar 19;10(3):321. doi: 10.3390/antibiotics10030321 (PMC8003518; doi:10.3390/antibiotics10030321)
Supplement: Supplementary file 1 [file antibiotics-10-00321-s001.pdf]

Supporting information

Novel Seleno- and Thio-urea Containing Dihydropyrrol-2-one Analogues as Antibacterial Agents

Shekh Sabir [1], Tsz Tin Yu,[1] Rajesh Kuppusamy,[1] Basmah Almohaywi,[2] George Iskander,[1] Theerthankar Das,[3] Mark Willcox,[4] David StC. Black,[1] and Naresh Kumar [1*]

[1] School of Chemistry, The University of New South Wales, NSW 2052 Sydney, Australia; s.sabir@student.unsw.edu.au (S.S.); tsztin.yu@unsw.edu.au (T. T.Y.); r.kuppusamy@ad.unsw.edu.au (R.K.); g.iskanderb@gmail.com (G. I.); d.black@unsw.edu.au (D.S.B.)

[2] School of Pharmacy, King Khalid University Abha 6142, Saudi Arabia, bal-mohawe@kku.edu.sa (B.A.)

[3] Department of Infectious Diseases and Immunology, School of Medical Sciences, The University of Sydney, NSW 2006 Sydney, Australia; das.ashishkumar@sydney.edu.au (T.D.)

[4] School of Optometry and Vision Science, The University of New South Wales, NSW 2052 Sydney, m.willcox@unsw.edu.au (M.W.)

* Correspondence: n.kumar@unsw.edu.au; Tel.: +61-29385-4698; Fax: +61-29385-6141

Contents

^{1}H and ^{13}C NMR spectra of the compounds S2-S32

^{77}Se NMR spectrum of compound **3e** …………………………………………………………S12

HRMS data of the compounds………………………………………………………………S33-S47

IR Spectra of compounds…………………………………………………………………..S48-S52

Growth inhibition data (*P.aeruginosa* MH602)...….. …S53

^{1}H NMR spectrum of compound **3a**

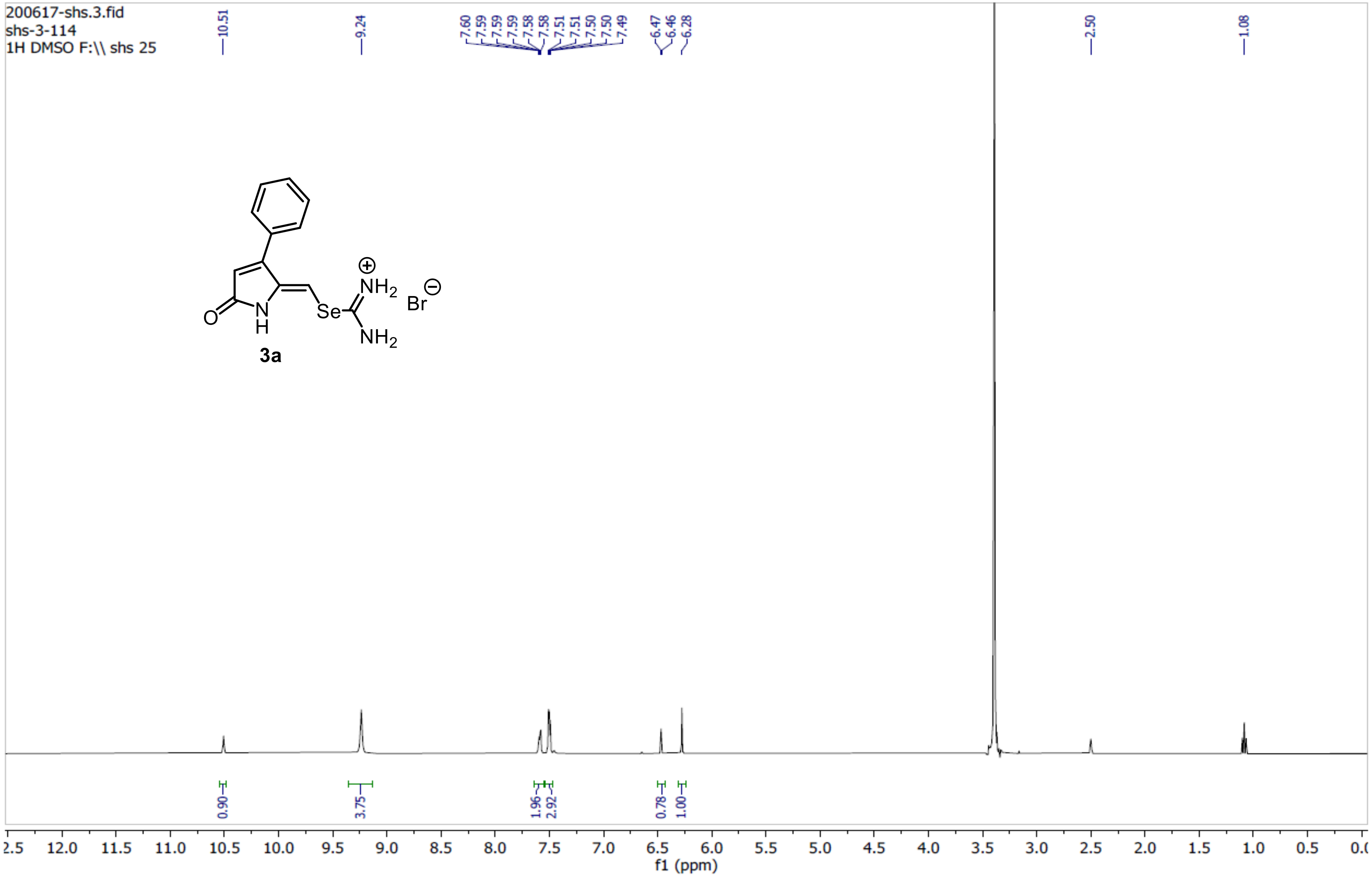

^{13}C NMR spectrum of compound **3a**

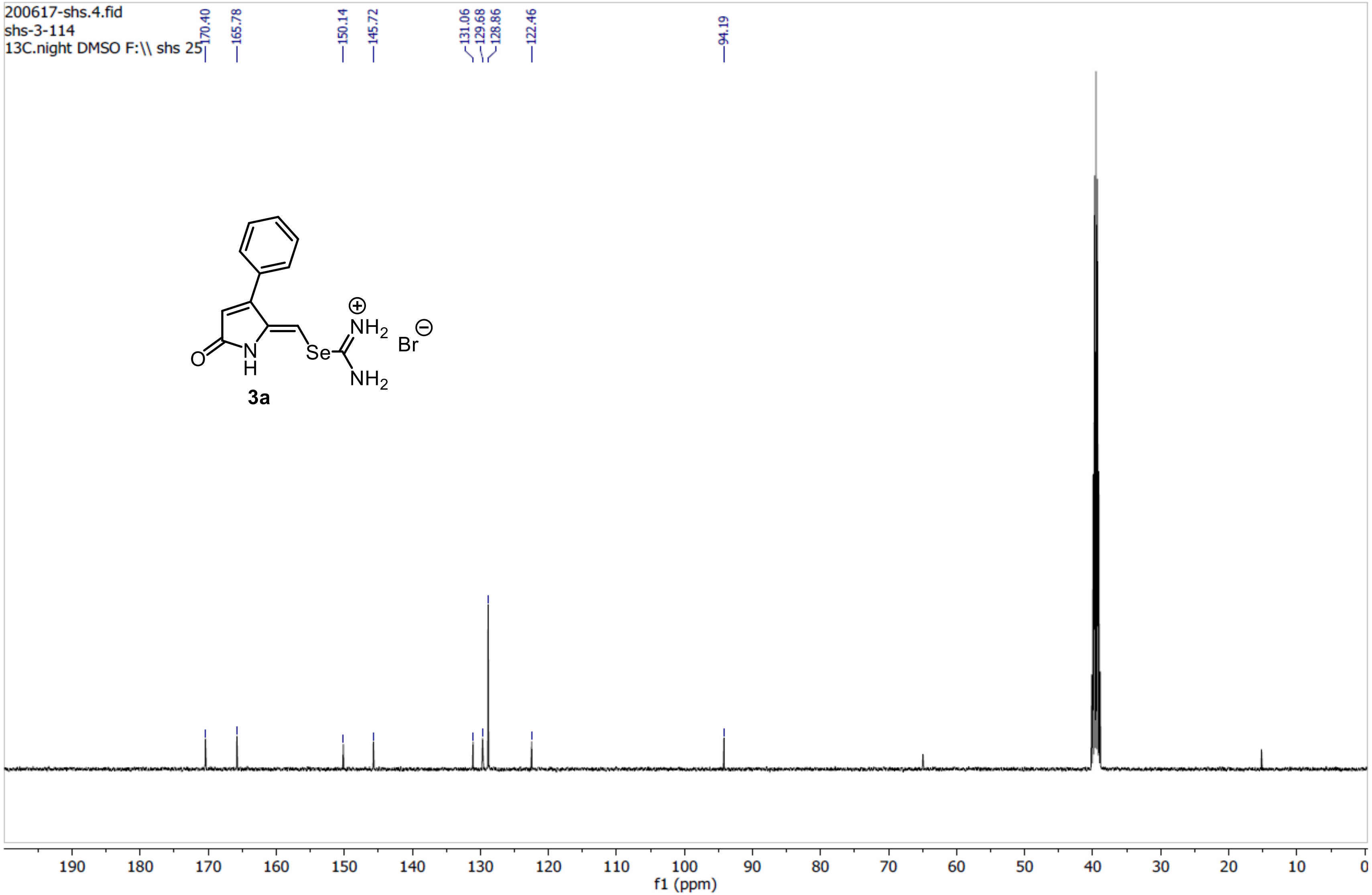

^{1}H NMR spectrum of compound **3b**

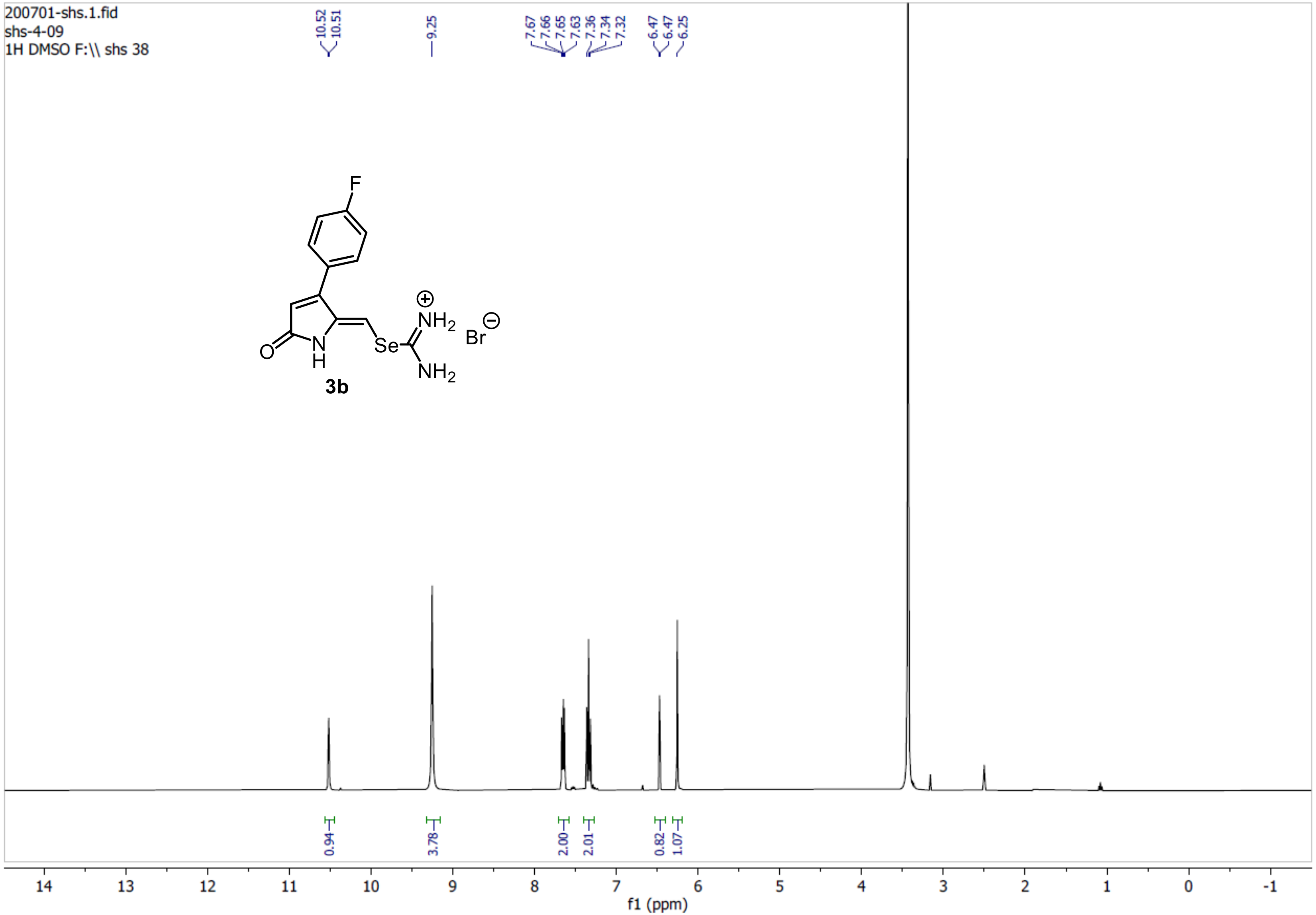

^{13}C NMR spectrum of compound **3b**

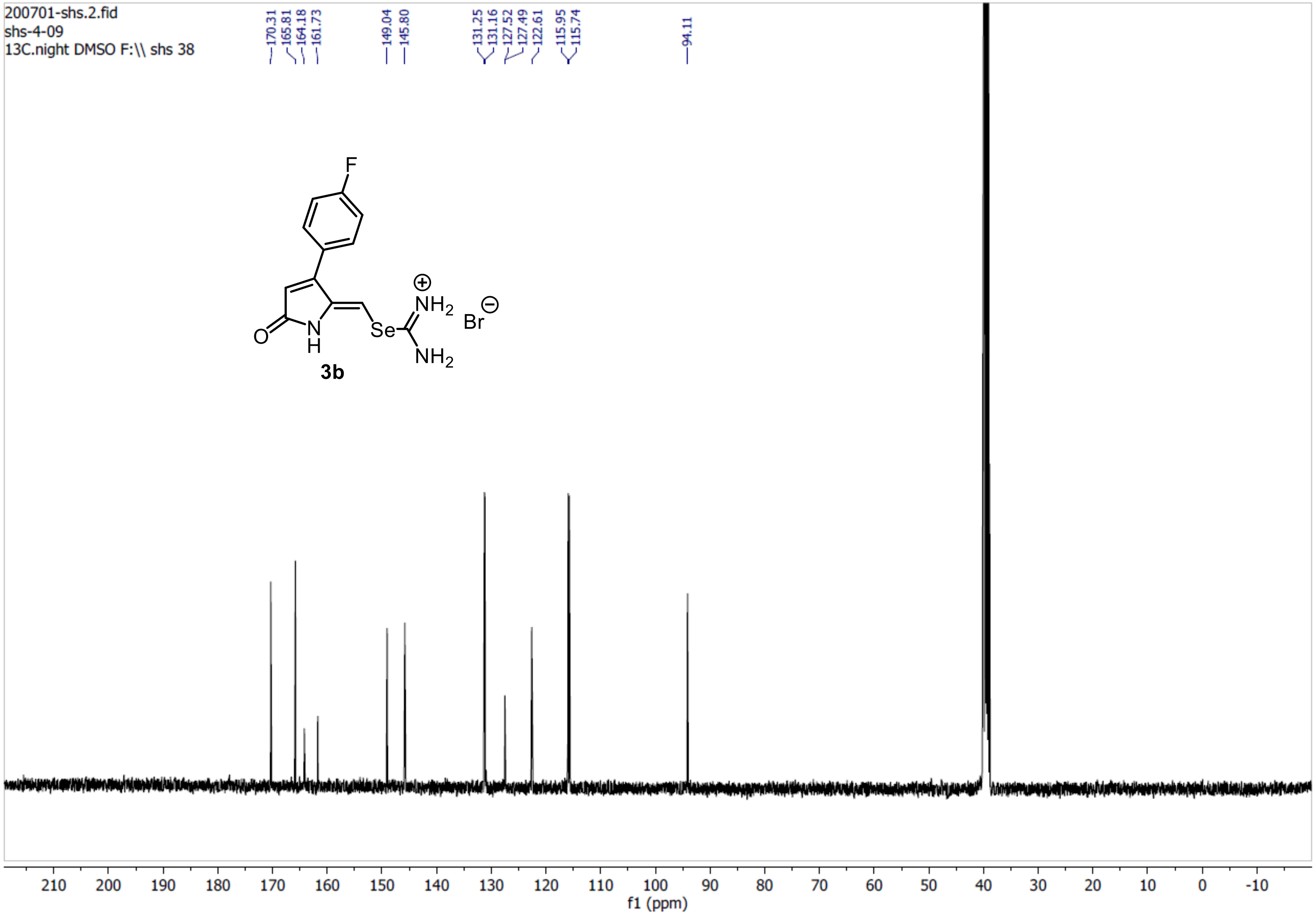

^{1}H NMR spectrum of compound **3c**

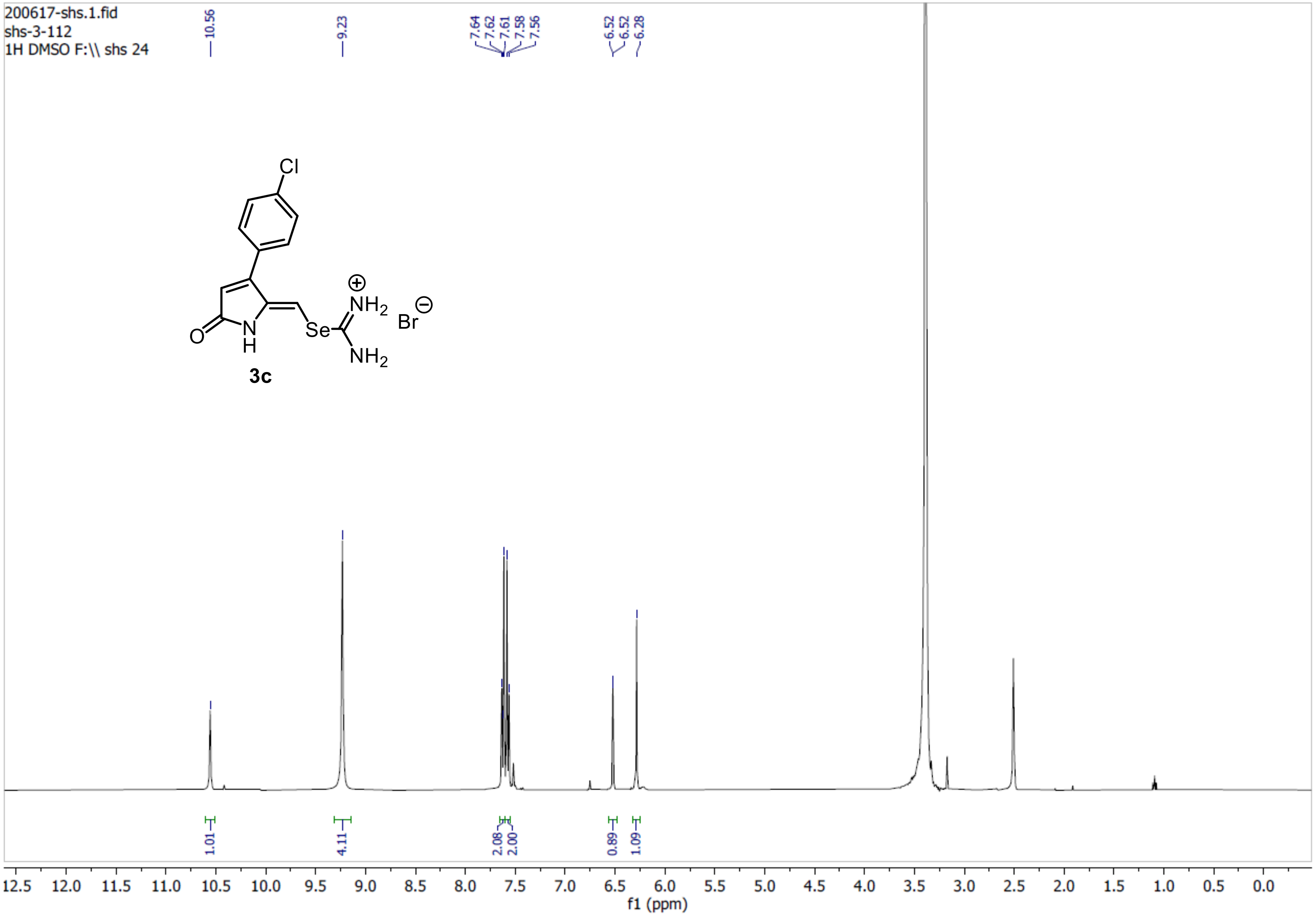

^{13}C NMR spectrum of compound **3c**

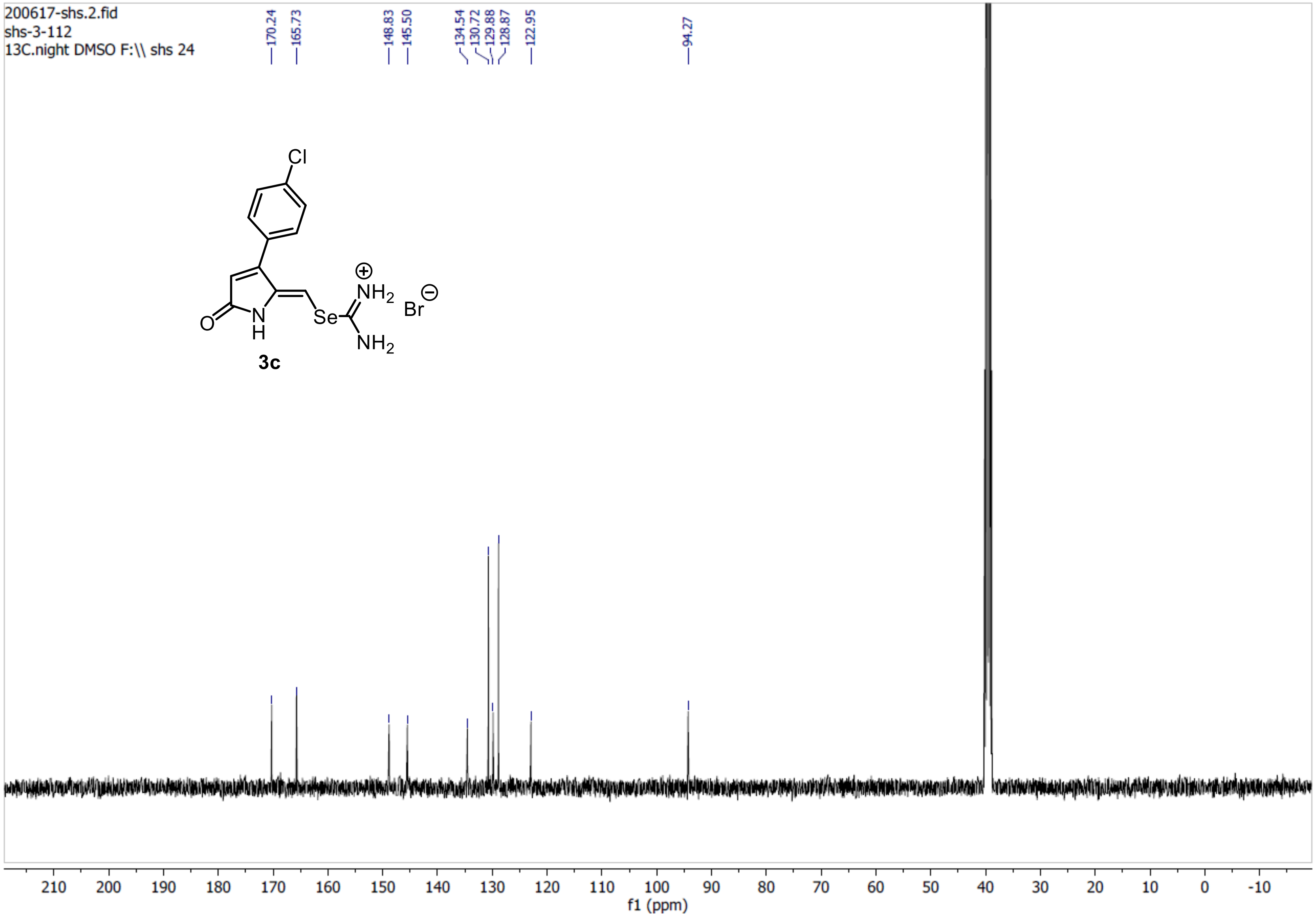

^{1}H NMR spectrum of compound **3d**

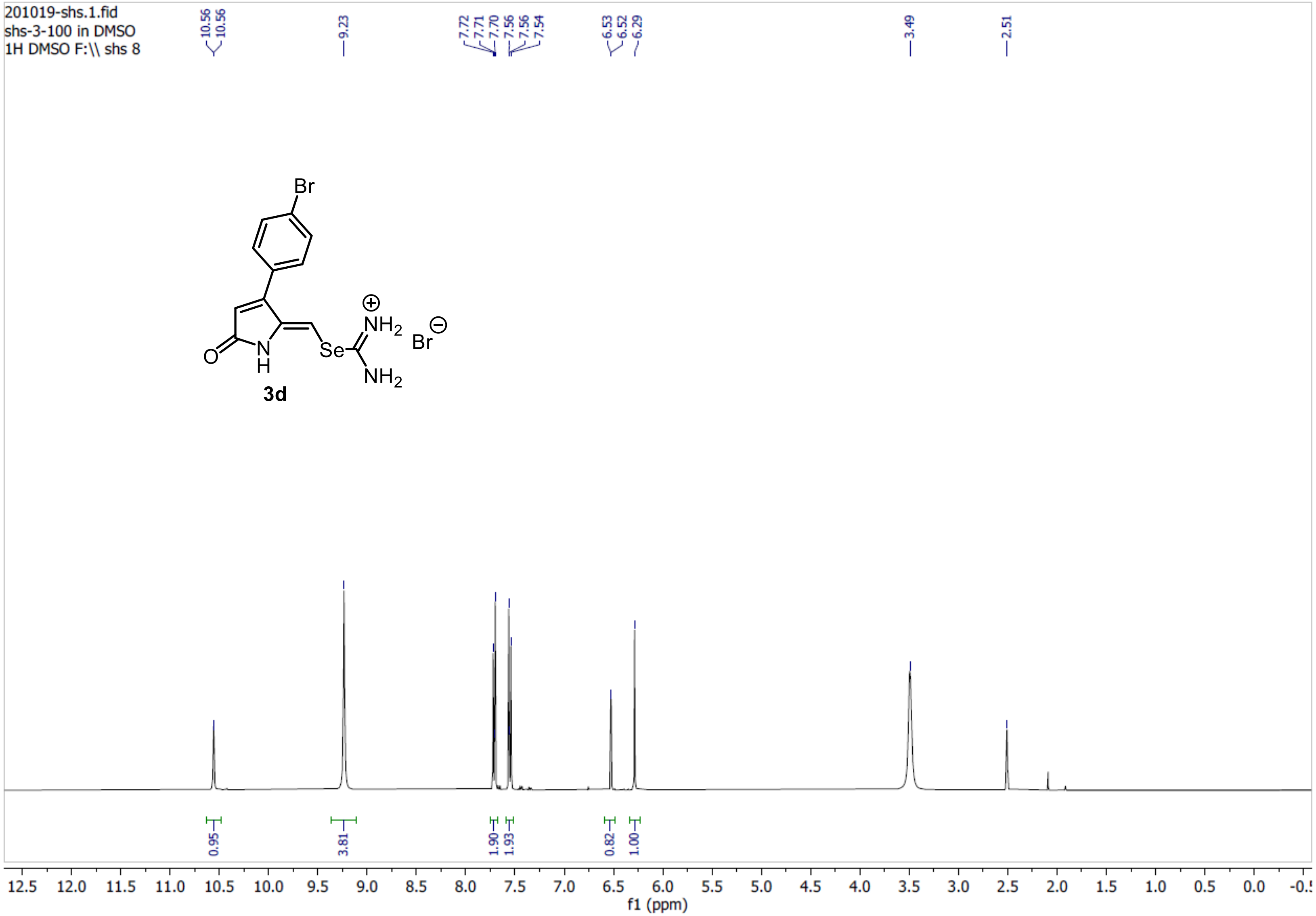

^{13}C NMR spectrum of compound **3d**

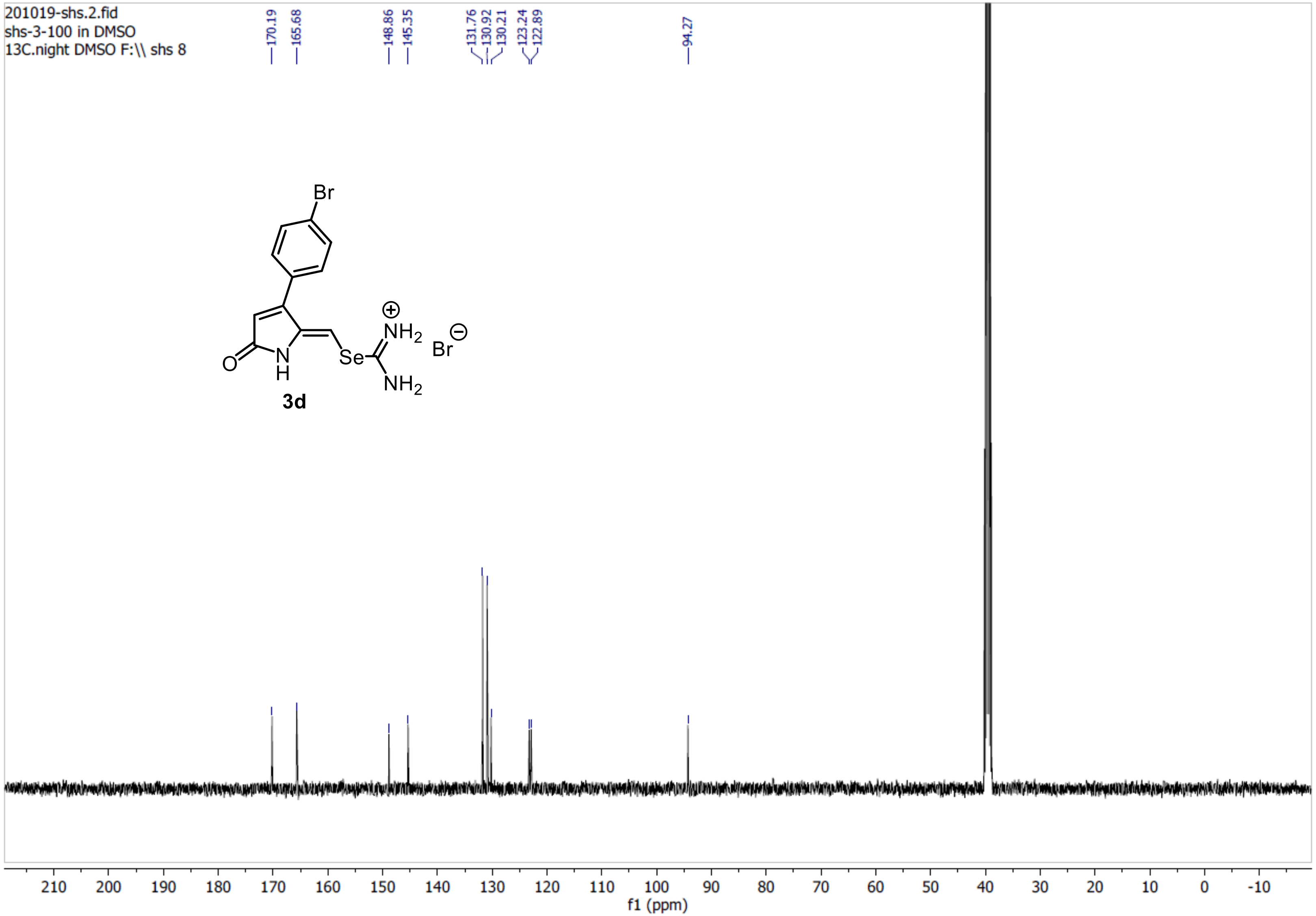

^{1}H NMR spectrum of compound **3e**

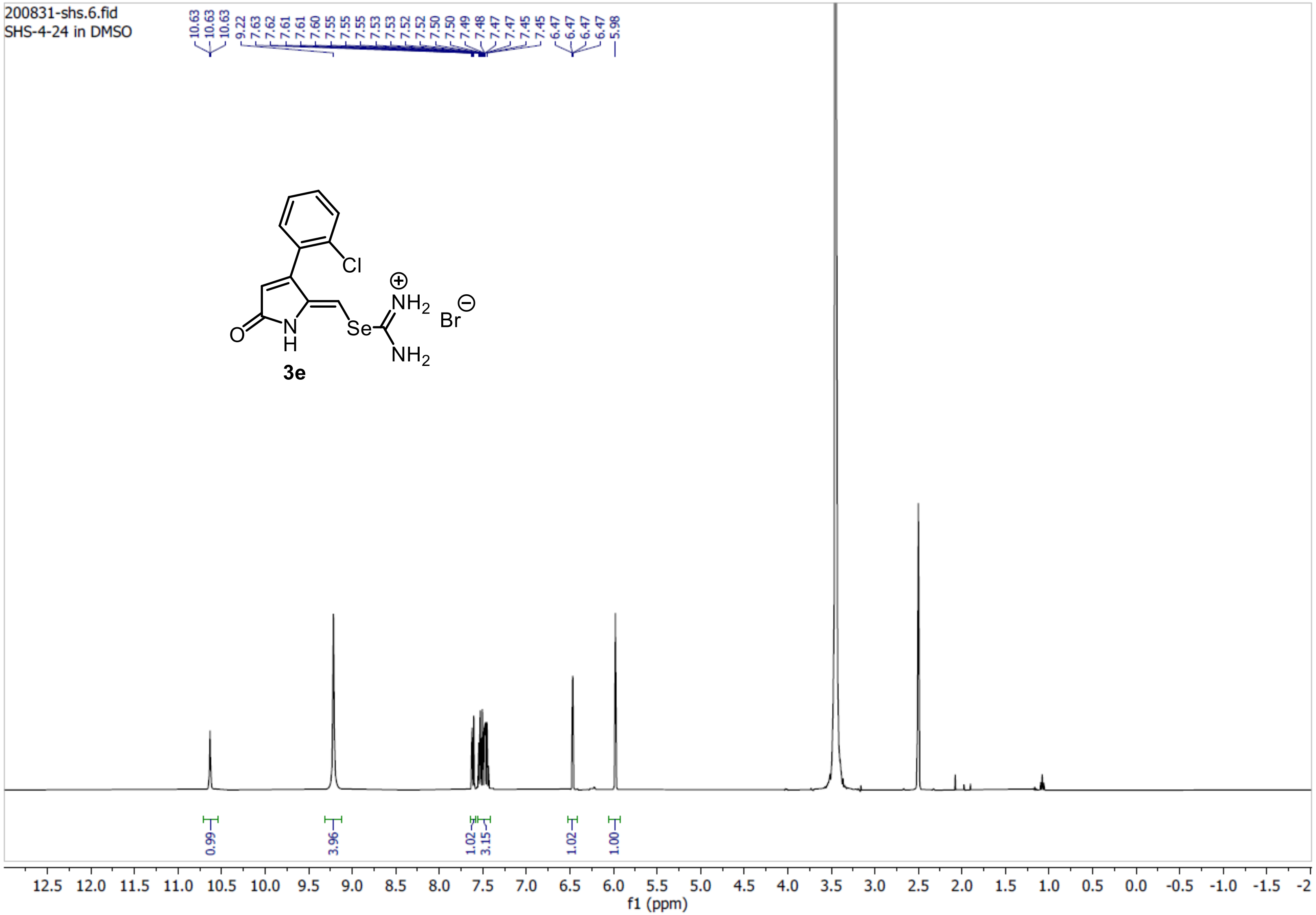

^{13}C NMR spectrum of compound **3e**

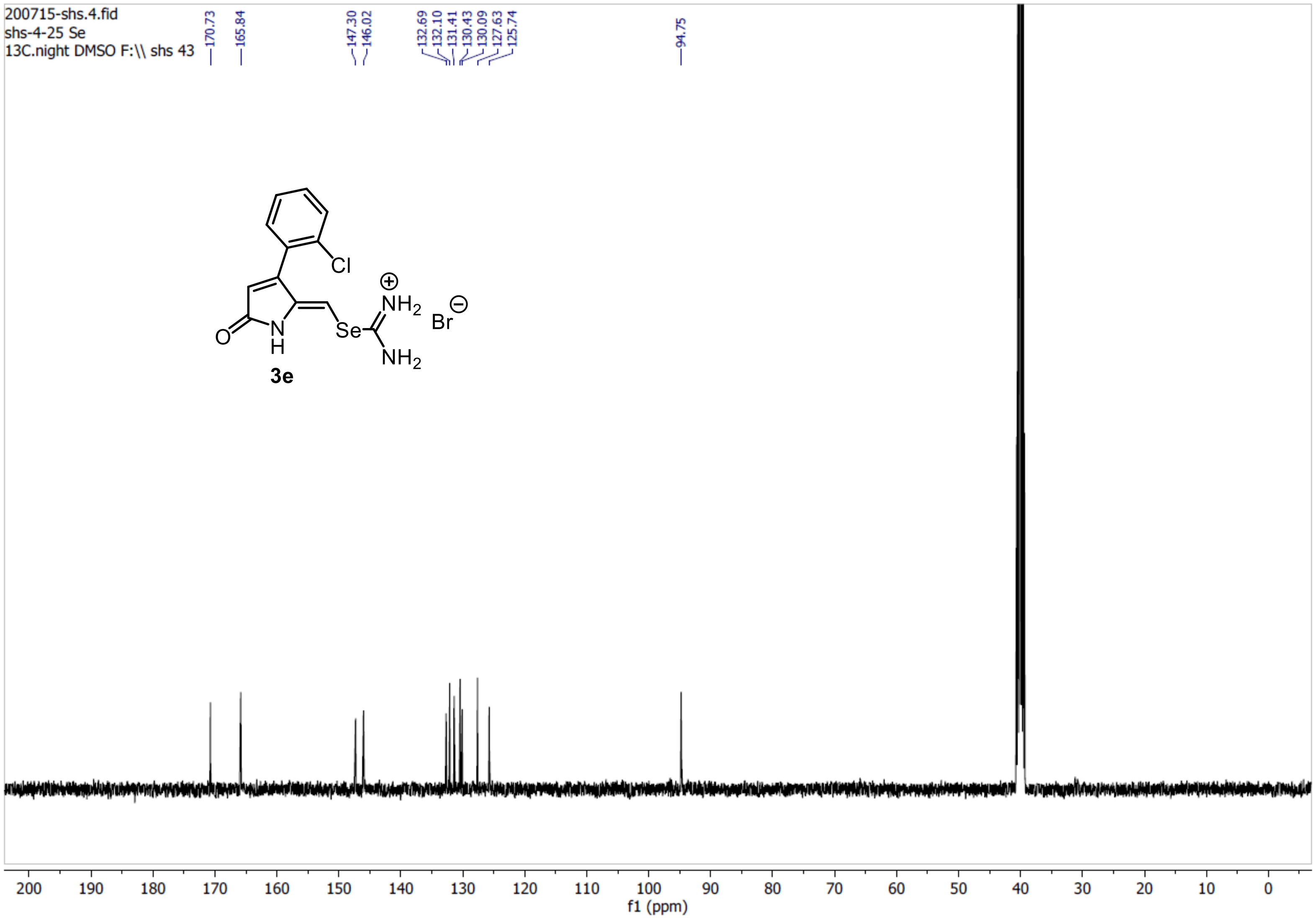

^{77}Se NMR spectrum of compound **3e**

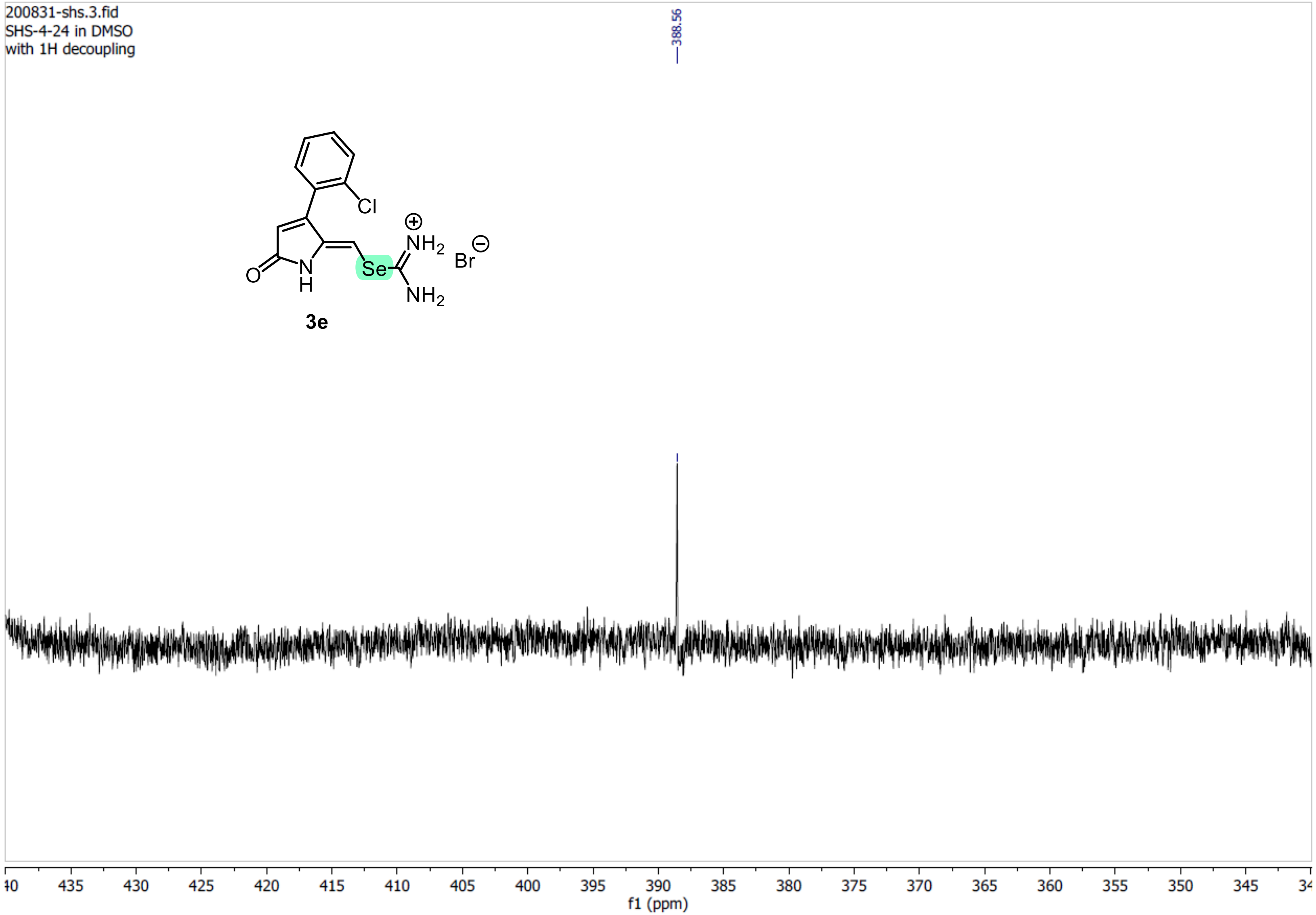

^{1}H NMR spectrum of compound **3f**

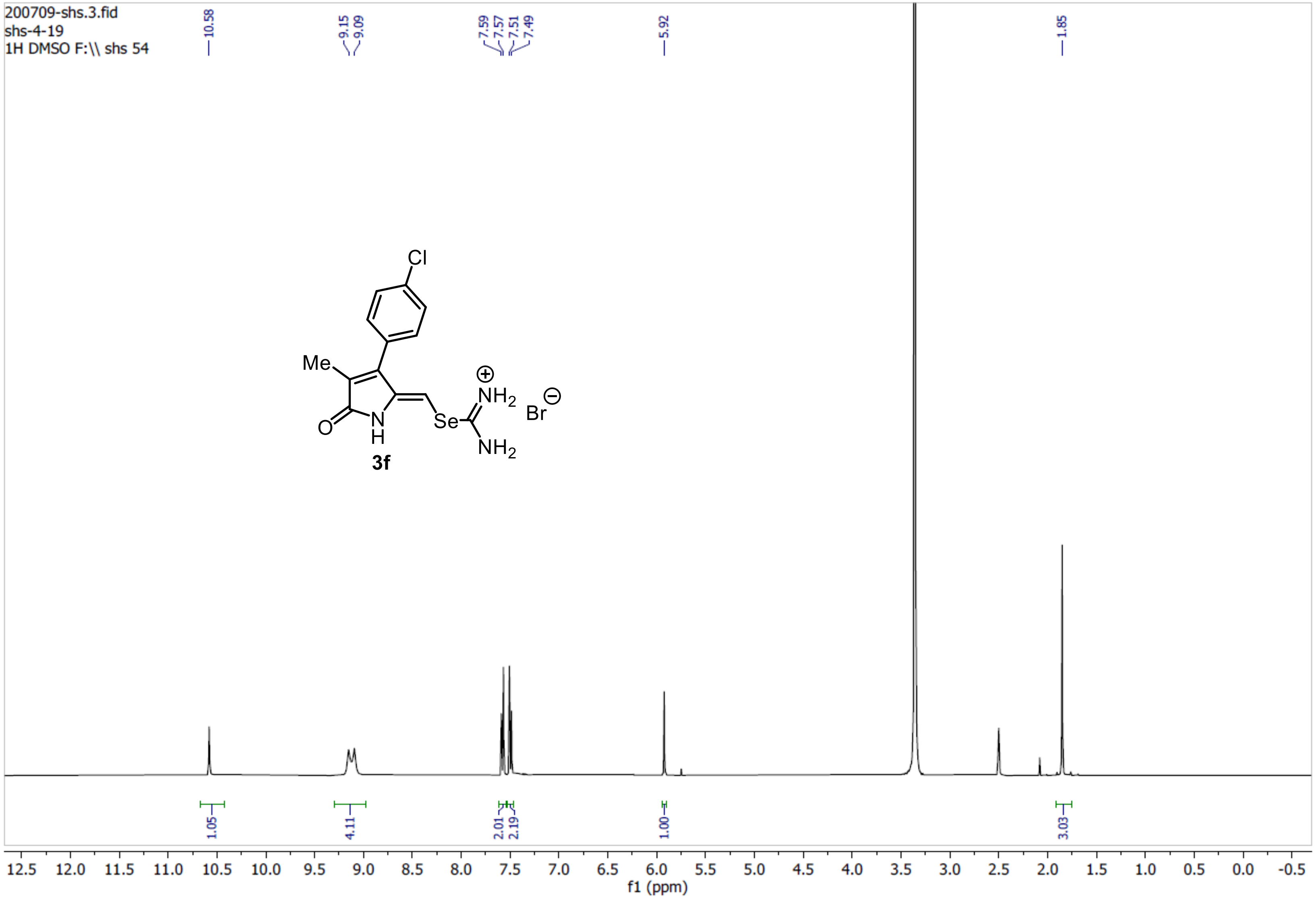

^{13}C NMR spectrum of compound **3f**

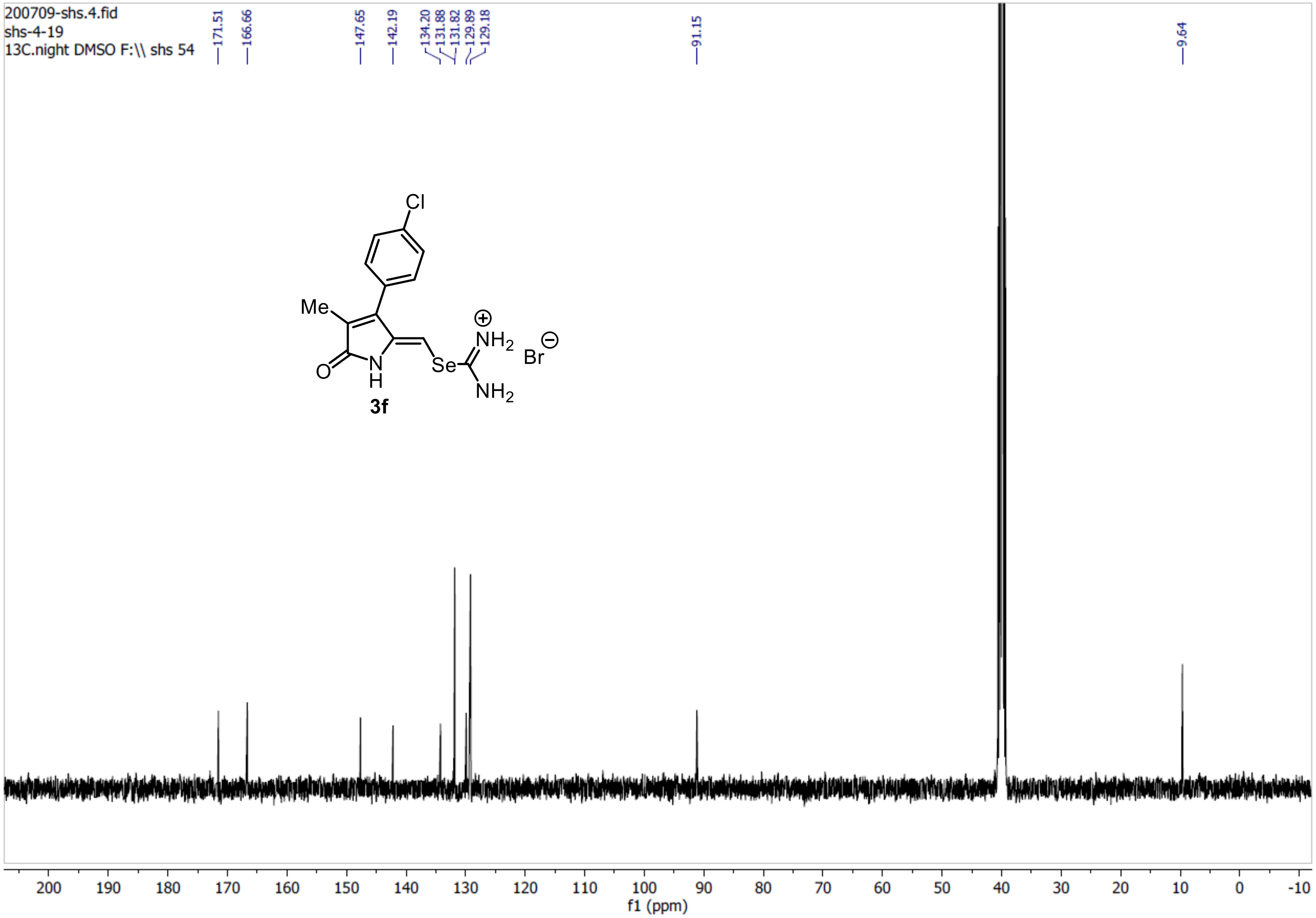

^{1}H NMR spectrum of compound **3g**

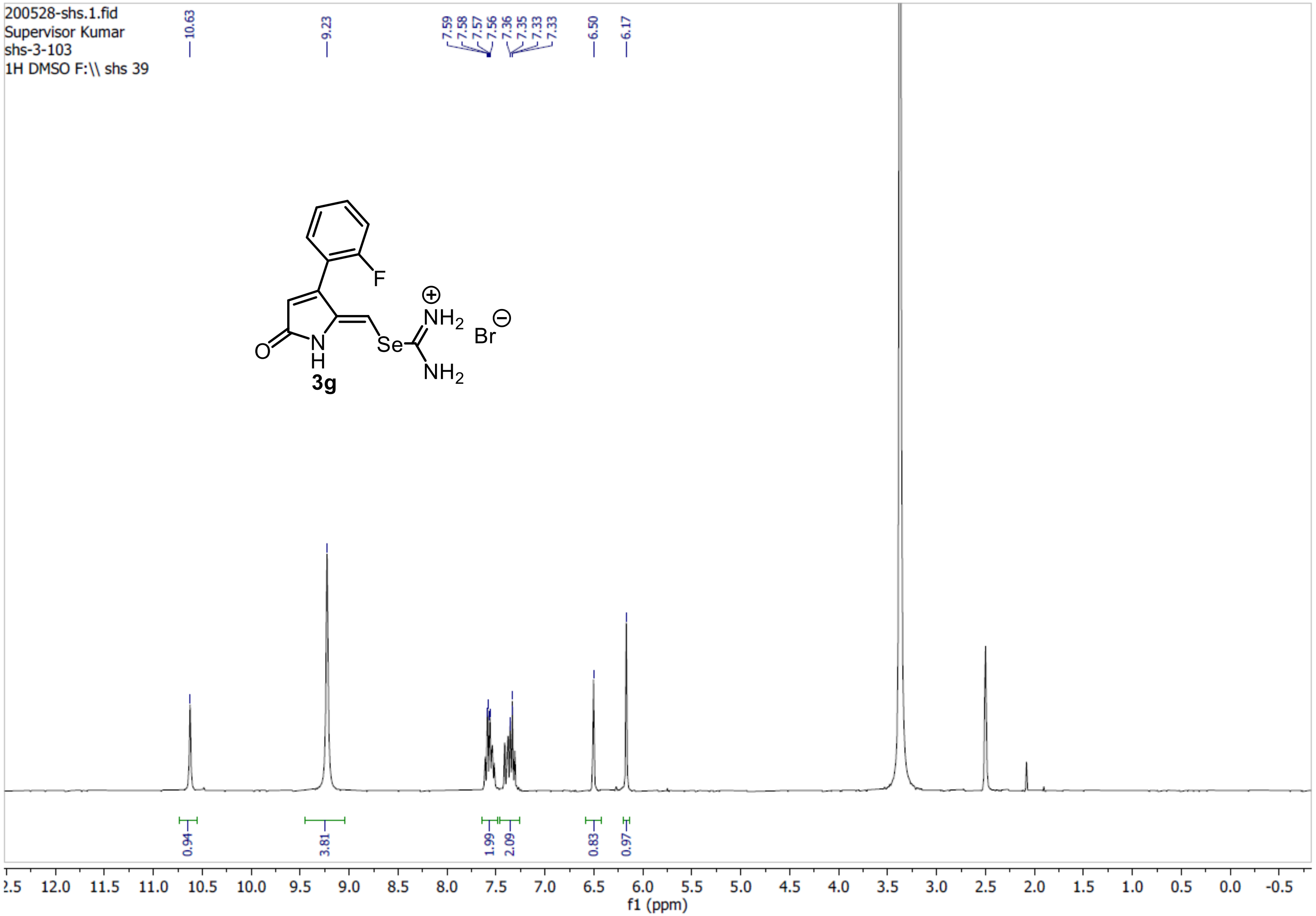

^{13}C NMR spectrum of compound **3g**

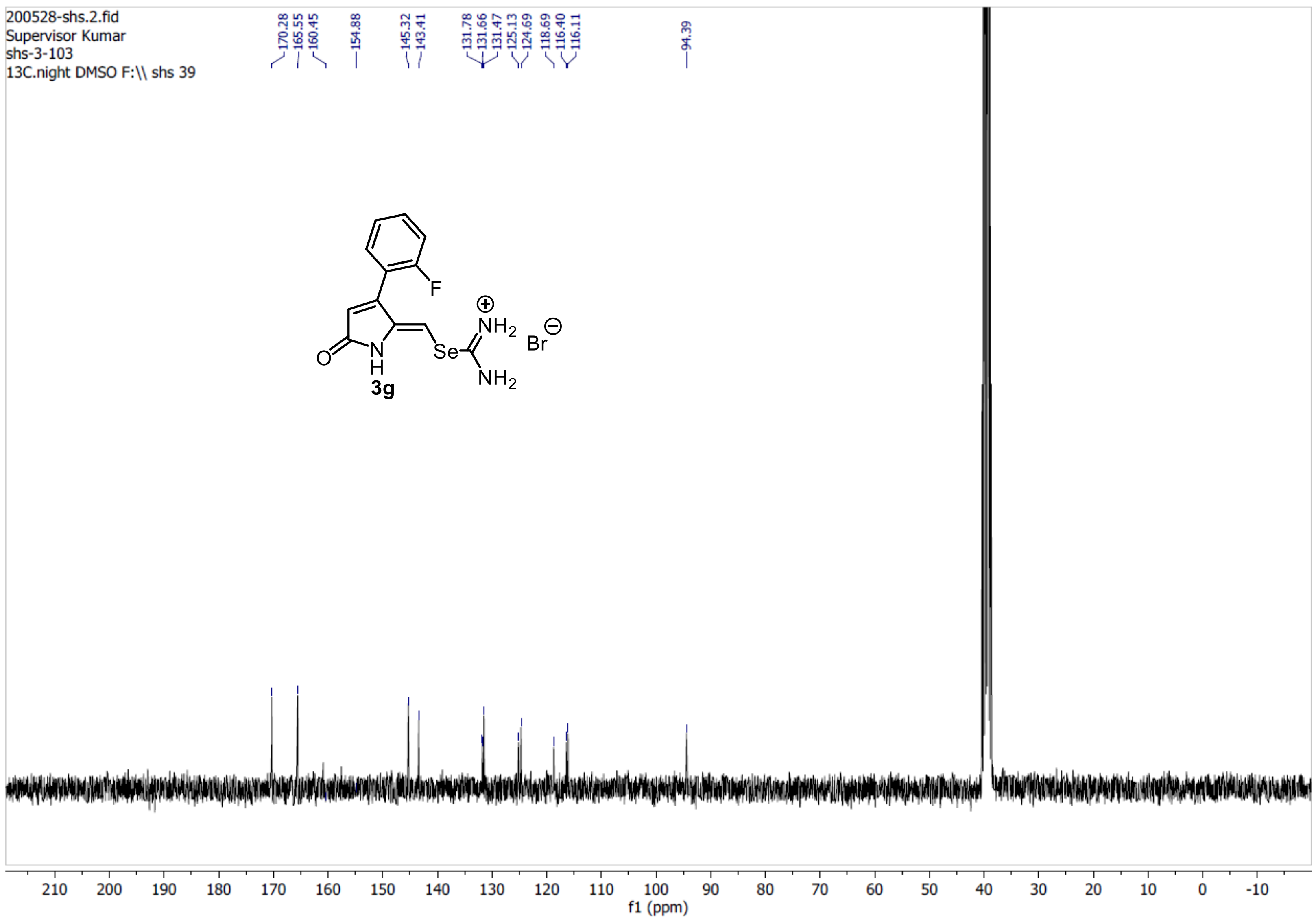

^{1}H NMR spectrum of compound **3h**

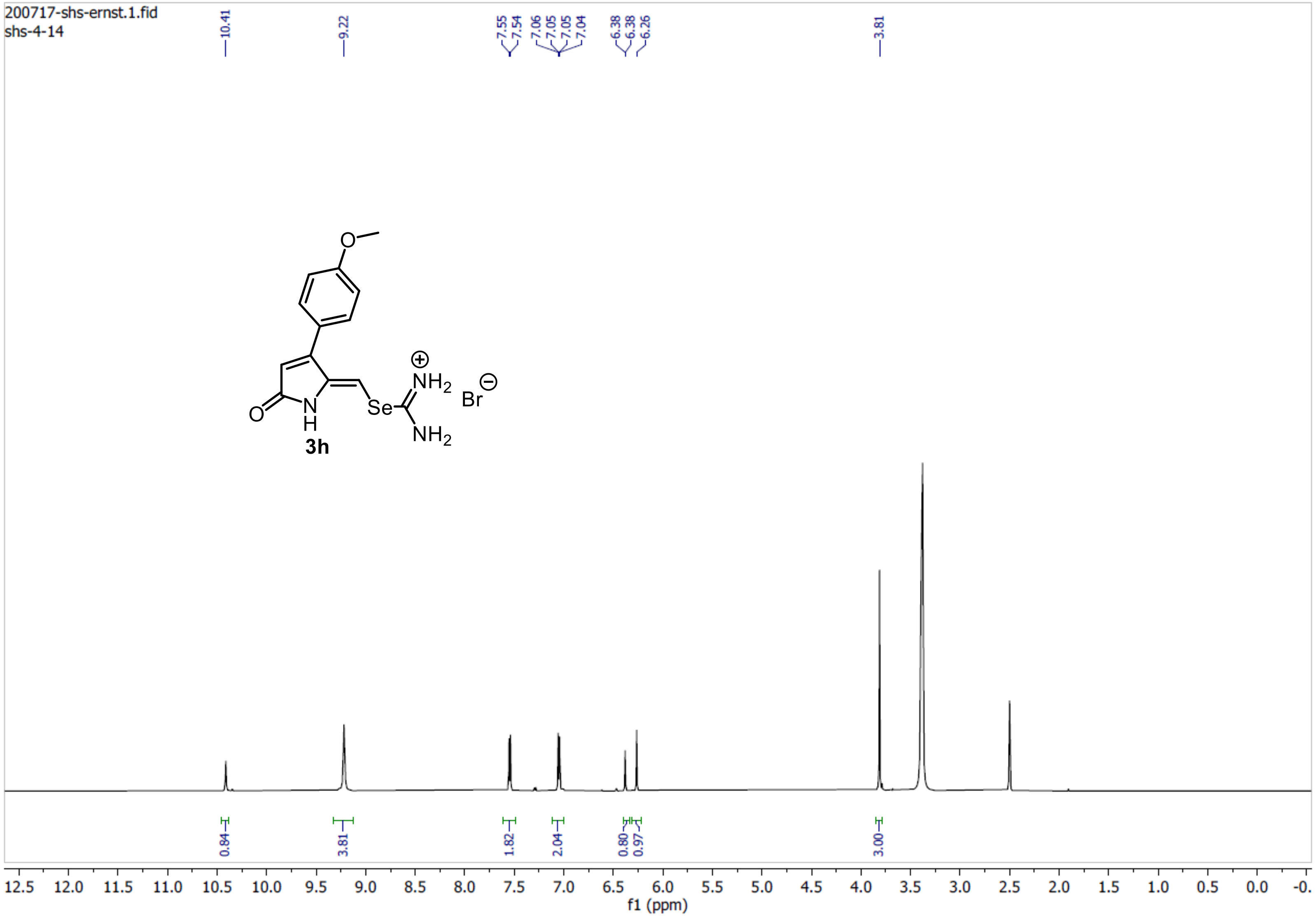

^{13}C NMR spectrum of compound **3h**

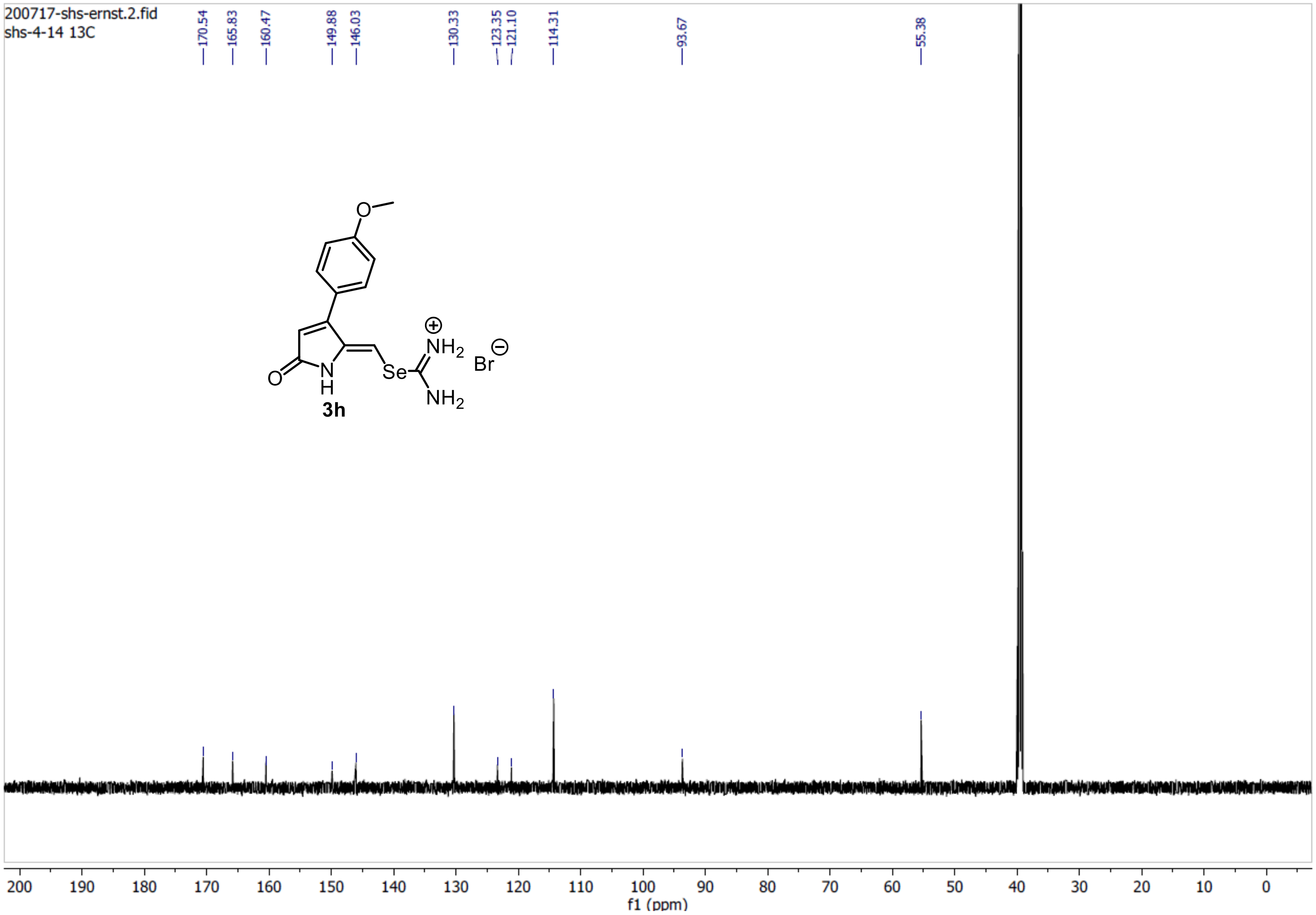

^{1}H NMR spectrum of compound **4a**

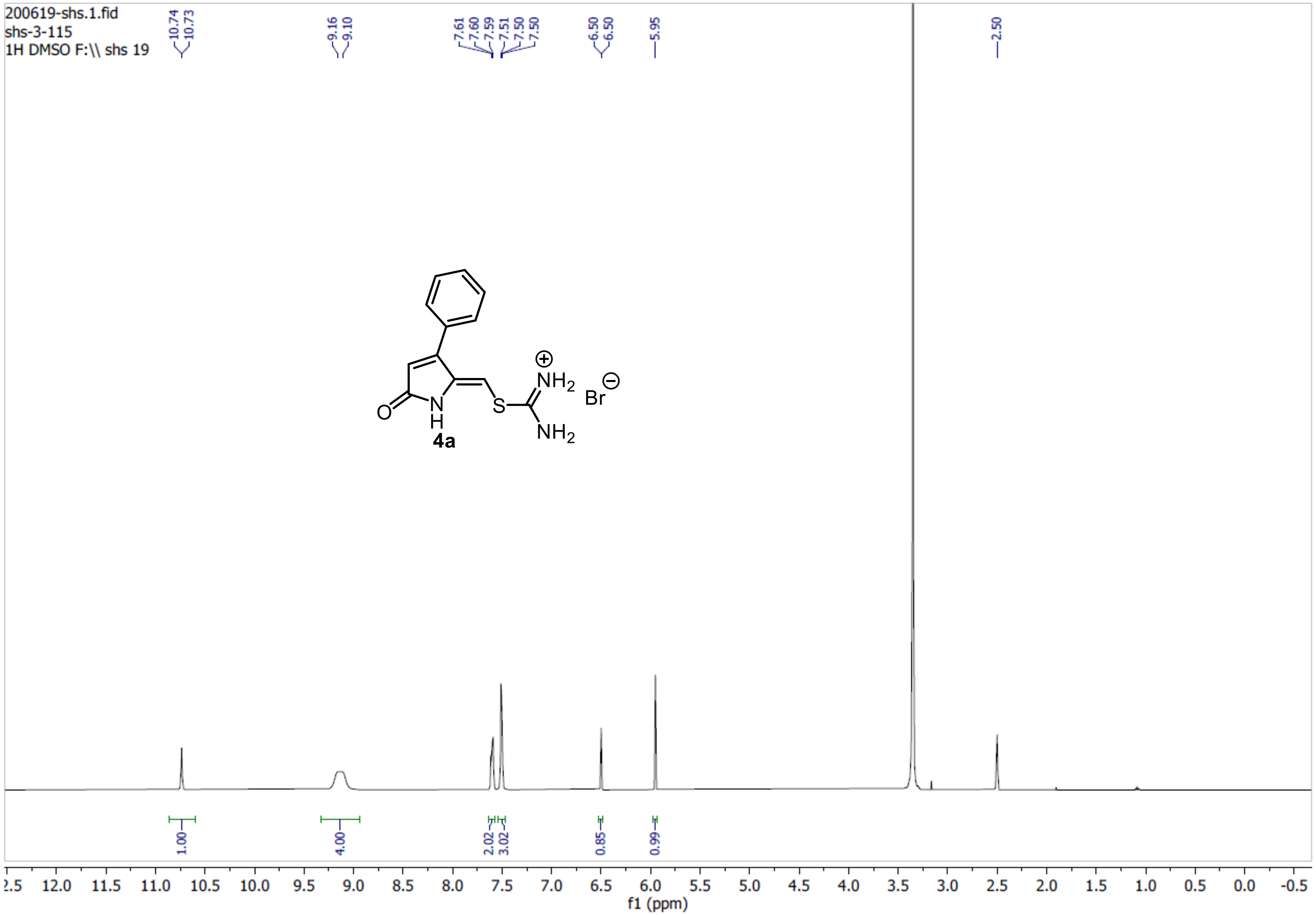

^{13}C NMR spectrum of compound **4a**

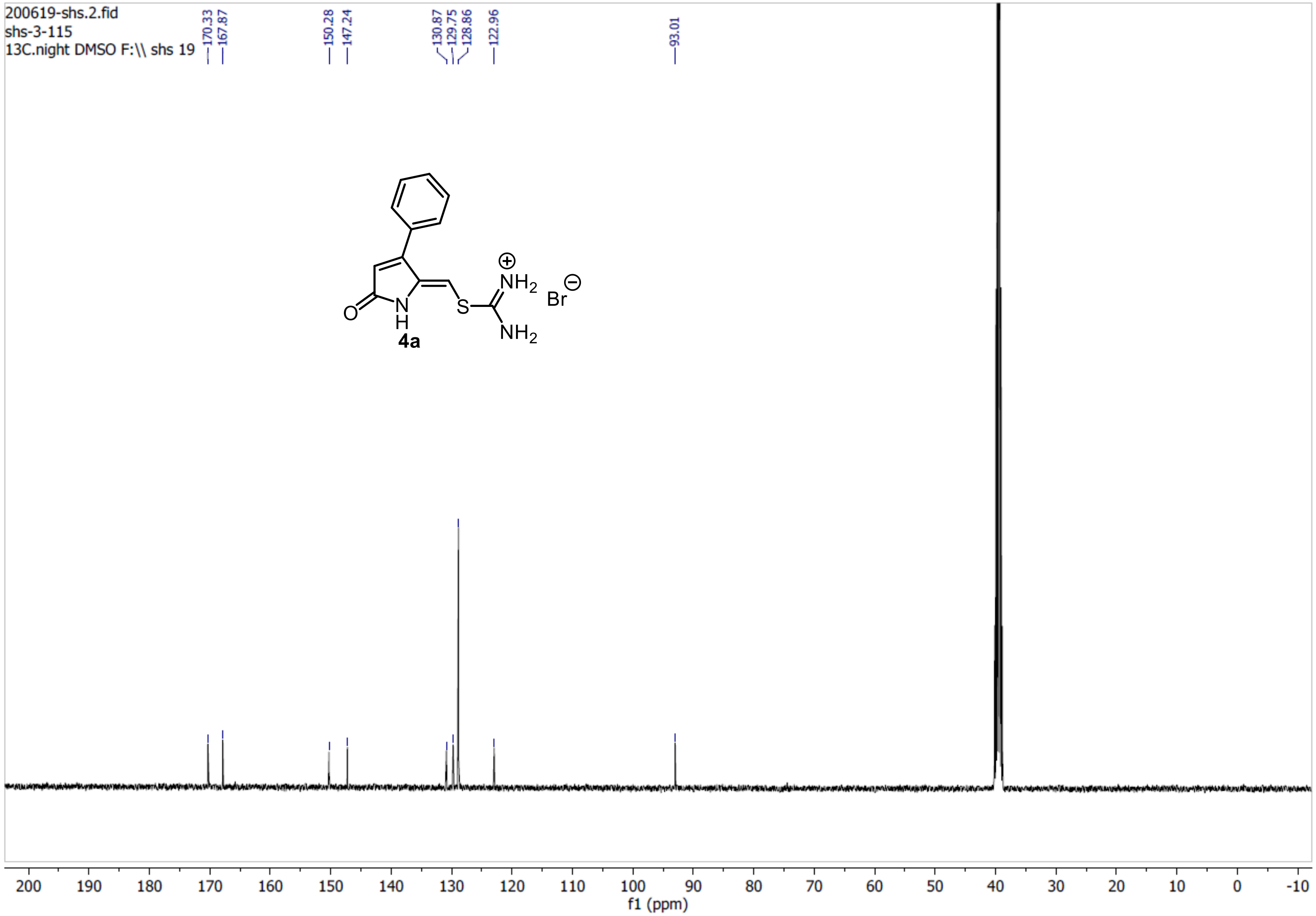

^{1}H NMR spectrum of compound **4b**

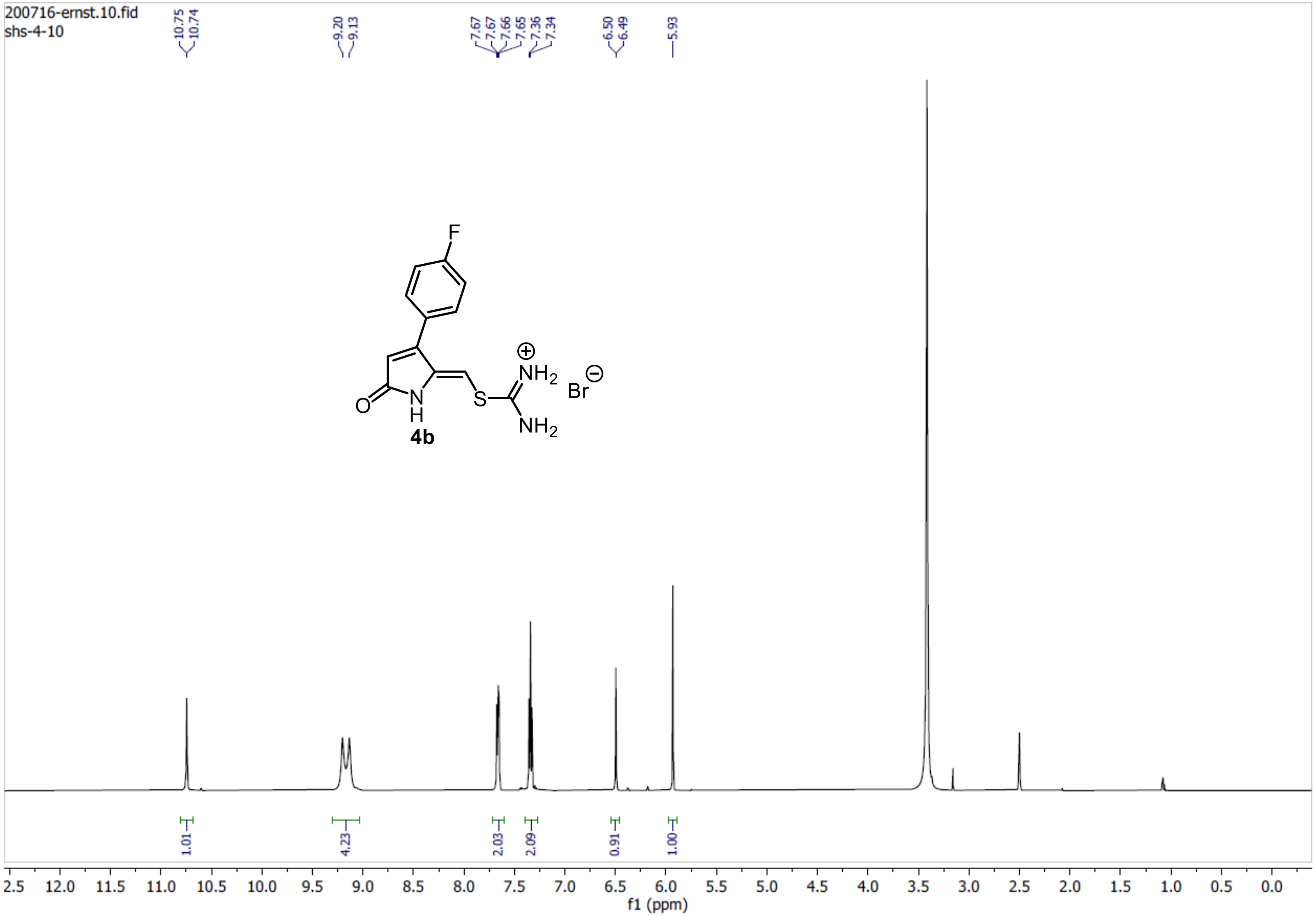

^{13}C NMR spectrum of compound **4b**

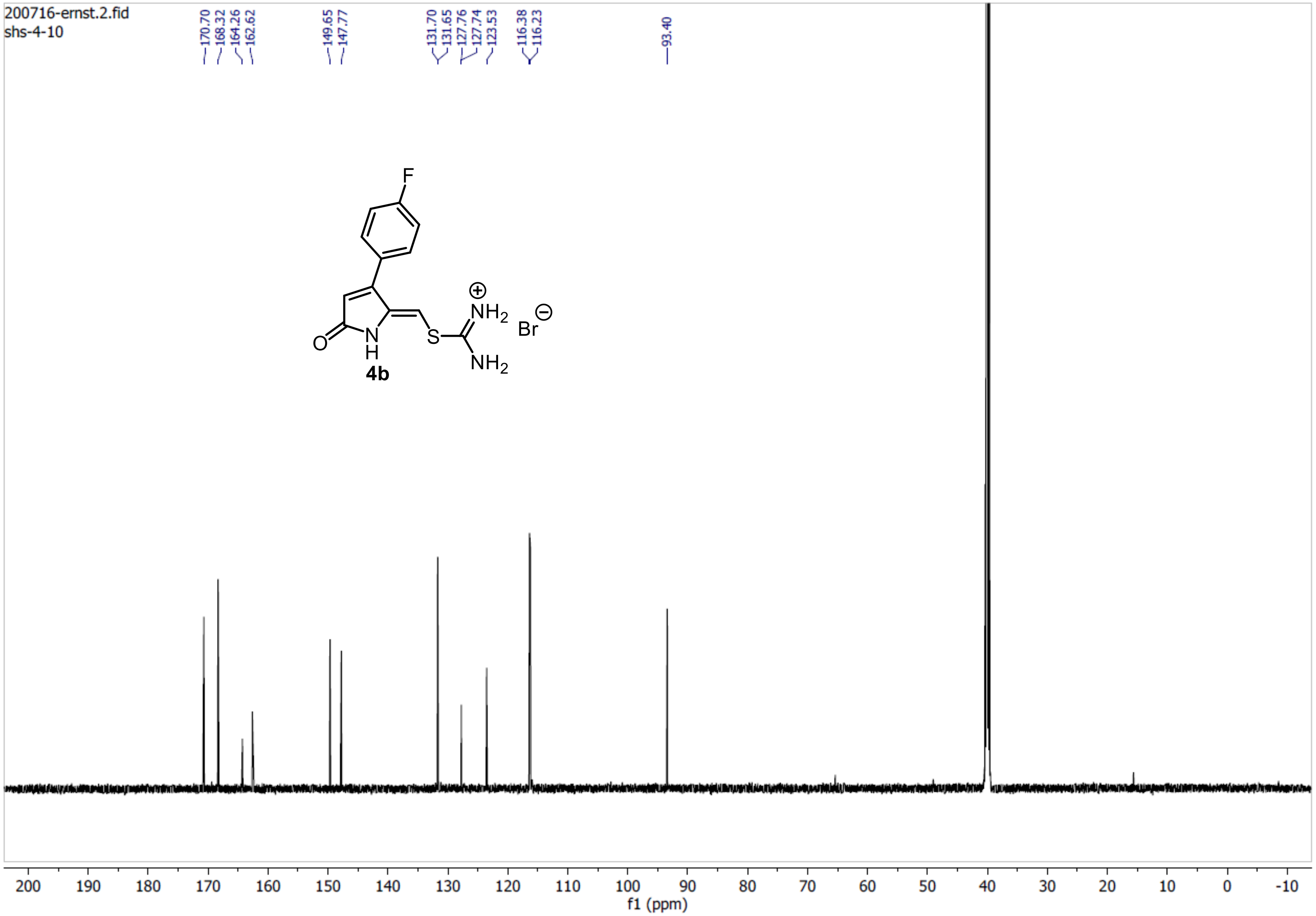

^{1}H NMR spectrum of compound **4c**

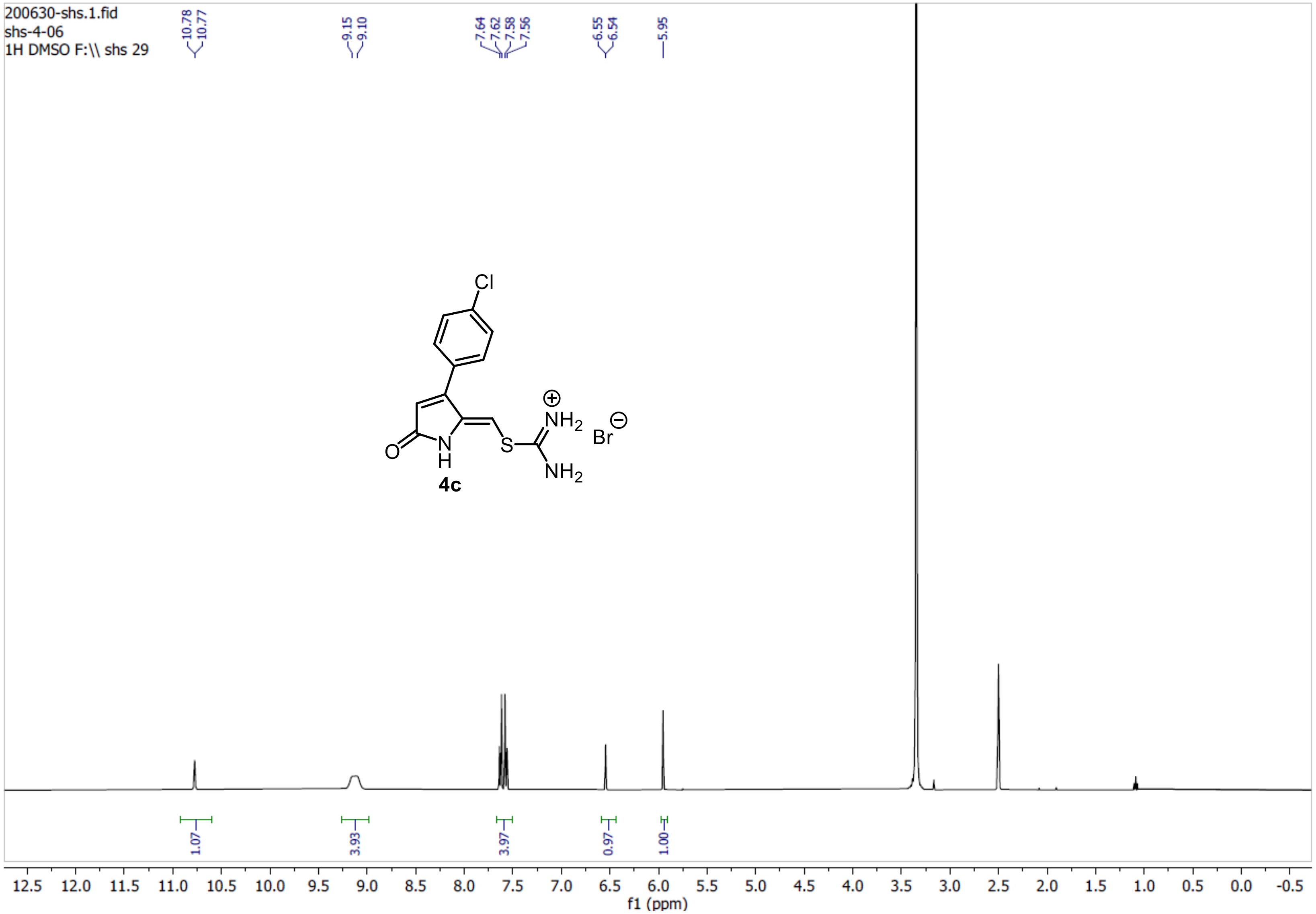

^{13}C NMR spectrum of compound **4c**

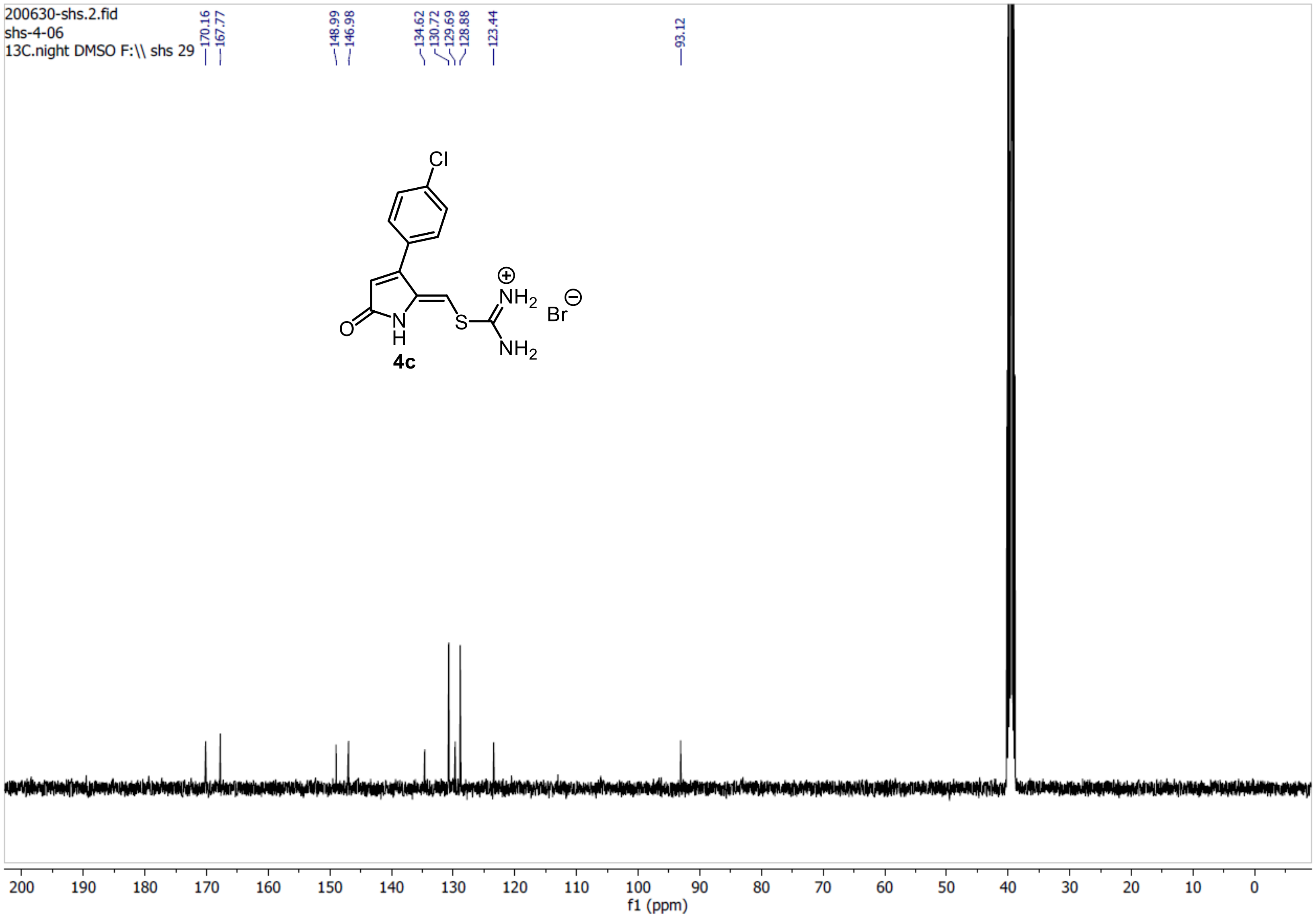

^{1}H NMR spectrum of compound **4d**

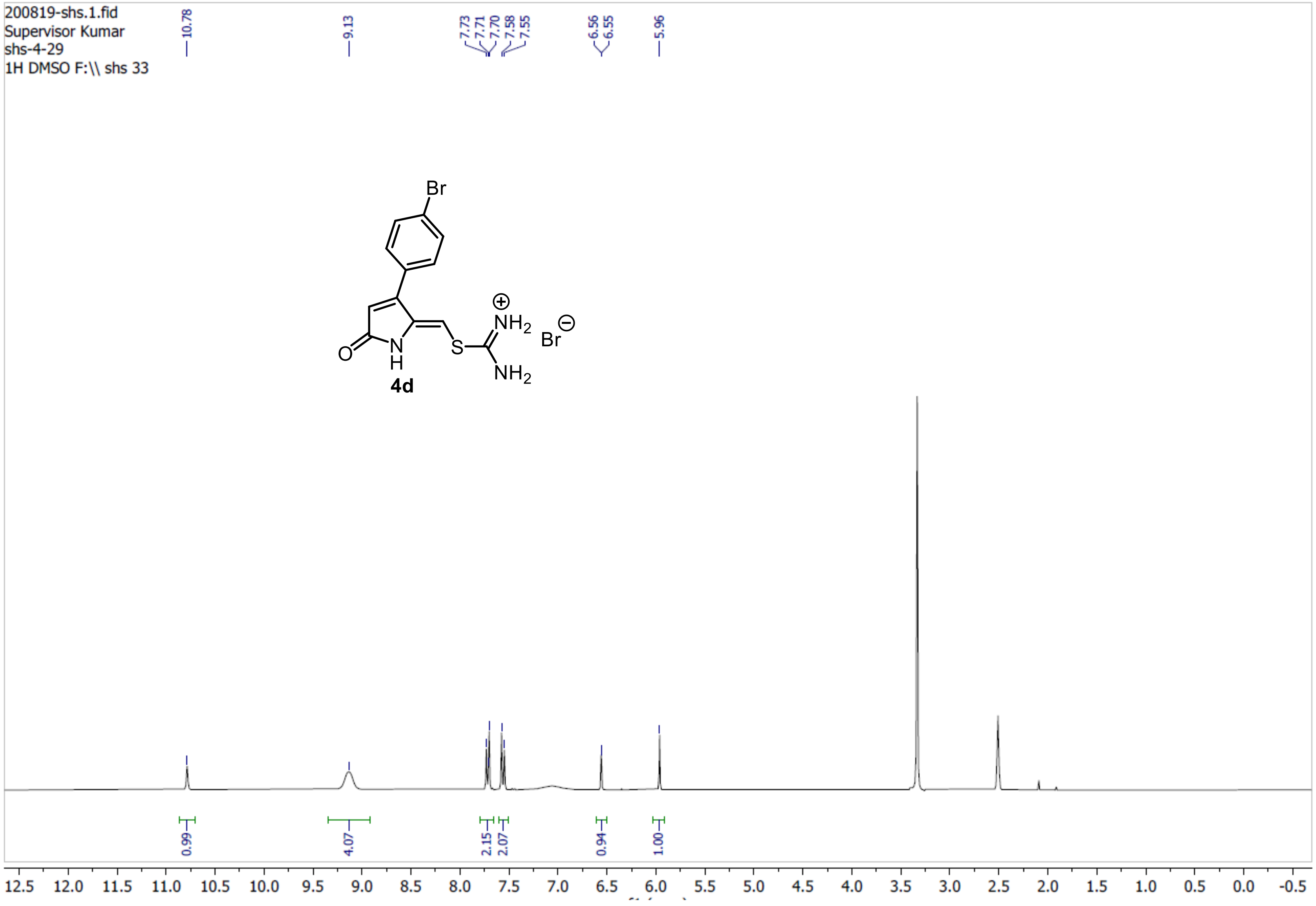

^{13}C NMR spectrum of compound **4d**

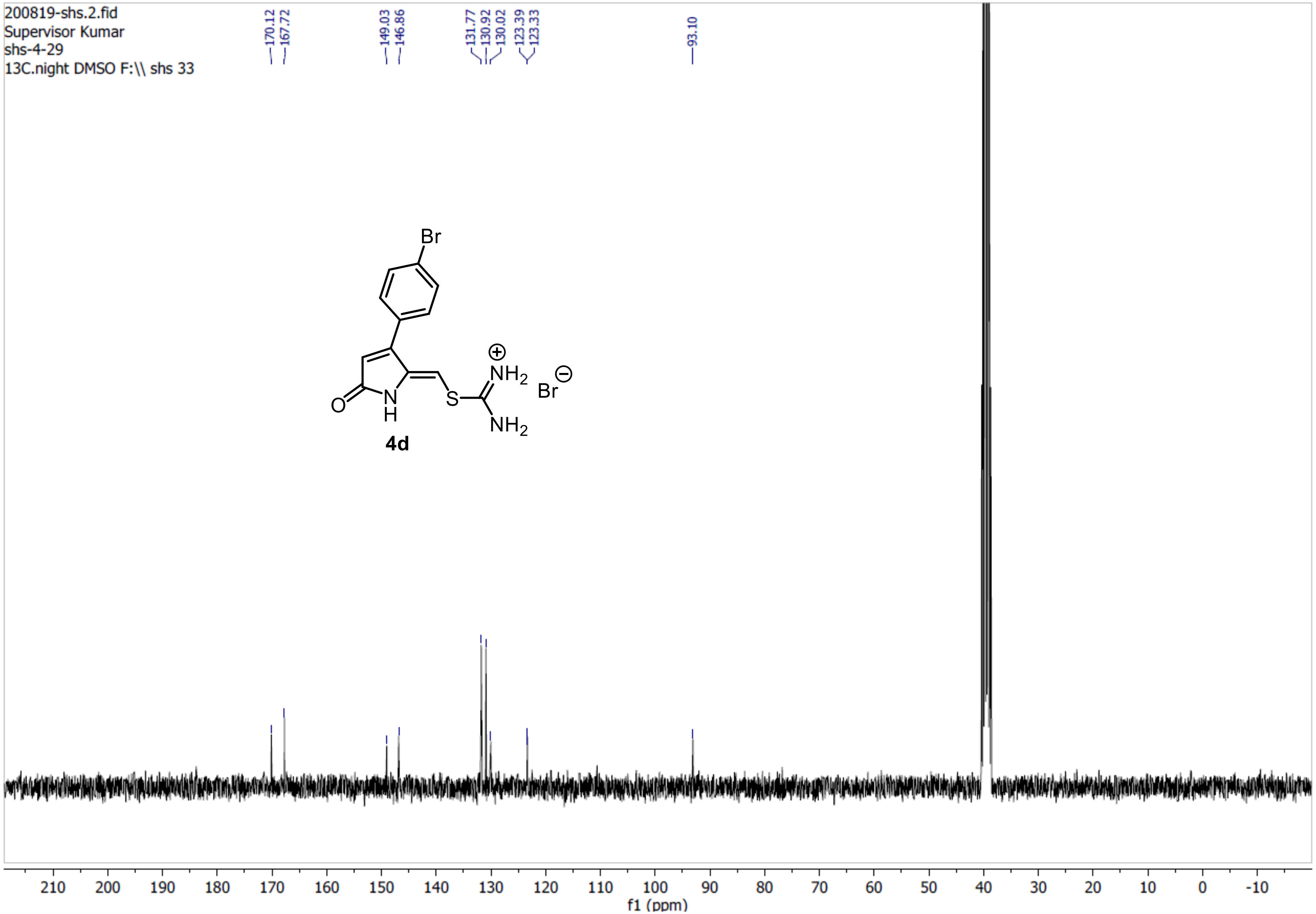

^{1}H NMR spectrum of compound **4e**

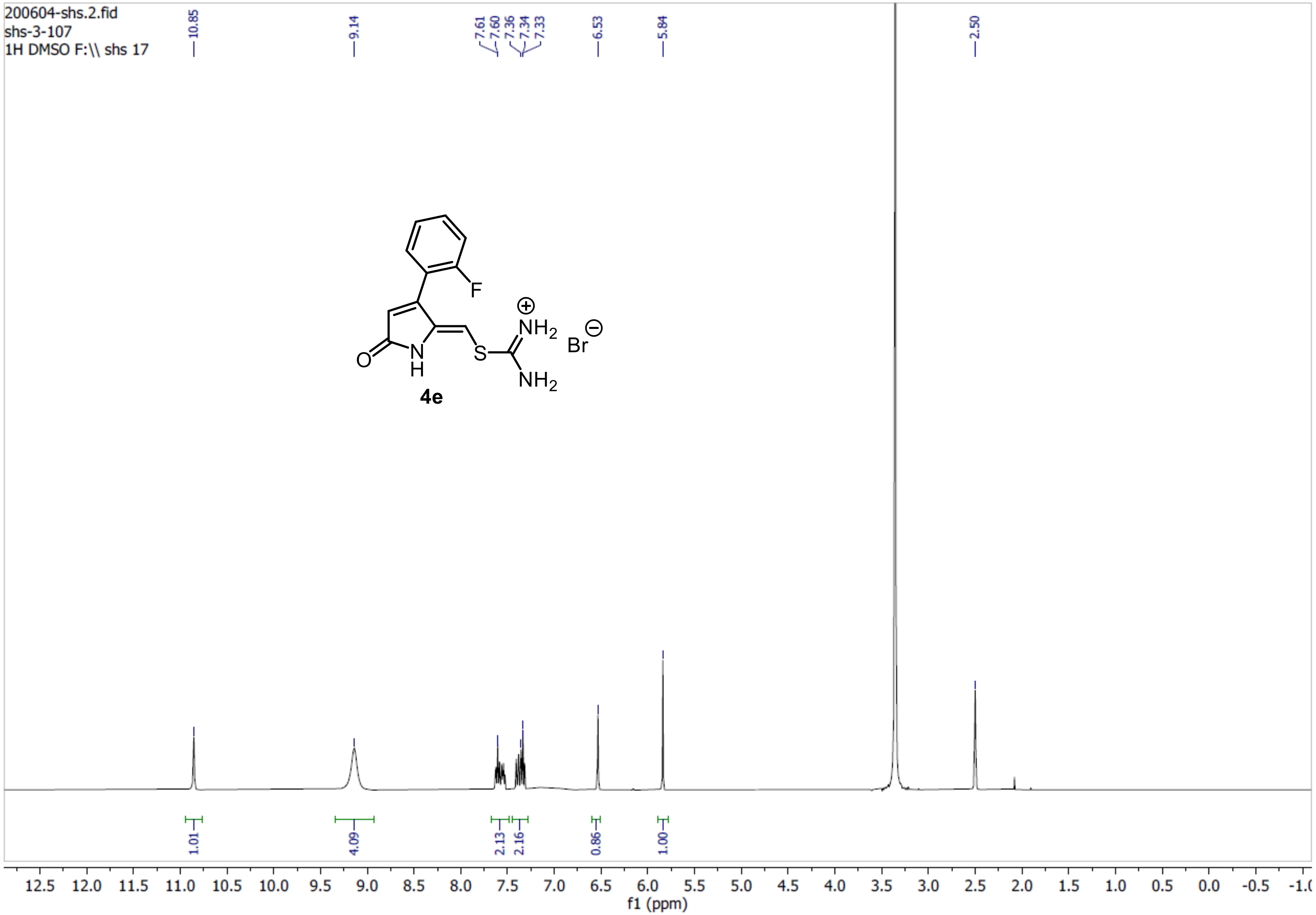

^{13}C NMR spectrum of compound **4e**

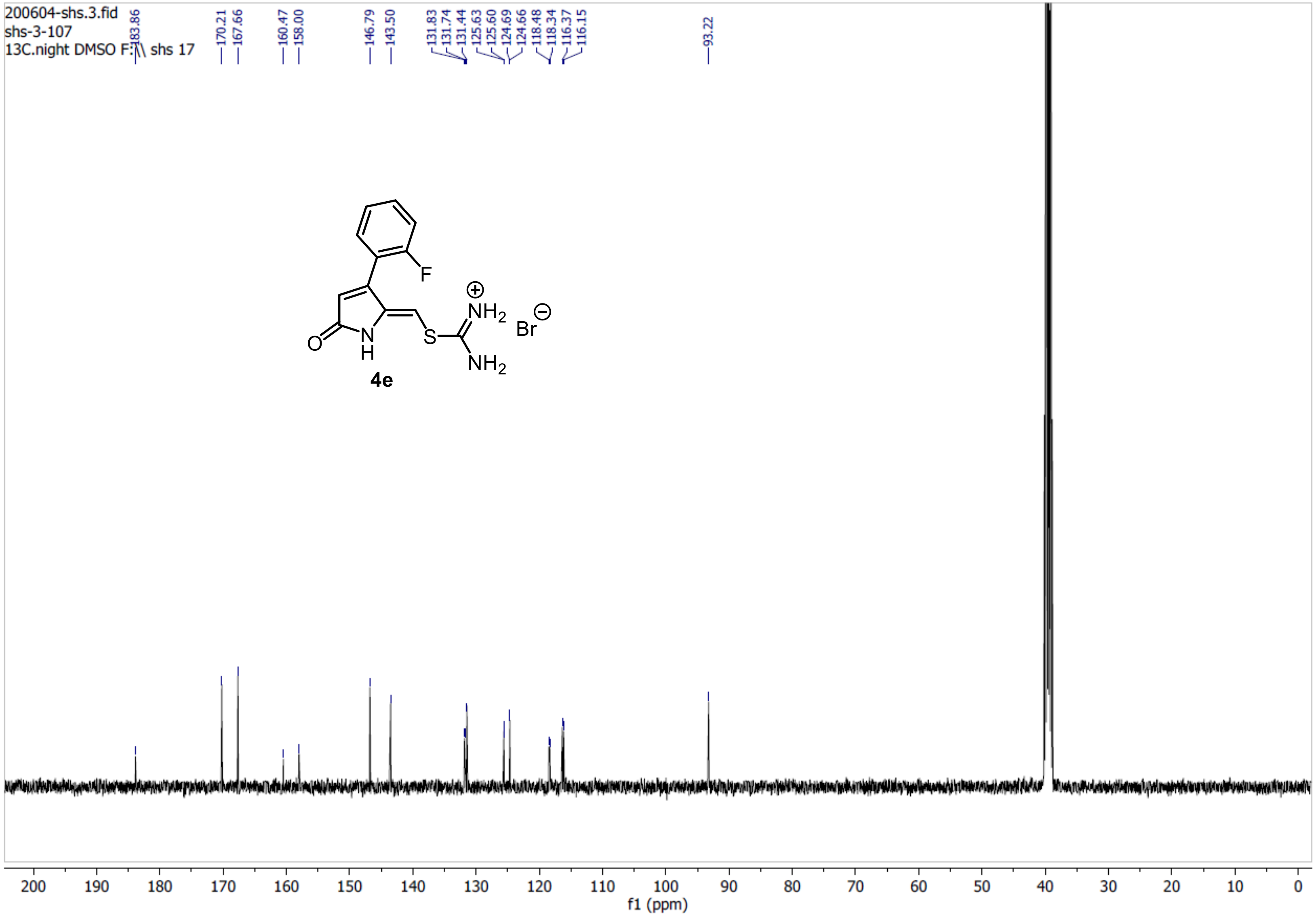

^{1}H NMR spectrum of compound **4f**

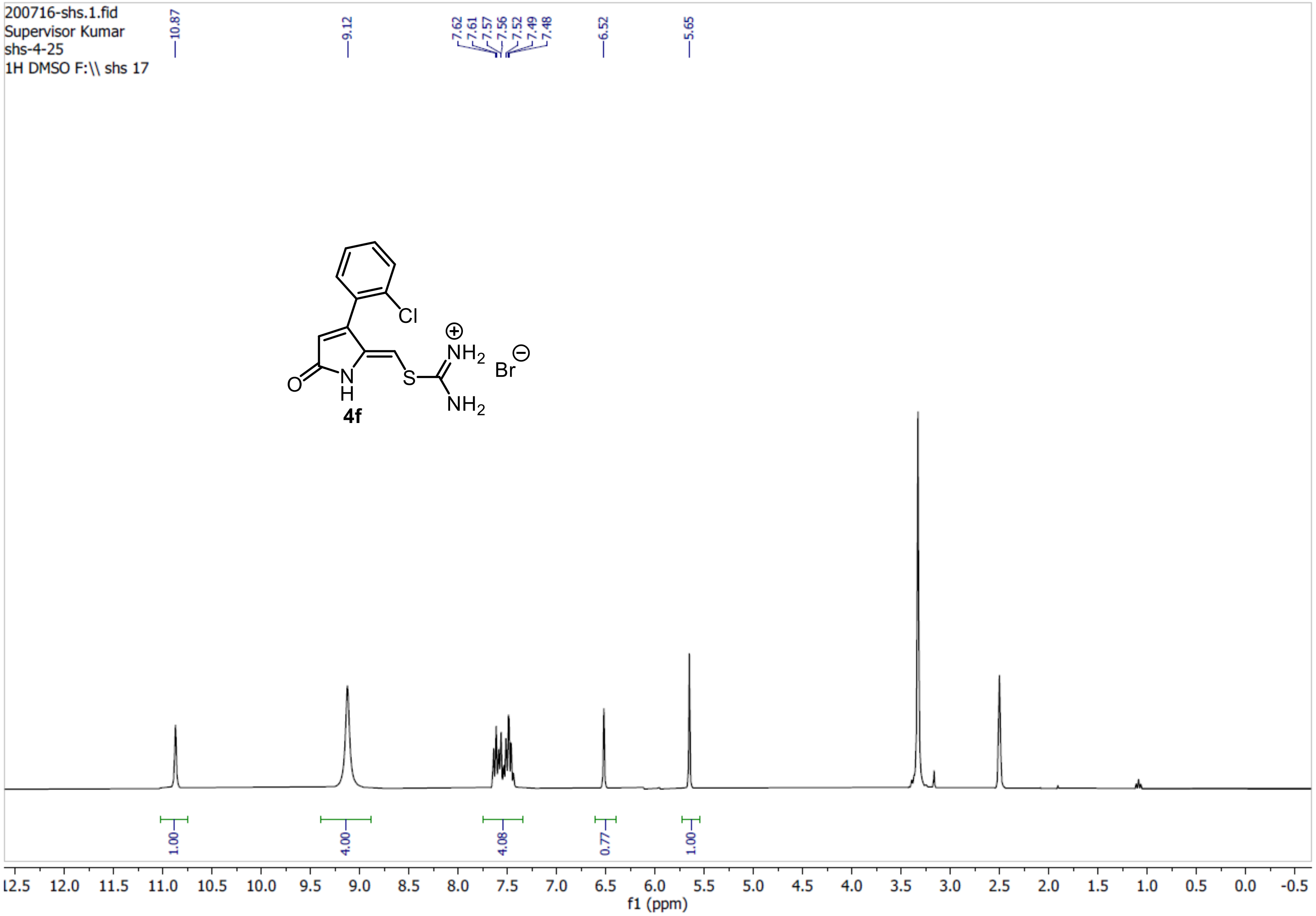

^{13}C NMR spectrum of compound **4f**

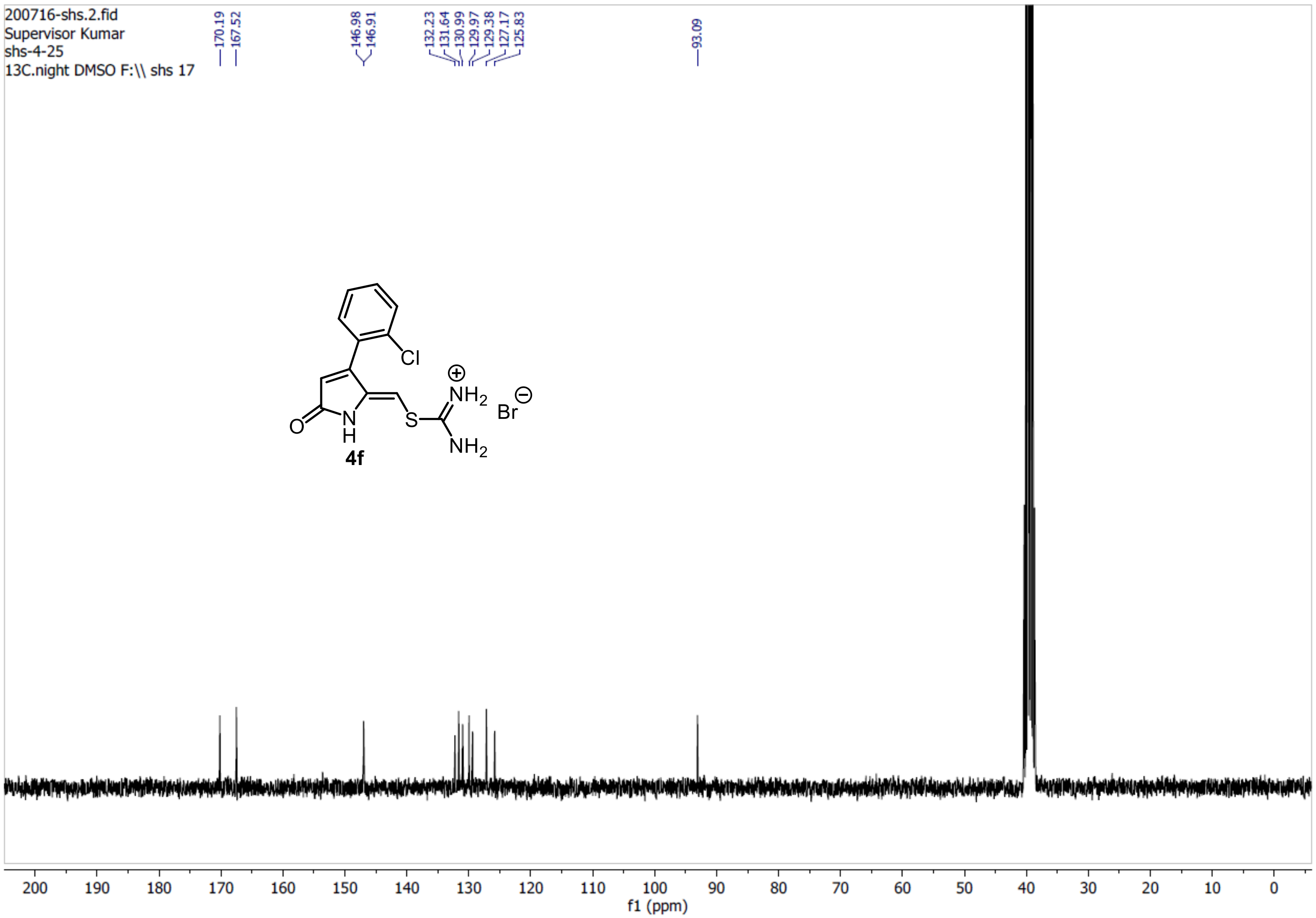

^{1}H NMR spectrum of compound **4g**

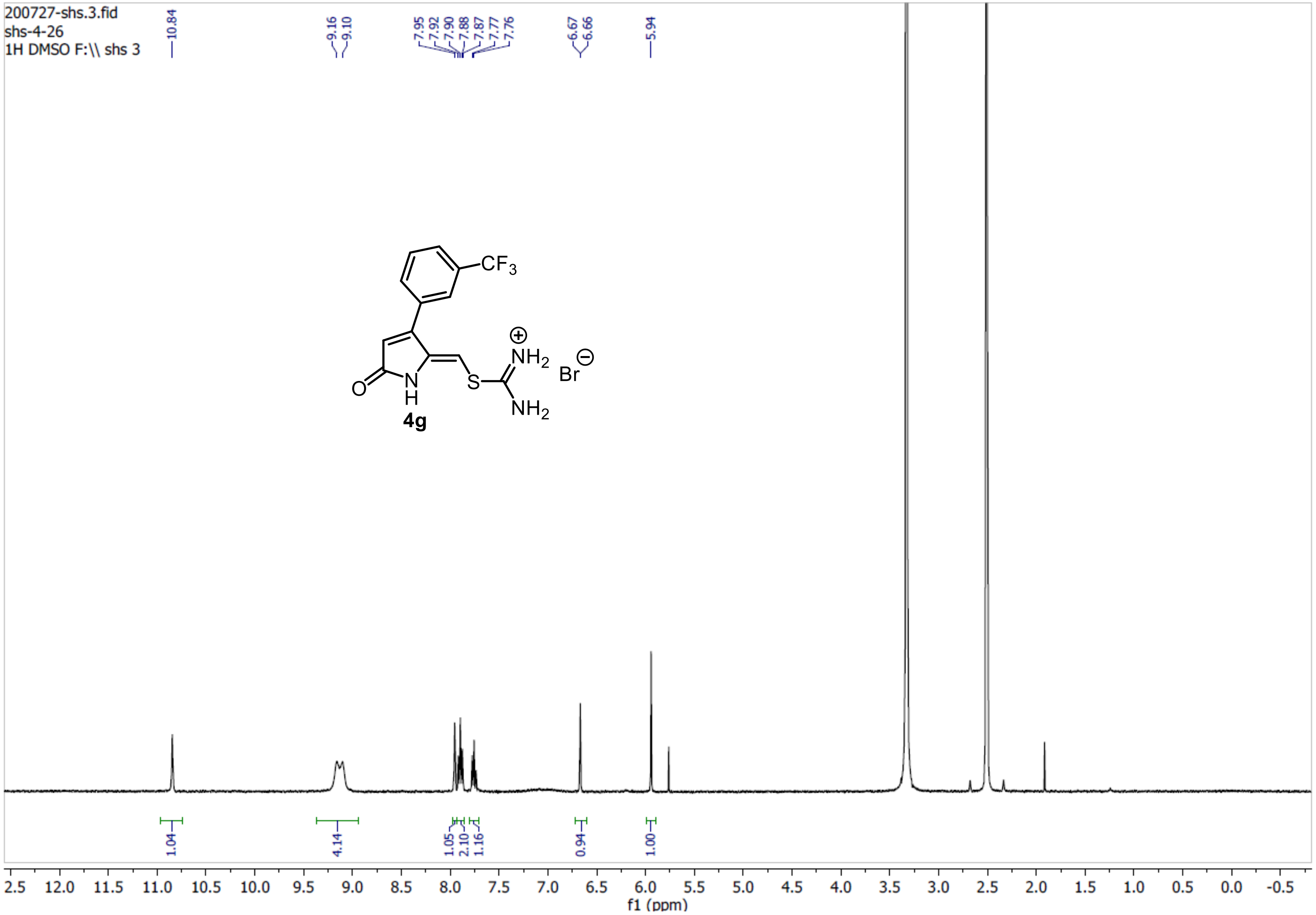

^{13}C NMR spectrum of compound **4g**

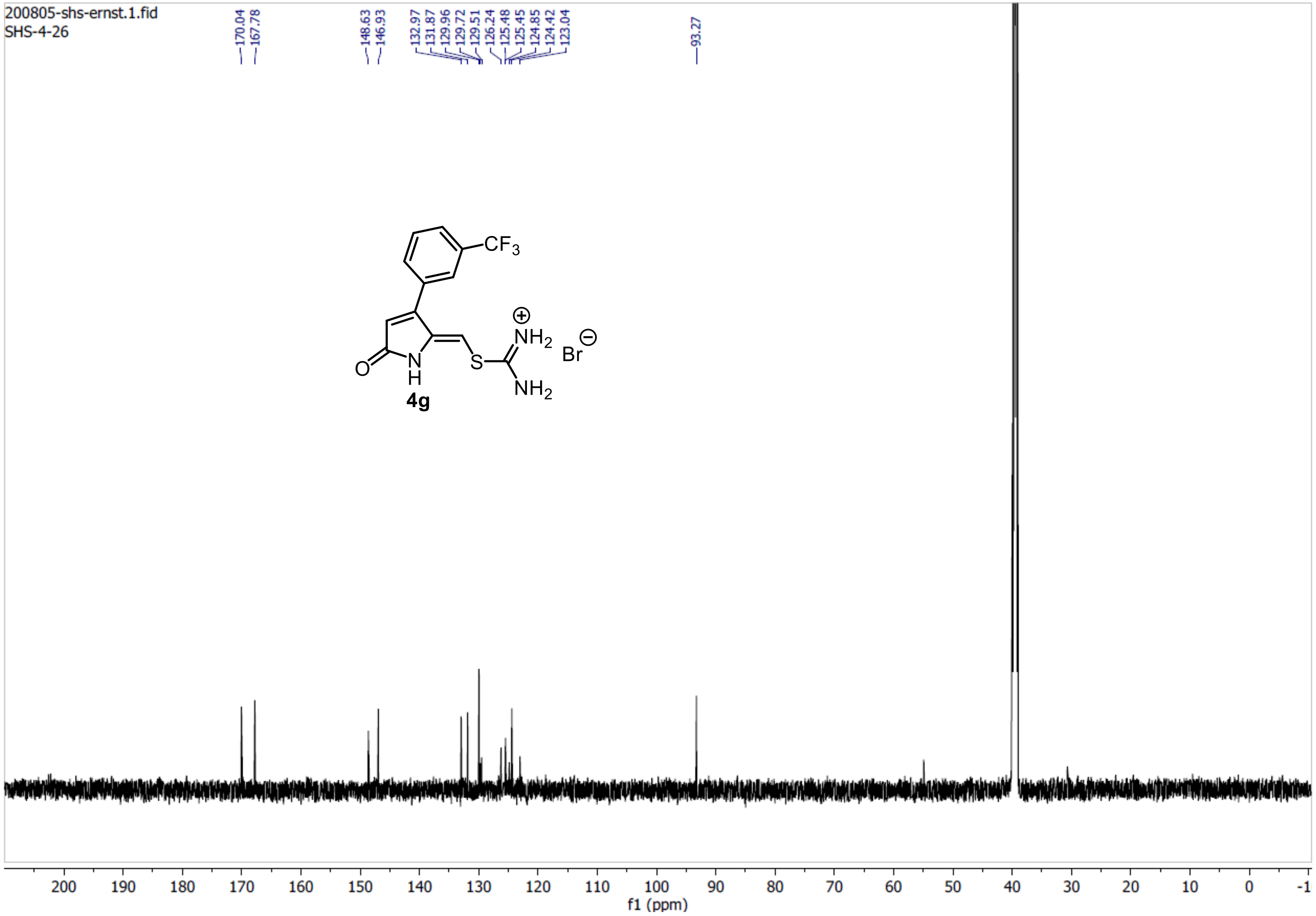

Sample submission report

Sample Analysis Request #	**3073456**
Samples submitted by	**Shekh Sabir**
Date run	2020-11-17
Operator	Chowdhury Sarowar
Report prepared by	Chowdhury Sarowar

Notes:

Sample: shs-3-114

Full spectrum

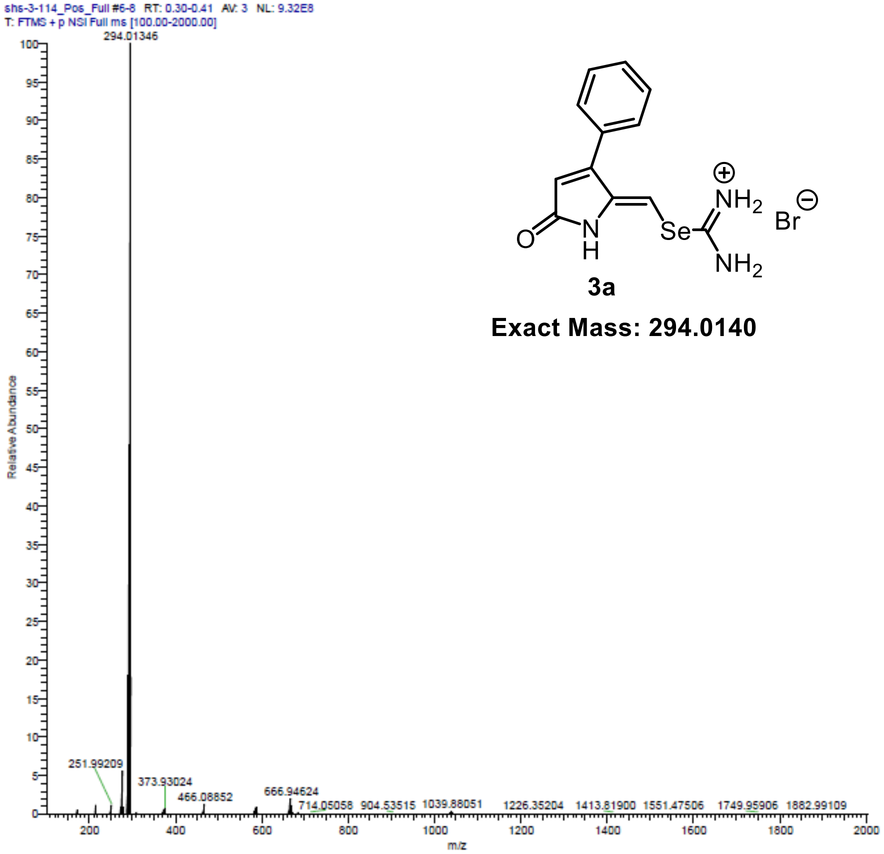

3a

Exact Mass: 294.0140

Sample submission report

Sample Analysis Request #	**3073456**
Samples submitted by	**Shekh Sabir**
Date run	2020-11-17
Operator	Chowdhury Sarowar
Report prepared by	Chowdhury Sarowar

Notes:

Sample: shs-4-09

Full spectrum

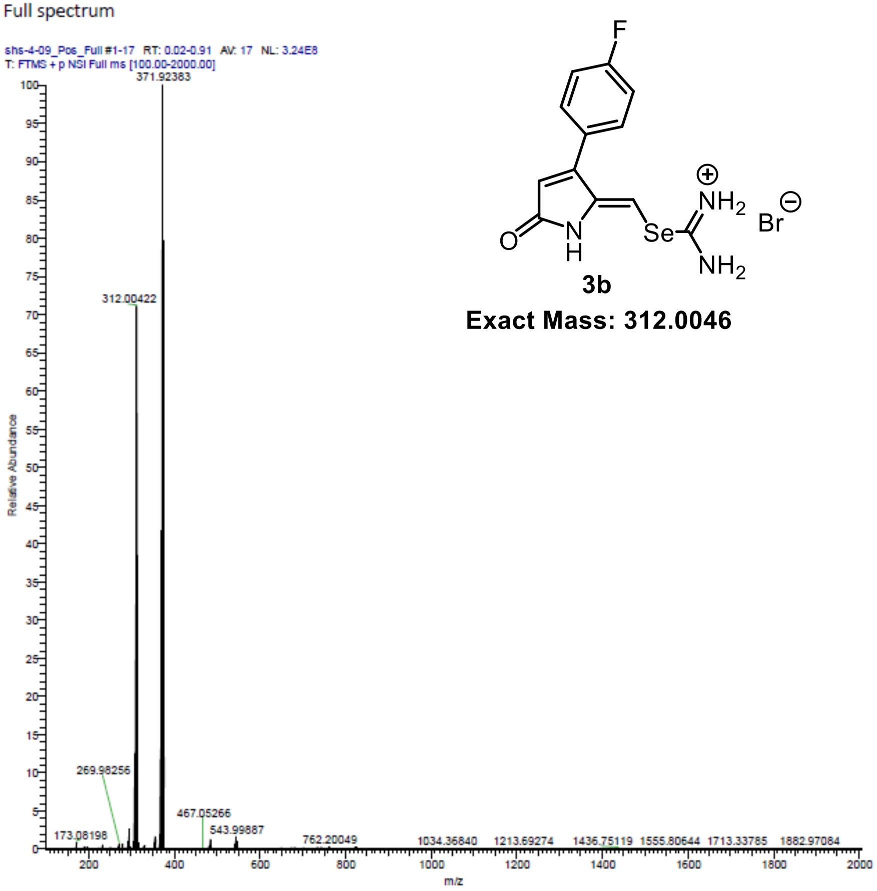

3b

Exact Mass: 312.0046

Sample submission report

Sample Analysis Request # **3073456**
Samples submitted by **Shekh Sabir**
Date run 2020-11-17
Operator Chowdhury Sarowar
Report prepared by Chowdhury Sarowar

Notes:

Sample: shs-3-112

Full spectrum

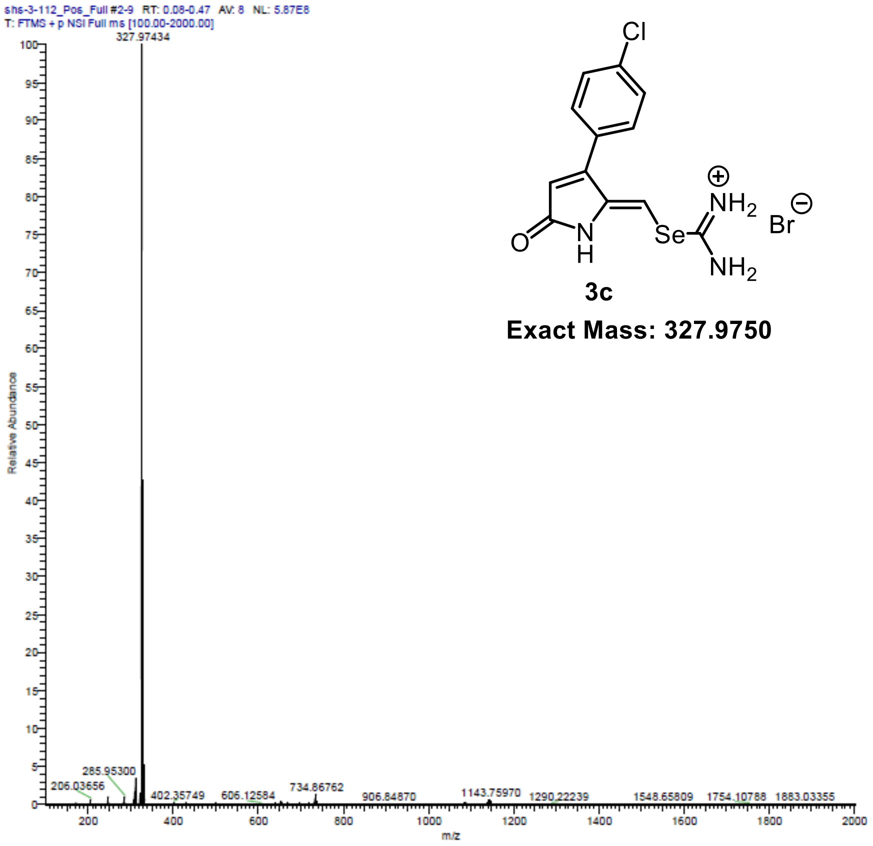

3c

Exact Mass: 327.9750

Sample submission report

Sample Analysis Request # **3073456**
Samples submitted by **Shekh Sabir**
Date run 2020-11-17
Operator Chowdhury Sarowar
Report prepared by Chowdhury Sarowar

Notes:

Sample: shs-3-100

Full spectrum

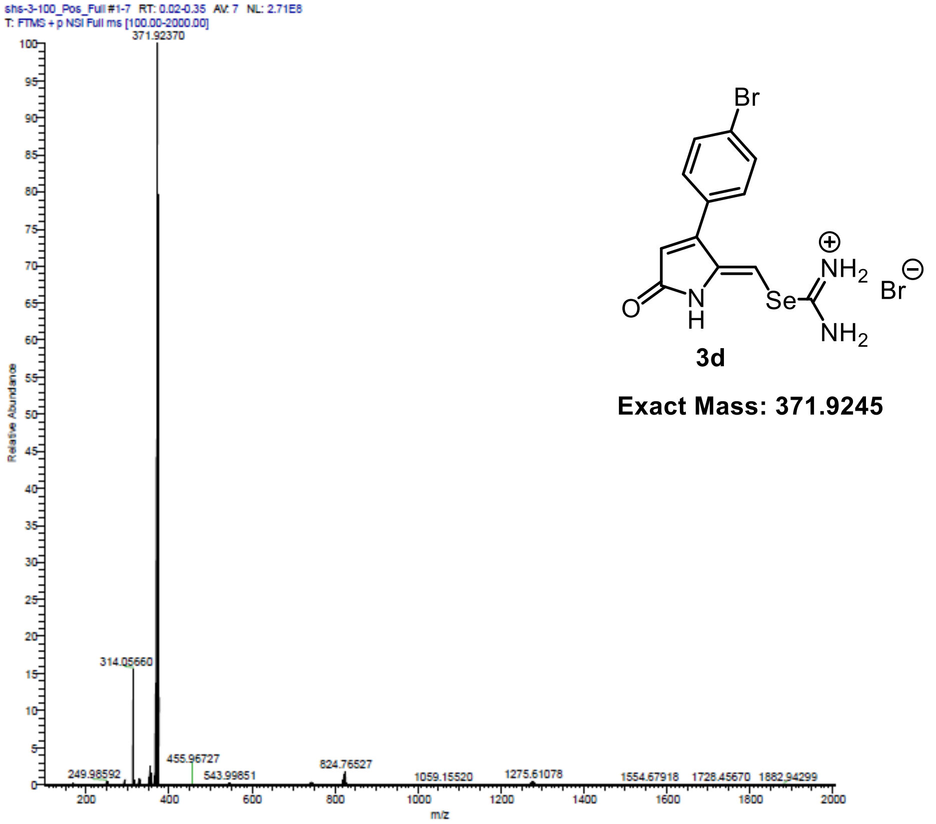

3d

Exact Mass: 371.9245

Sample submission report

Sample Analysis Request #	**2997593**
Samples submitted by	**Sabir, Shekh**
Date run	2020-08-11
Operator	Chowdhury Sarowar
Report prepared by	Chowdhury Sarowar

BMSF Bioanalytical Mass Spectrometry Facility

Notes:

Sample: shs-3-24

Full spectrum

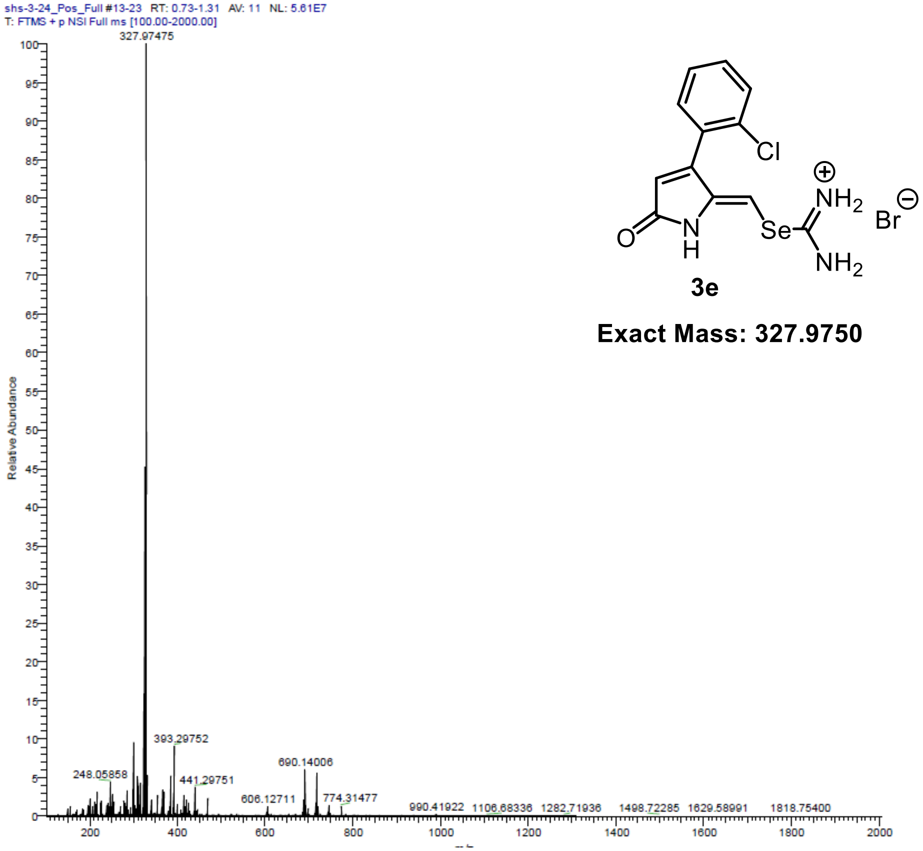

3e

Exact Mass: 327.9750

Sample submission report

Sample Analysis Request #	**3073456**
Samples submitted by	**Shekh Sabir**
Date run	2020-11-17
Operator	Chowdhury Sarowar
Report prepared by	Chowdhury Sarowar

Notes:

Sample: shs-4-19

Full spectrum

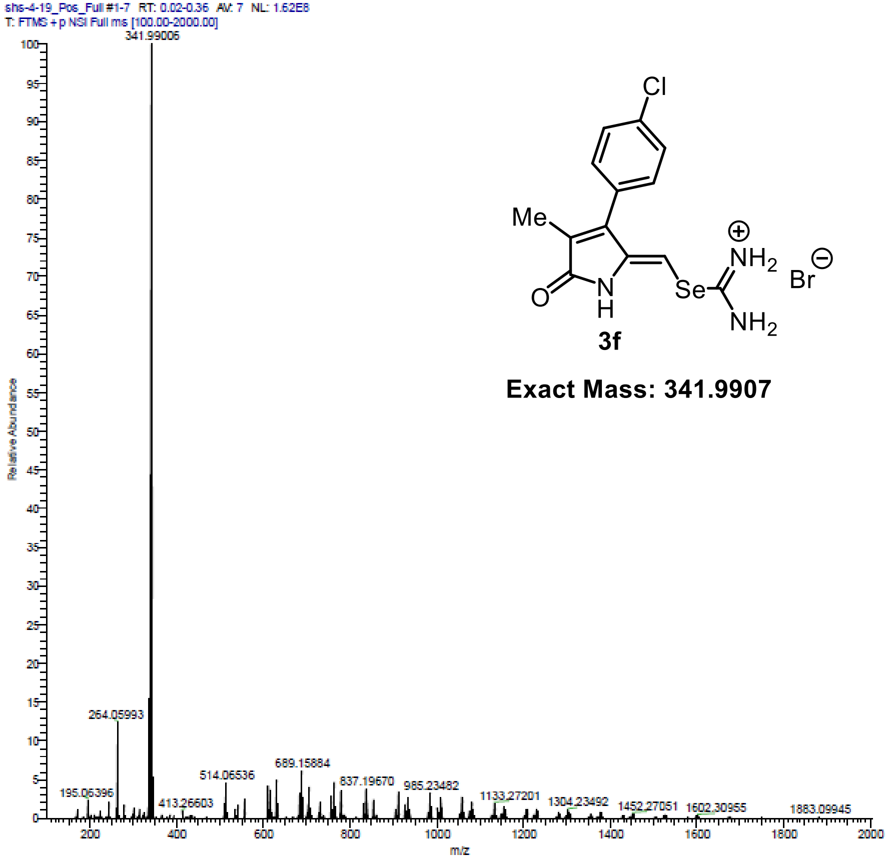

3f

Exact Mass: 341.9907

Sample submission report

Sample Analysis Request #	**3073456**
Samples submitted by	**Shekh Sabir**
Date run	2020-11-17
Operator	Chowdhury Sarowar
Report prepared by	Chowdhury Sarowar

BMSF Bioanalytical Mass Spectrometry Facility

Notes:

Sample: shs-3-103

Full spectrum

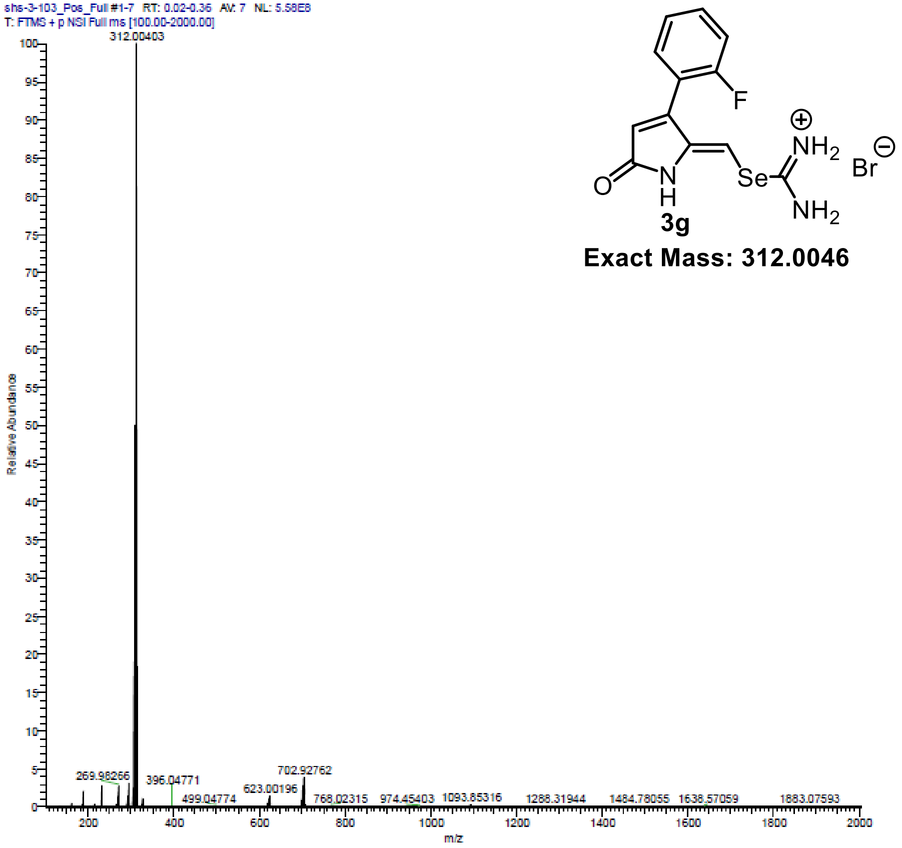

3g

Exact Mass: 312.0046

Sample submission report

Sample Analysis Request #	**3073456**
Samples submitted by	**Shekh Sabir**
Date run	2020-11-17
Operator	Chowdhury Sarowar
Report prepared by	Chowdhury Sarowar

Notes:

Sample: shs-4-14

Full spectrum

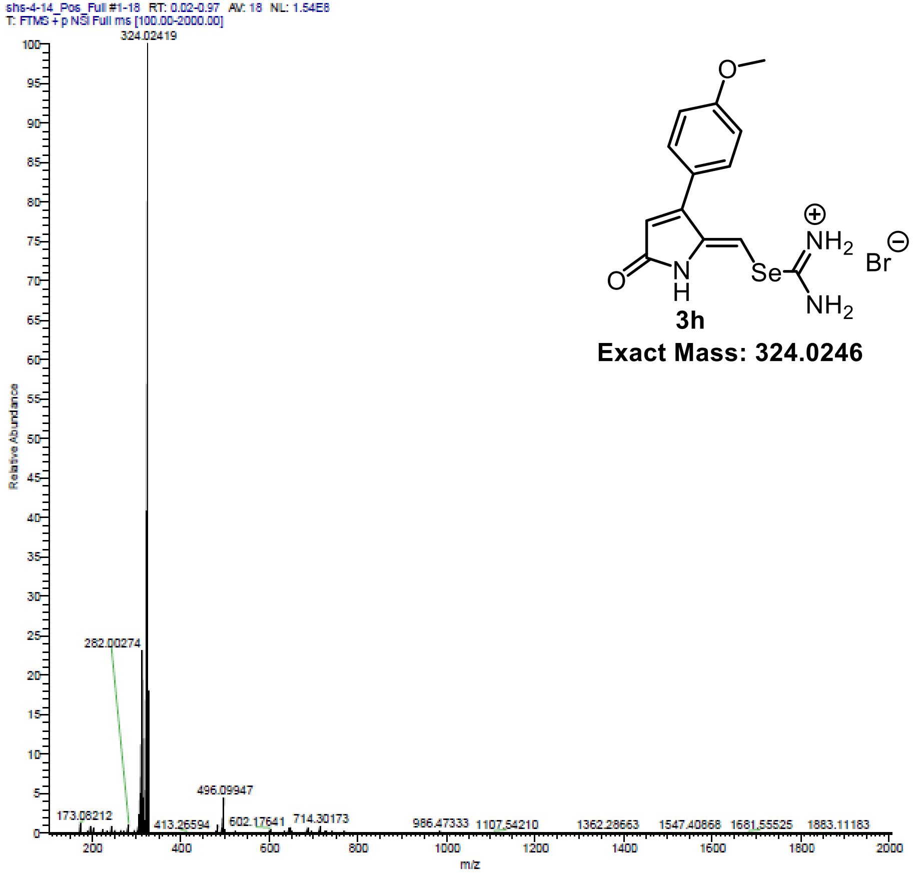

3h

Exact Mass: 324.0246

Sample submission report

Sample Analysis Request #	**3073456**
Samples submitted by	**Shekh Sabir**
Date run	2020-11-17
Operator	Chowdhury Sarowar
Report prepared by	Chowdhury Sarowar

Notes:

Sample: shs-3-115

Full spectrum

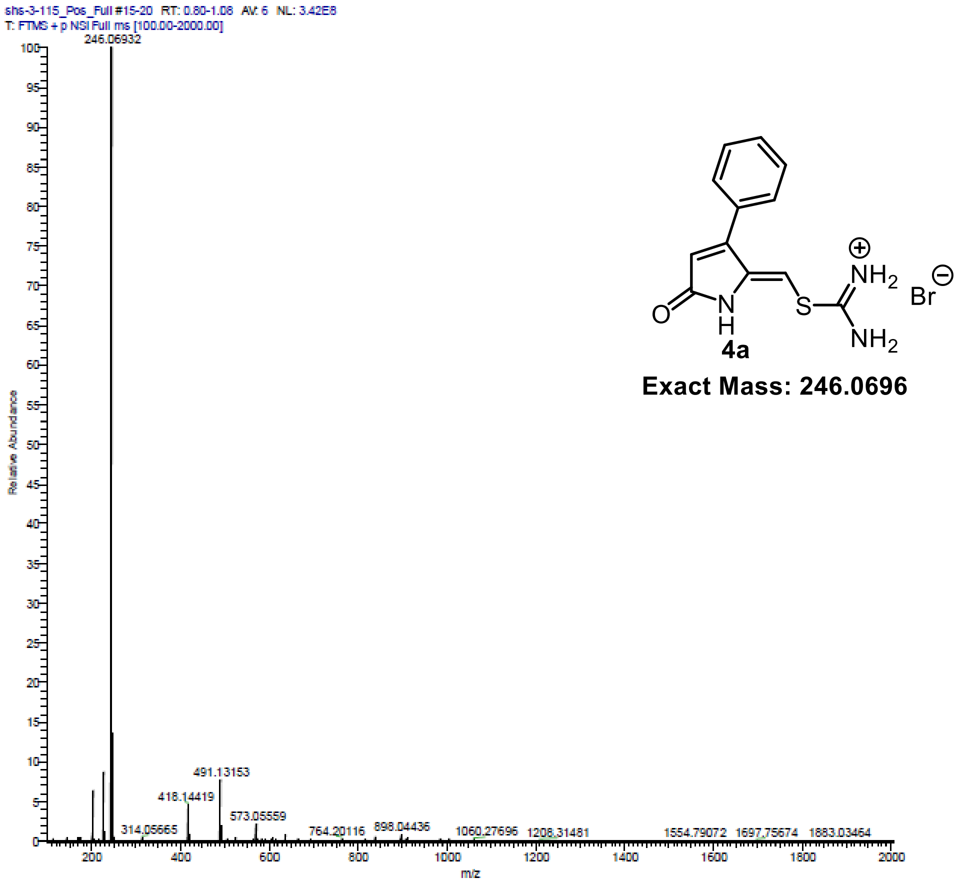

4a

Exact Mass: 246.0696

Sample submission report

Sample Analysis Request #	**3073456**
Samples submitted by	**Shekh Sabir**
Date run	2020-11-17
Operator	Chowdhury Sarowar
Report prepared by	Chowdhury Sarowar

Notes:

Sample: shs-4-10

Full spectrum

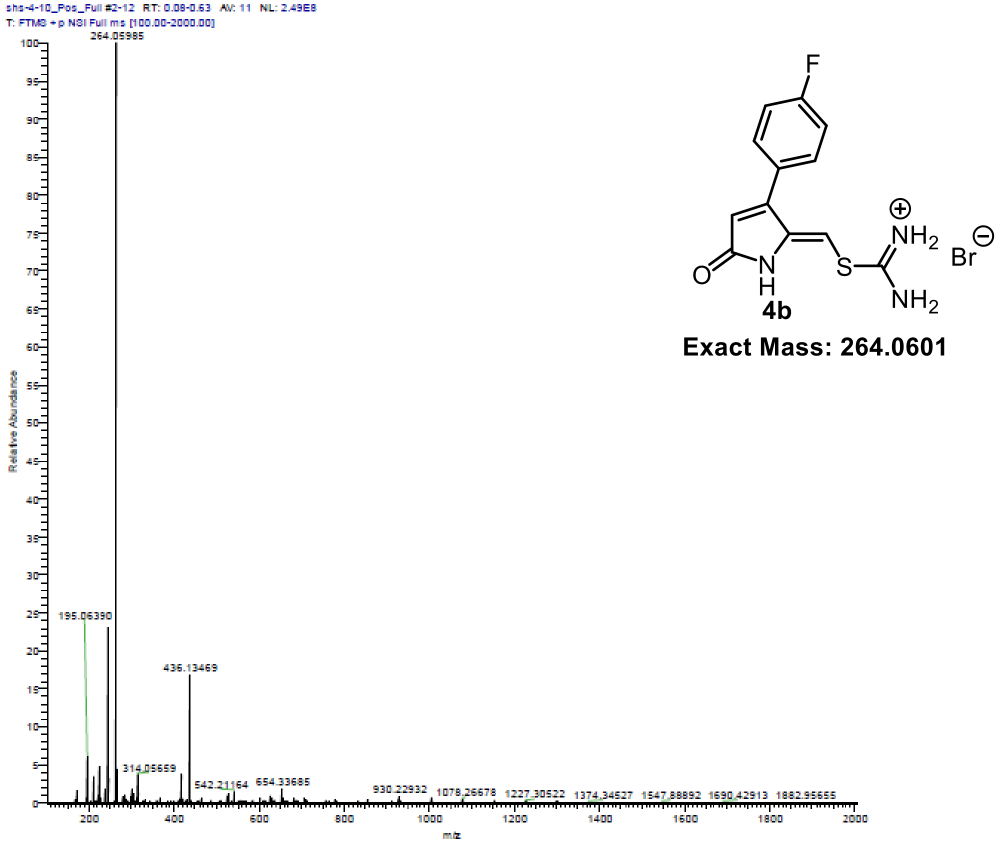

4b

Exact Mass: 264.0601

Sample submission report

Sample Analysis Request # **3073456**
Samples submitted by **Shekh Sabir**
Date run 2020-11-17
Operator Chowdhury Sarowar
Report prepared by Chowdhury Sarowar

Notes:

Sample: shs-4-06

Full spectrum

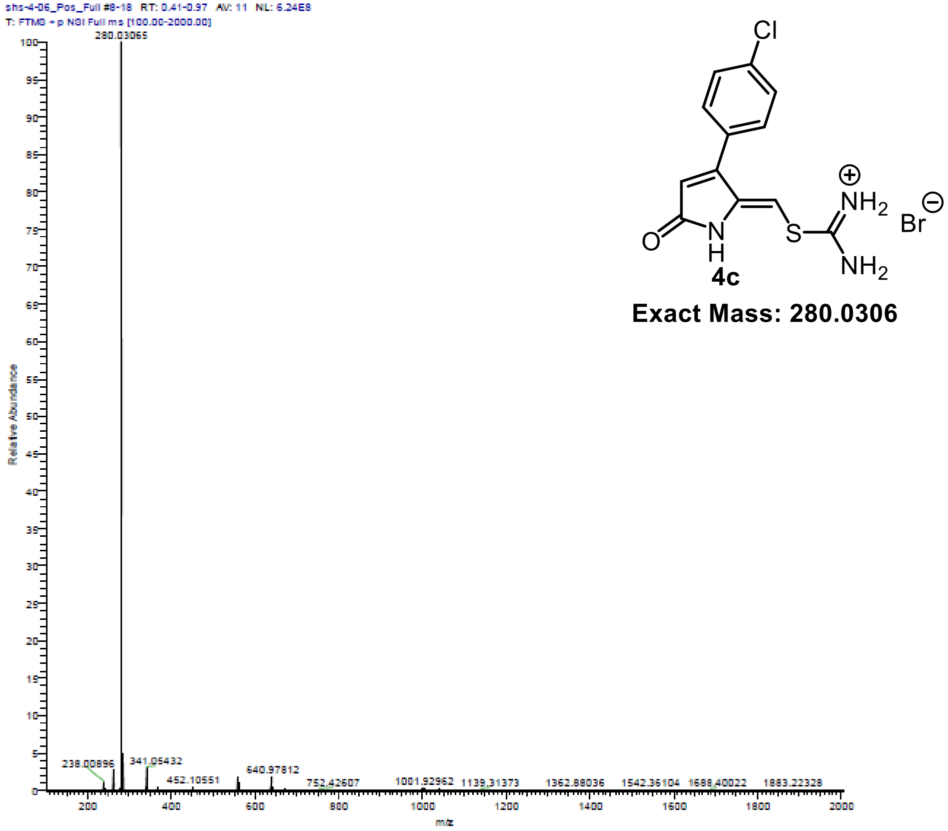

4c

Exact Mass: 280.0306

Sample submission report

Sample Analysis Request # **3073456**
Samples submitted by **Shekh Sabir**
Date run 2020-11-17
Operator Chowdhury Sarowar
Report prepared by Chowdhury Sarowar

Notes:

Zoomed spectrum

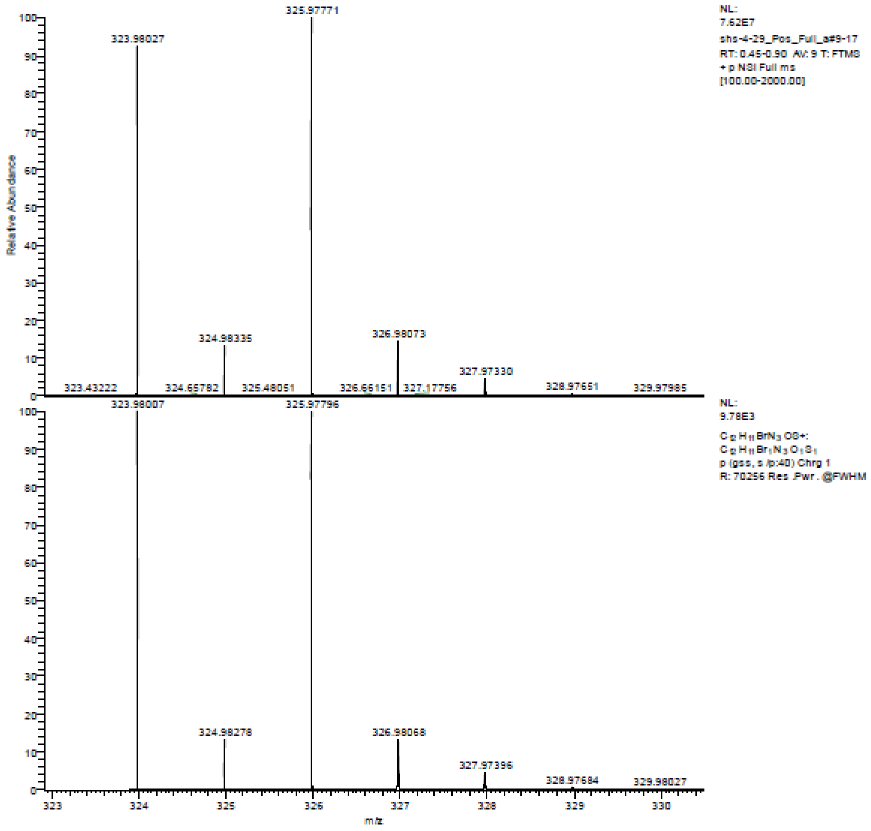

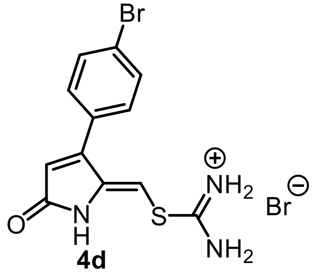

4d

Exact Mass: 323.9801

Sample submission report

Sample Analysis Request # **3073456**
Samples submitted by **Shekh Sabir**
Date run 2020-11-17
Operator Chowdhury Sarowar
Report prepared by Chowdhury Sarowar

Notes:

Sample: shs-3-107

Full spectrum

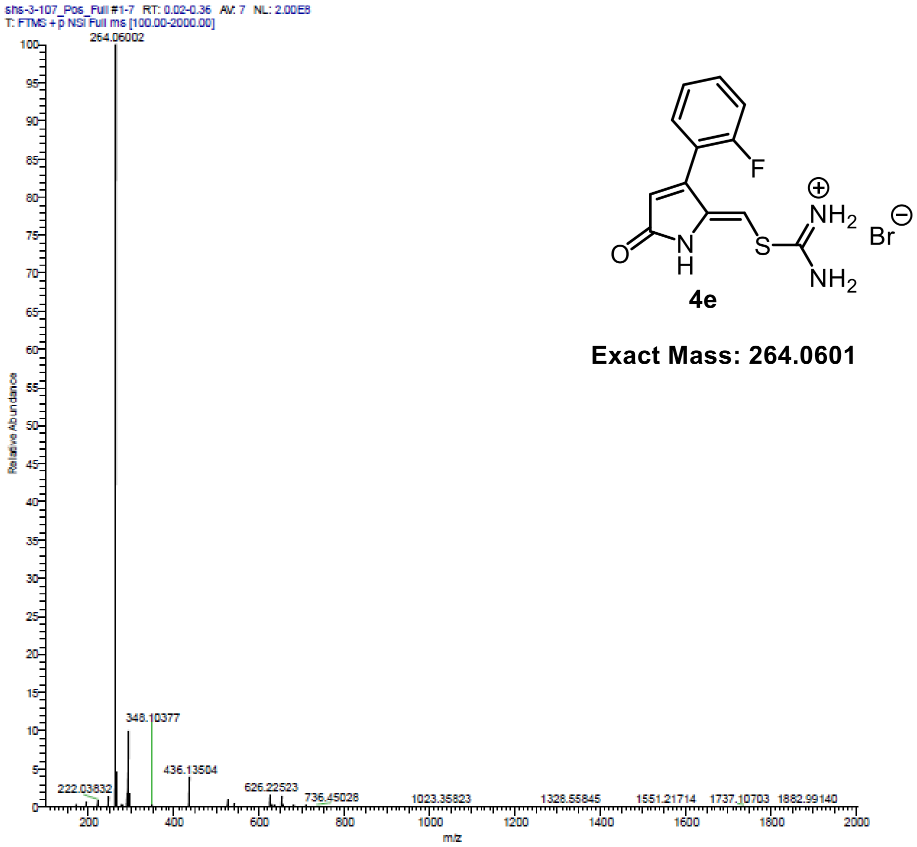

4e

Exact Mass: 264.0601

Sample: shs-4-25

Full spectrum

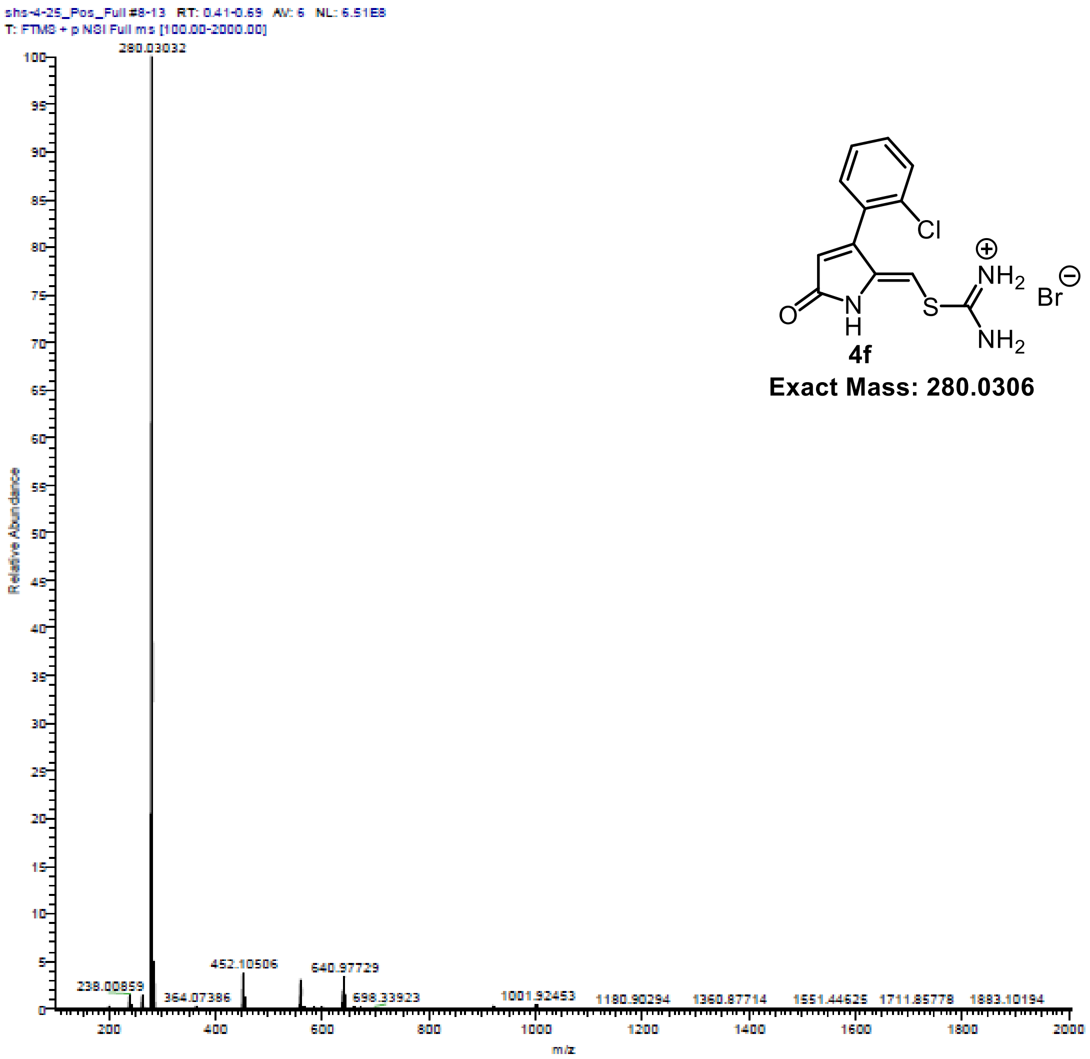

4f

Exact Mass: 280.0306

Sample submission report

BMSF
Bioanalytical Mass Spectrometry Facility

Sample Analysis Request #	**3073456**
Samples submitted by	**Shekh Sabir**
Date run	2020-11-17
Operator	Chowdhury Sarowar
Report prepared by	Chowdhury Sarowar

Notes:

Sample: shs-3-26

Full spectrum

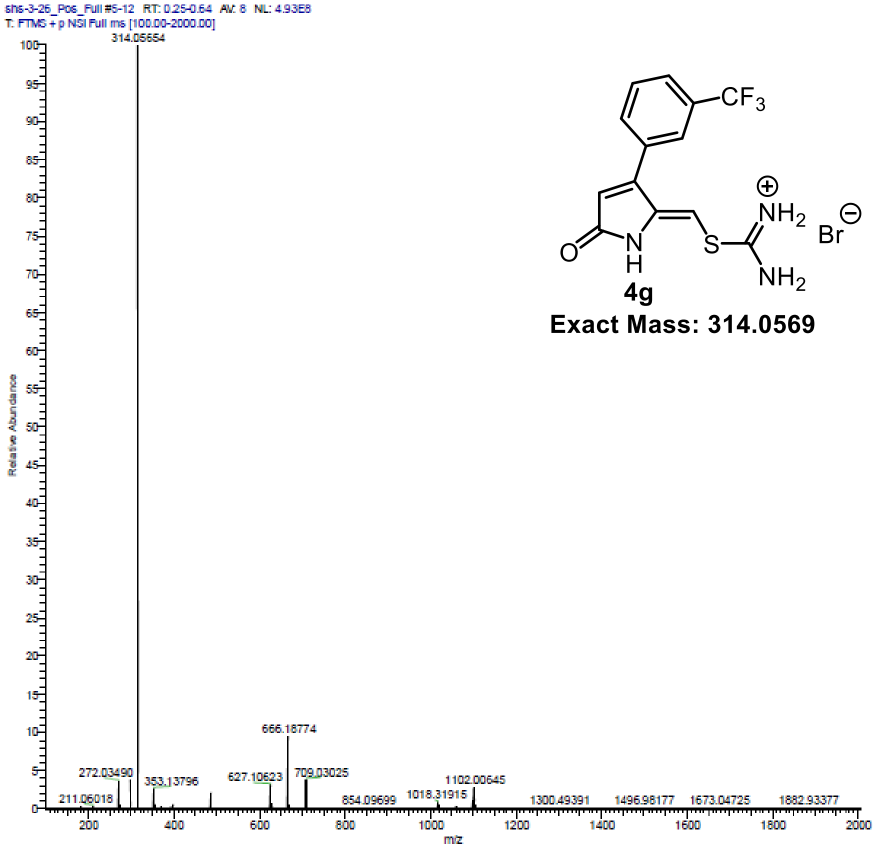

4g

Exact Mass: 314.0569

Agilent Resolutions Pro

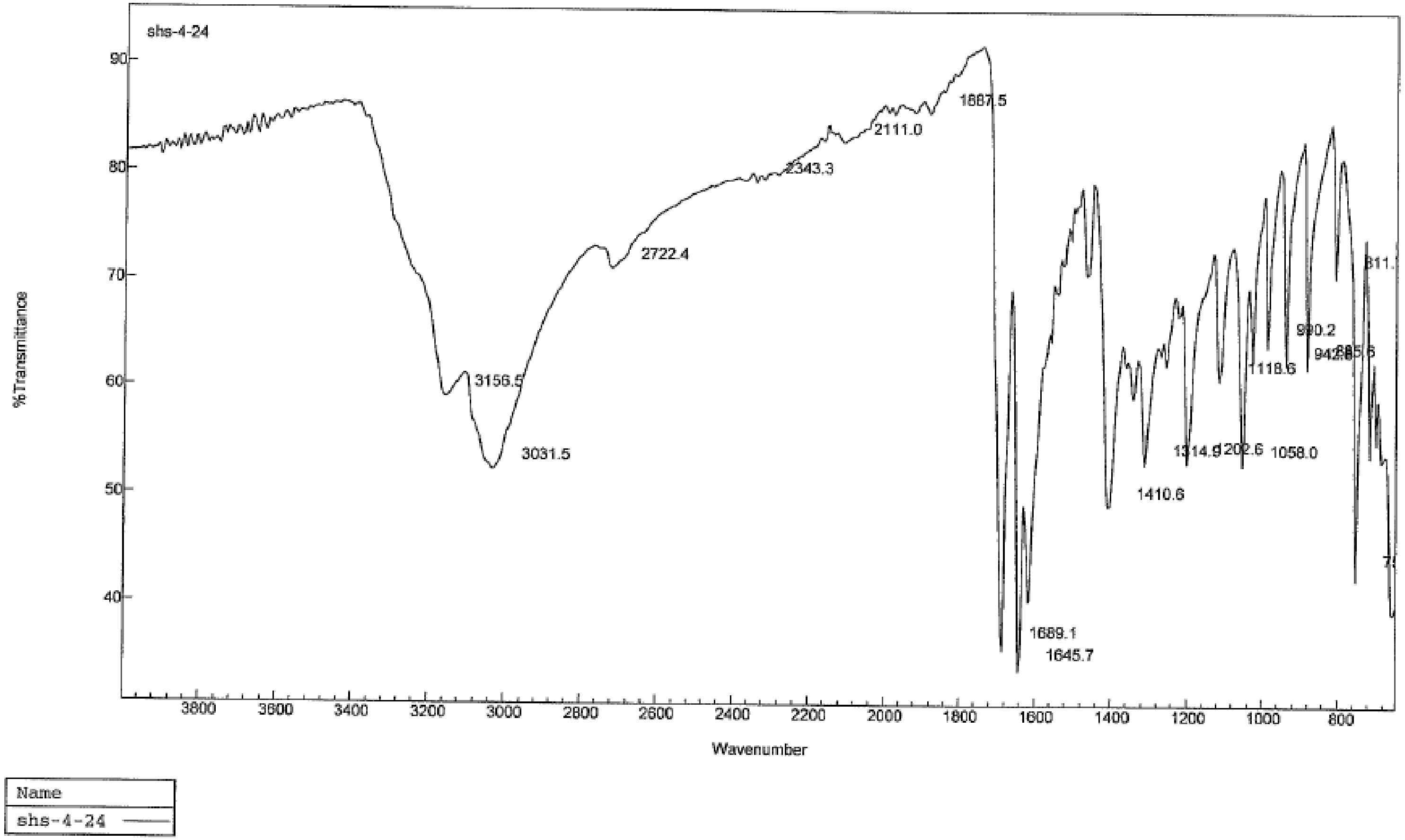

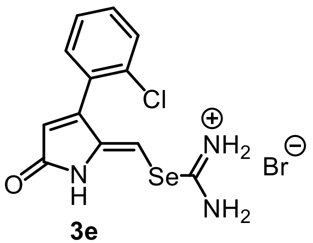

3e

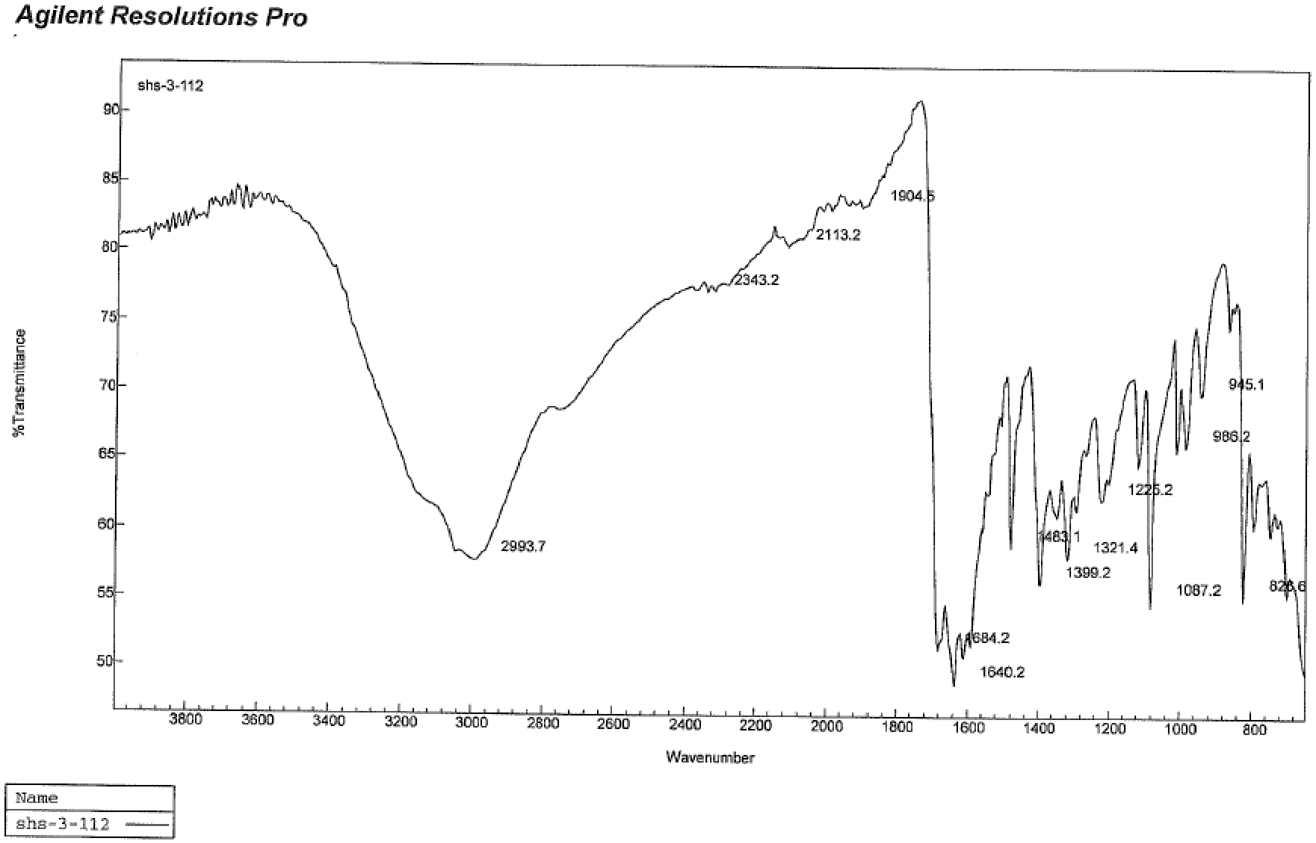
Agilent Resolutions Pro
shs-3-112
%Transmittance
Wavenumber
2993.7
2343.2
2113.2
1904.5
1684.2
1640.2
1463.1
1399.2
1321.4
1225.2
1087.2
986.2
945.1
826.6
Name
shs-3-112

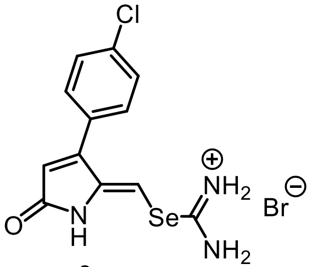
Cl
O
N
H
Se
NH2
NH2
Br

3c

Agilent Resolutions Pro

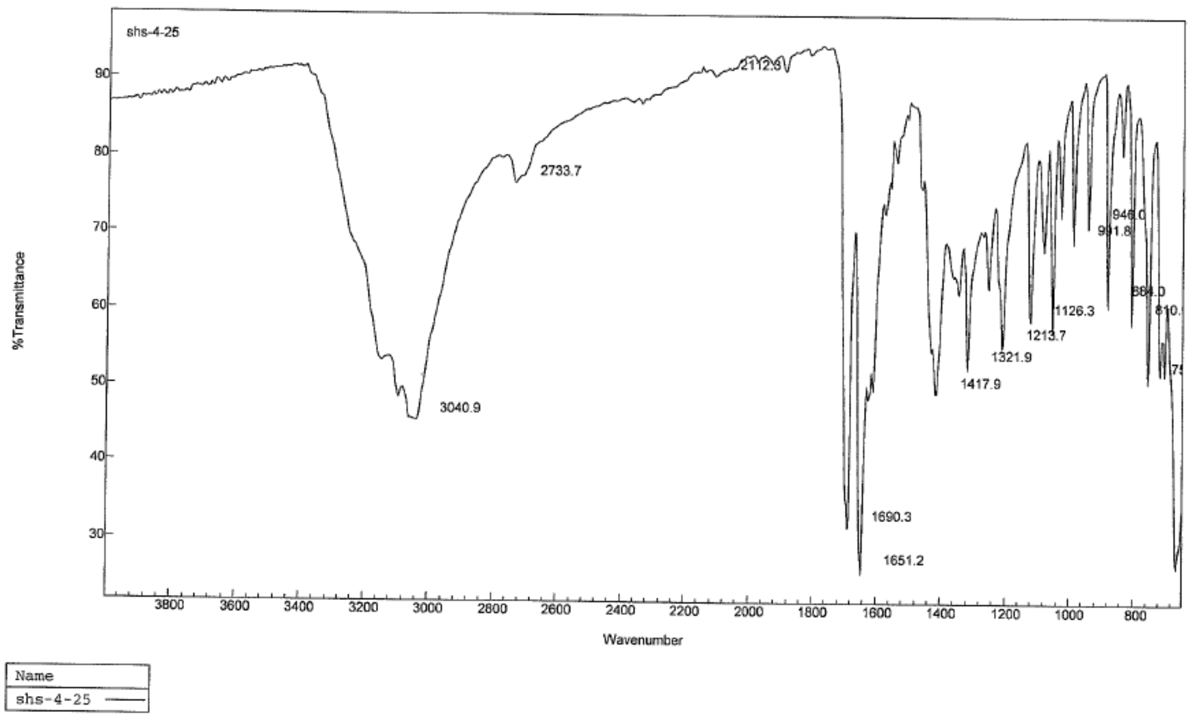

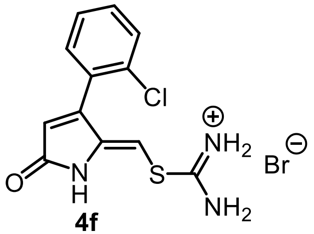

4f

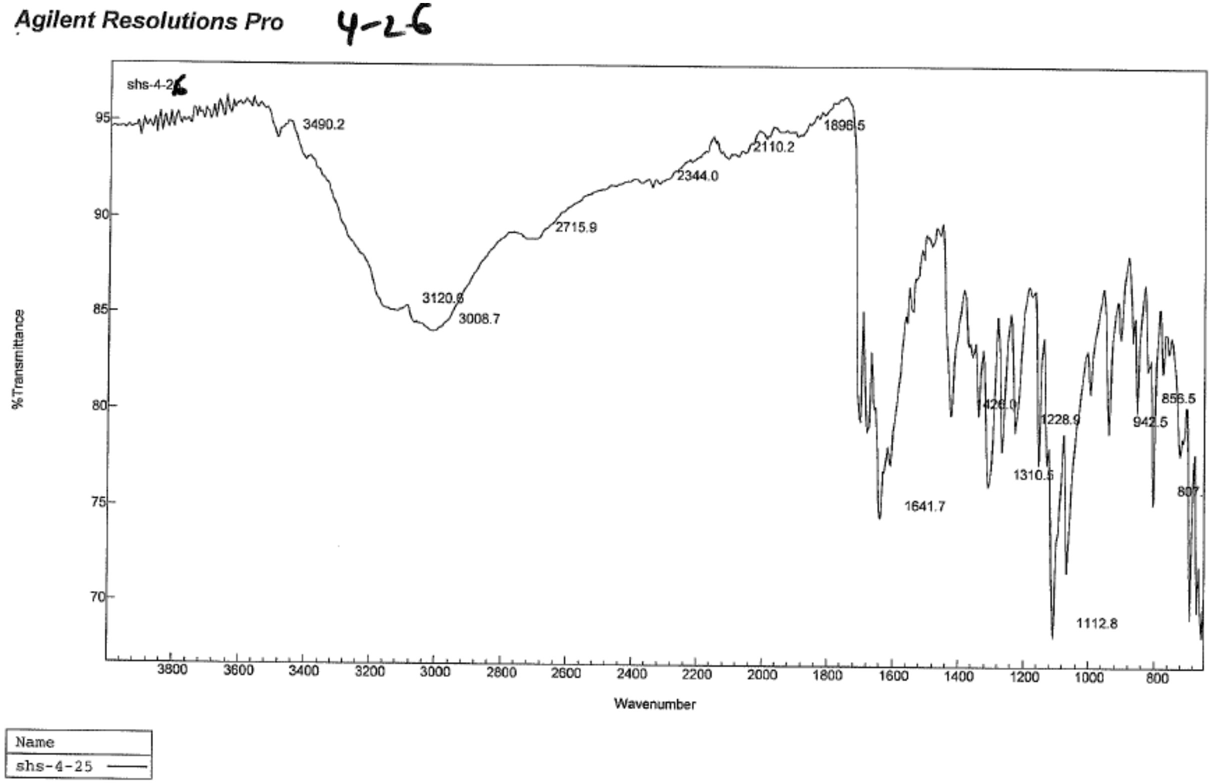
Agilent Resolutions Pro
4-26
shhs-4-26
3490.2
3120.6
3008.7
2715.9
2344.0
2110.2
1896.5
1641.7
1426.0
1310.5
1228.9
1112.8
942.5
856.5
807.
%Transmittance
Wavenumber
Name
shs-4-25

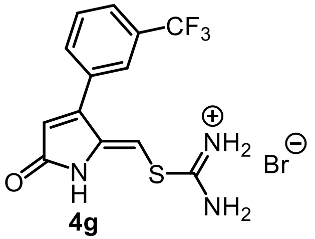
CF3
NH2
Br
O
N
H
S
NH2

4g

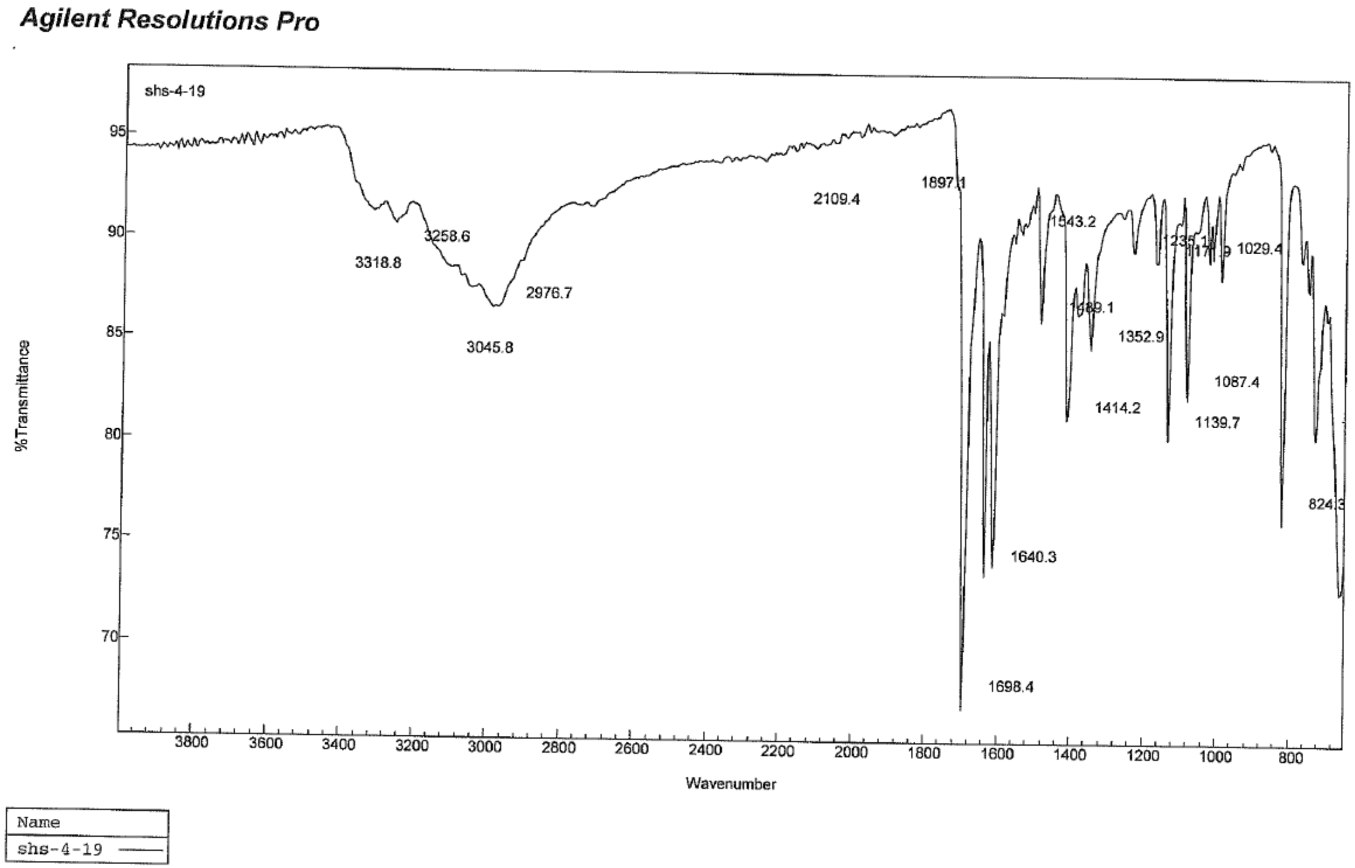
Agilent Resolutions Pro
shs-4-19
95
90
85
80
75
70
%Transmittance
3318.8
3258.6
3045.8
2976.7
2109.4
1897.1
1543.2
1389.1
1352.9
1235.1
1029.4
1087.4
1414.2
1139.7
824.3
1640.3
1698.4
3800
3600
3400
3200
3000
2800
2600
2400
2200
2000
1800
1600
1400
1200
1000
800
Wavenumber
Name
shs-4-19

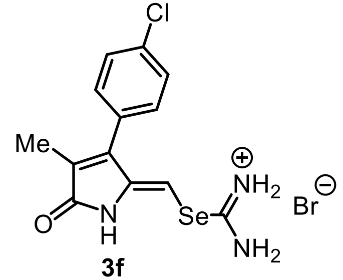
Cl
Me
O
N
H
Se
NH2
NH2
Br

3f

Effect on bacterial growth

Compound	**% Growth inhibition data** (*P. aeruginosa* MH602)		
	125 µM	61.5 µM	31 µM
3a	49.8±7.6	33.7±7.6	19.2±3.6
3b	26.9±3.4	12.4±1.8	7.9±7.2
3c	41.1±6.8	32.2±2.7	22.8±3.3
3d	39.5±2.7	31.8±3.3	16.7±3.0
3e	36.2±7.5	25.8±4.6	10.5±4.5
3f	23.7±3.4	12.4±1.8	7.9±7.2
3g	32.1±6.8	21.3±2.8	9.2±8.1
3h	26.9±14.4	21.8±3.2	1.3±1.13
4a	18.9±11.3	7.7±4.1	3.3±2.3
4b	10.4±9.1	4.4±4.4	0.0±0.0
4c	30.0±3.9	19.5±2.3	10.6±7.3
4d	34.9±2.2	25.3±7.9	15.2±4.0
4e	55.6±3.4	43.4±6.9	30.0±2.2
4f	33.3±7.2	33.9±5.7	33.1±5.8
4g	58.9±5.5	46.8±4.3	36.0±8.9
Fu 30	50.2±3.4	40.5±21.9	22.8±8.1
